# Characterization of metagenome-assembled genomes of two endo-archaea of *Candida tropicalis*

**DOI:** 10.3389/frmbi.2022.1020341

**Published:** 2023-02-01

**Authors:** Uppada Jagadeeshwari, Chintalapati Sasikala, Anusha Rai, B. Indu, Sahu Ipsita, Chintalapati Venkata Ramana

**Affiliations:** ^1^ Bacterial Discovery Laboratory, Centre for Environment, Institute of Science and Technology (IST), Jawaharlal Nehru Technological (JNT) University Hyderabad, Hyderabad, India; ^2^ Department of Plant Sciences, School of Life Sciences, University of Hyderabad, Hyderabad, India

**Keywords:** Metagenome-assembled genomes (MAG), endo-archaea, *Heimdallarchaeota*, *Candida tropicalis*, *Asgardarchaeota*

## Abstract

**Introduction:**

Host-microbe interactions are pivotal in host biology, ecology, and evolution. Recent developments in sequencing technologies have provided newer insights into the same through the hologenome concept.

**Methods:**

We report here the study on metagenome-assembled genomes (MAGs) associated with *Candida tropicalis* (studied through shotgun metagenome sequencing), adding to the knowledge about endomicrobiomes of yeast. De novo assembly and binning recovered two partial archaeal genomes, taxonomically belonging to the phylum Asgardarchaeota.

**Results and Discussion:**

The phylogenomic analysis based on the core genes revealed that both the binned genomes cladded separately with the less studied and uncultivated ‘*Candidatus*’ superphylum, designated as *Asgard* archaea (the nearest known relative of eukaryotes). Between the two binned genomes, the average nucleotide index (ANI) was 71.2%. The average nucleotide identities (ANI) of the two binned genomes with ‘*Candidatus* Heimdallarchaeota’ were 60.4-61.2%. The metabolic pathways of both the binned genomes predicted genes belonging to sulfur reduction, Kreb’s pathway, glycolysis, and C1 carbon metabolism. Further, both the binned genomes were predicted to support autotrophic as well as the heterotrophic mode of growth, which might probably help the host in its nutritional requirements also. Further, the genomes showed few eukaryotic signature proteins (ESPs) and SNARE proteins indicating that members of *Asgardarchaeota* are the closest relatives of eukaryotes. The gaps present in the metabolic potential of the MAGs obtained and the absence of a few essential pathways shows that they are probably in a symbiotic relationship with the host. The present study, reports for the first-time endosymbiosis of *Asgard* archaea with yeast. It also provides insights into the metabolic potential, ecology, evolutionary history, and endosymbiotic nature of the important but 160 poorly studied *Asgard* archaea.

## Introduction

The availability of low-cost techniques for analyzing microbial communities has caused a remarkable increase in the studies related to ecology, diversity, and evolution of microbiome-host systems (Carrier and Reitzel, 2017; Uhr et al., 2019). Further, the general interest in the concomitant relationship between the microbe and host has been expanding, leading to the generation of the hologenome theory of evolution (Zilber-Rosenberg and Rosenberg, 2008). The hologenome theory states that plants and animals, in association with their microbiome, serve as an entity for evolution. The term “hologenome” denotes the total gene constituents of the holobionts (Rosenberg et al., 2007), whereas holobionts are the hosts (plant/animal), along with the microorganisms dwelling as exo-symbionts or endosymbionts (Margulis, 1991). This evolutionary concept vividly applies to the eukaryotic systems of plants, animals, and humans (Alberdi et al., 2016; Rosenberg and Zilber-Rosenberg, 2016); however, its implications on specific model microorganisms like yeast are yet to be explored.

The role of archaea and bacteria in endosymbiosis with eukaryotes is one of the most long-lasting mysteries in modern biology. There are still debates going on from past decades on the concept of symbiotic origin of eukaryotes from bacteria and archaea (Baum, 2015; Martin et al., 2015; Dey et al., 2016). Majority of the bacteria and archaeal endosymbionts inhabit the cytoplasm whereas some appear to be distributed freely, swimming or surrounded by host-derived membranes. Most of the studies conducted on endosymbiosis so far were associated with bacterial members with other organisms and only a few cases are reported about endosymbiosis between Archaea and other organisms. These mainly include methanogenic archaeal endosymbionts with eukaryotes like anaerobic protozoa, free-living ciliates, sponges, molluscs and even humans (*Methanobrevibacter smithii*). Studies on how archaea may interact with the eukaryotic host is very limited due to the absence of model organisms and much more work needs to be done to understand the association of archaea with the eukaryotic host (Hoppert et al., 2013).

The discovery of *Asgard* archaea has boosted the eocyte hypothesis that eukaryotes evolved from archaea as *Asgard* archaea possess two remarkable features: robust evolutionary affinity with eukaryotes and the existence of various eukaryotic signature proteins (ESPs) (Wu et al., 2022). The phylogenetic analyses of eukaryotes and other nearest members from other domains have suggested that the closest archaeal sister lineage to eukaryotes may be the proposed super phylum, *Asgard* archaea that consist of *Lokiarchaeota, Thorarchaeota, Odinarchaeota*, and *Heimdallarchaeota* (Spang et al., 2022). The members of *Heimdallarchaeota* are identified as the *Asgard* archaeal lineage to the eukaryotic branch based on various phylogenetic, and phylogenomic, analyses based on conserved protein sequences (Xie et al., 2021). A recent study by Neveu et al. (2020) has shown that SNARE-like proteins were also present in the *Asgard* archaea. Their interactive study of forming stable complexes between eukaryotic SNAREs and archaeal proteins was monumental in suggesting that the diverse eukaryotic SNARE proteins evolved from archaea, the closest known living relative of eukaryotes (Neveu et al., 2020).

So far, the genetic and metabolic potential of the members of *Heimdallarchaeotes* were only inferred from analysis of MAGs that are fragmented and suffer from uncertainty in their completeness and accuracy caused during MAG assembly and binning (Wu et al., 2022). These drawbacks resulted in uncertainties regarding the relationship of archaea with eukaryotes in terms of endosymbiosis and eukaryogenesis (Xie et al., 2021). Yeasts are unicellular eukaryotic microorganisms that have served as model systems for several molecular-based studies. This is attributed to their unicellular organization and similarity to the other eukaryotic systems in cell biological pathways (Mohammadi et al., 2015; Heinisch and Brandt, 2016). In addition, they are amenable to genetic manipulation and thus for testing hypotheses. Recent studies have shown that yeasts are rich reservoirs of endobacteria (Deveau et al., 2018; Indu et al., 2021). Although bacteria-fungi interactions are well studied and tested (Hoffman et al., 2013; Uehling et al., 2017), the same is yet to be carried out in unicellular fungi like yeasts. The functional role of endobacteria in yeast is yet to be inferred; however, several studies in recent years have shown their effects on host dynamics. It includes functions related to nitrogen fixation (Sen et al., 2021) and distribution in specialized niches (Salmanian et al., 2012). However, there are no reports so far on the endosymbiotic relationship of archaeal members and yeast as host.

In the present study, the shotgun metagenome approach was applied to expand knowledge on the yeast microbiome and its occurrence inside the cells of the yeast, *Candida tropicalis*. With advanced sequencing and bioinformatics methods, genomes of microorganisms can be acquired even without the cultivation requirement. With shotgun metagenome, several metagenome-assembled genomes (MAGs) have been recovered from diverse samples (Chen et al., 2021; Nayfach et al., 2021). MAGs approaches can recover populations-variable genes and core genes, which can further assist in providing valuable insights related to population genetics, microbial-host interactions, and their survival strategies (Meziti et al., 2021). The present study attempts to extend existing information (Indu et al., 2021) on prokaryote-yeast interaction with the fundamental approach of using metagenome-assembled genomes (MAGs).

## Materials and methods

### Organism and growth conditions

Details of isolation and identification of *Candida tropicalis* strain JY101 are given by Indu et al. (2021). The organism was grown on yeast peptone dextrose (YPD) agar (HIMEDIA, M1363) at 30°C with an incubation period of 4 to 5 days. The culture was preserved as glycerol stock (50% v/v) at -20°C.

### DNA extraction, shot-gun sequencing and data processing

Before the DNA extraction, the purity of the yeast culture was checked by observing yeast cells under a phase contrast microscope and under the confocal microscope after staining the cells with DAPI (4′,6-diamidino-2-phenylindole) as mentioned by Indu et al., 2021. The genomic DNA of *C. tropicalis* was isolated from cells grown in YPD broth (at 30°C for five days and harvested by centrifuging at 6000xg for 10 min) by following the manufacturer’s protocol of NucleoSpin Kit. DNA was stored at -20°C until further use. The shotgun metagenome sequencing of whole-genome DNA was outsourced to Nucleome Informatics, Hyderabad, and carried out using the Illumina High Throughput sequencer (Illumina HiSeq 2500). The DNA libraries were constructed through the process of personalized MetaFast – Ligation on Amplicon which constitutes end pairing, adding A to tail, purification, and PCR amplification. The qualified libraries, after pooling according to their effective concentration and expected data volume, were fed into the sequencer for 2*150 bp sequencing. The paired-end raw reads have been submitted to the NCBI database. The raw reads were then imported into KBase (https://www.kbase.us/) for further analysis using the applications available in KBase (Arkin et al., 2018). The quality of raw reads was assessed using FastQC (Andrews, 2017; http://www.bioinformatics.babraham.ac.uk/projects/fastqc). Quality trimming and adapter removal were performed using the default parameters of Trimmomatic v0.36 (Bolger et al., 2014; Andrews, 2017). The trimmed reads were then assembled using the *de novo* assembler metaSPAdes v3.13.0 with a minimum contig length of 300 and the resulting assembly was used for further binning (Nurk et al., 2017). The assemblies were aligned using bowtie for the detection of artefacts due to sequencing (Lind et al., 2018). The taxonomic classification of the metagenome in order to understand the microbiome associated with the host was carried by Kaiju by using the NCBI nr database containing both eukaryotes and prokaryotes (Menzel et al., 2016) and the results were viewed using the Krona graph (Ondov et al., 2011). The assembled contigs were binned using three computational approaches of MaxBin2 v2.2.4, MetaBAT2 Contig Binning v1.7 and CONCOCT v1.1 present in KBase (Alneberg et al., 2014; Wu et al., 2016; Kang et al., 2019). The MaxBin2 clusters assembled contiguous genome fragments of metagenomic sequence into different “bins”, each of which corresponds to a putative population genome. It uses nucleotide composition information, source strain abundance (measured by depth-of-coverage by aligning the reads to the contigs), and phylogenetic marker genes (40 marker gene sets that are universal among bacteria and archaea) to perform binning through an Expectation-Maximization (EM) algorithm with a probability threshold of 0.8 (Wu et al., 2016). The binning app CONCOT- v1.1 uses Bowtie for read mapping based on nucleotide composition and depth of coverage (Alneberg et al., 2014). The MetaBAT calculates their pairwise probabilistic integrated composite distances based on tetranucleotide frequency (TNF) and abundance later supplied to a modified k-medoid clustering algorithm to bin contigs iteratively and exhaustively into genome bins (Kang et al., 2019). The refinement and optimization of binned contigs by consensus resulted in the generation of two binned genomes. The refinement was performed using DAS Tool - v1.1.2, an automated method that optimizes bacterial or archaeal genome bins using a de-replication, aggregation and scoring strategy by using diamond for single copy gene identification. The bins constructed were extracted as assemblies using Extract Bins from Binned Contigs v1.0.2 app available in KBase (Arkin et al., 2018). CheckM was used to analyze the genome quality and completeness and to detect contamination (Parks et al., 2015). The downloaded metagenome-assisted genome (MAG) assemblies were used for further downstream analysis. The assemblies were deposited in NCBI as Bin 001 and Bin 002.

### Genome annotation and phylogenetic studies

The phylogenetic-based taxonomic classification of the reconstructed MAGs was performed using Genome Taxonomy Database (GTDB)-Tk Classify-v1.6.0 using single-copy phylogenetic markers (Chaumeil et al., 2020). MAGs were preliminarily screened for their phylogenetic assignment using PhyloPhlAnV 3.0 (Asnicar et al., 2020) with universal markers from GTDB as a reference database. The phylogenomic tree was based on core genes and was constructed by using an Up-to-date bacterial core gene set (UBCG) to assess further the taxonomic position (Na et al., 2018). The average amino acid identity (AAI) of assembled binned genomes with respect to its nearest members as placed by the GTDB reference tree was calculated using the AAI calculator by Kostas lab (Rodriguez-R and Konstantinidis, 2016). The genome annotation was performed using the RAST tool kit (RASTtk) (https://rast.nmpdr.org/rast.cgi), Patric (Wattam et al., 2017) and NCBI PGAP (Prokaryotic genome annotation pipeline) (Aziz et al., 2008; Tatusova et al., 2016). The EC numbers of the protein-coding genes were downloaded from Patric webserver and then submitted to the online interactive Pathways (iPath) explorer v3.0 (Darzi et al., 2018) for obtaining the differences between the metabolic constructions of the MAGs obtained.

### Data availability

The metagenome-assembled draft genomes of bins (Bin 001 and Bin 002) are available in NCBI GenBank under the following accession number of JAMCOH00000000 and JAMCOI00000000, respectively. Similarly, the paired-end raw shotgun sequence reads were submitted in the NCBI-SRA database under the accession number SRR19140378.

## Results

### An overview of the binned archaeal genomes and host-associated microbiome

The shotgun metagenome sequencing of *C. tropicalis* strain JY101 holobiome resulted in the generation of 83 million reads and 7.5 gigabases in total. The taxonomic classification of the shotgun metagenome with Kaiju shows that majority of the reads were belonging to *Eukaryota* (67%), followed by *Bacteria* (30%), *Archaea* (0.5%) and others. Among the *Archaea*, majority of the reads were belonging to *Euryarchaeota* (80%) followed by other members. The *Asgard* archaea were present in about 1% of the total archaeal reads (**Figure S1**).

The assembly of metagenomic reads into contigs with minimum contig length of 300 and k-mer size of 33 in metaSpades resulted in 528 contigs with a total length of 14.14 Mb and GC content of 33.01%. The L50 and N50 of the assembly constructed are 77 and 58640, respectively. The contigs formed were grouped and assigned into discrete population bins based on depth-of-coverage, nucleotide composition and marker genes. The bins formed were filtered by using CheckM resulting in two binned archaeal partial genomes. The process of binning did not result in contiguous genomes; however, the bins formed were close to the full set of genes from an organism and hence were used for functional studies. The most important parameters like assembly quality, estimates of genome completeness, and contamination were assessed to determine the quality of the MAGs obtained. The two metagenome-assembled genomes (MAGs) consisted of chromosomes of length 5,387,319 bp and 4,219,691 bp, with coverage of 50x. Bin 001 had a GC content of 34.4%, while Bin 002 had 31.4%. Bin001 has 231 scaffolds whereas Bin 002 has 164 scaffolds. The coding density of Bin001 is 69.6% and that of Bin002 is 60% and number of pseudogenes observed was also higher in Bin001 [25 pseudogenes] compared to that of Bin 002 [12 pseudogenes]. The relatively lower coding densities observed in the genomes may be due to rapid gene loss and pseudogene formation (Beam et al., 2020) causing the difference in genome sizes of both the bins. A few members of *Asgard* archaea have relatively lower genome size (0.3-0.4 Mb) and few have genome size greater than 5 Mb. This difference in the genome sizes among the members of Heimdallarchaeotes might be due to the variation in the procedure and bioinformatics analysis carried out during MAG assembly and binning. We failed to extract 16S rRNA gene from both the bins, however Bin001 showed 50 t-RNAs and Bin002 showed 22 t-RNAs. The genomes of both the bins are represented in **Figure S2**. The completeness and contamination of the genomes was checked by CheckM based on the single copy marker-gene approach and both the genomes are estimated to be >44% complete and <5% contaminated. The homology-based search of the bins with the single copy marker genes of CheckM showed that Bin001 has 63% marker completeness whereas Bin002 has only 28%. After considering the assembly statistics and bin parameters, based on the Minimum Information about Metagenome-Assembled Genome (MIMAG) criteria, the bins obtained from host *Candida tropicalis* JY101 are low quality drafts. However, these low-quality drafts also can be used to provide insights into metabolic potential of the higher organisms and helps in understanding the concepts of endosymbiosis and host-microbe interactions. The low abundance of *Asgard* group in the *C. tropicalis* holomicrobiome inferred from the taxonomic distribution data, have resulted in failure of few members of *Asgard* archaea failed to reassemble and/or bin into complete MAGs.

### Phylogenetic assessment of the binned archaeal genomes

**Figure 1:**
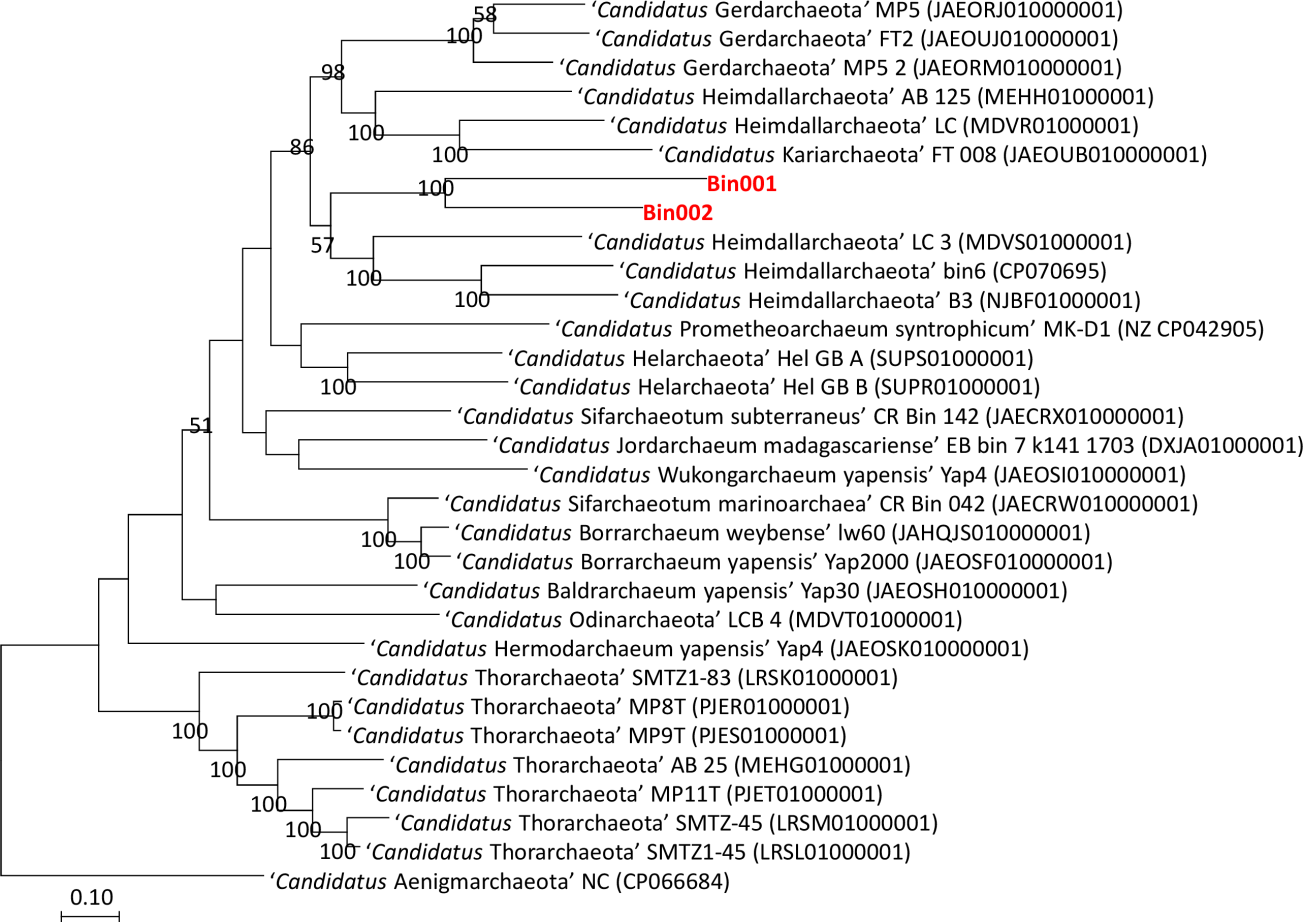
Raxml based phylogenomic tree of members belonging to phylum *Asgardarchaeota*. Genome of “*Candidatus* Aenigmarchaeota NC” (CP066684) was used as outgroup.

**Figure 2:**
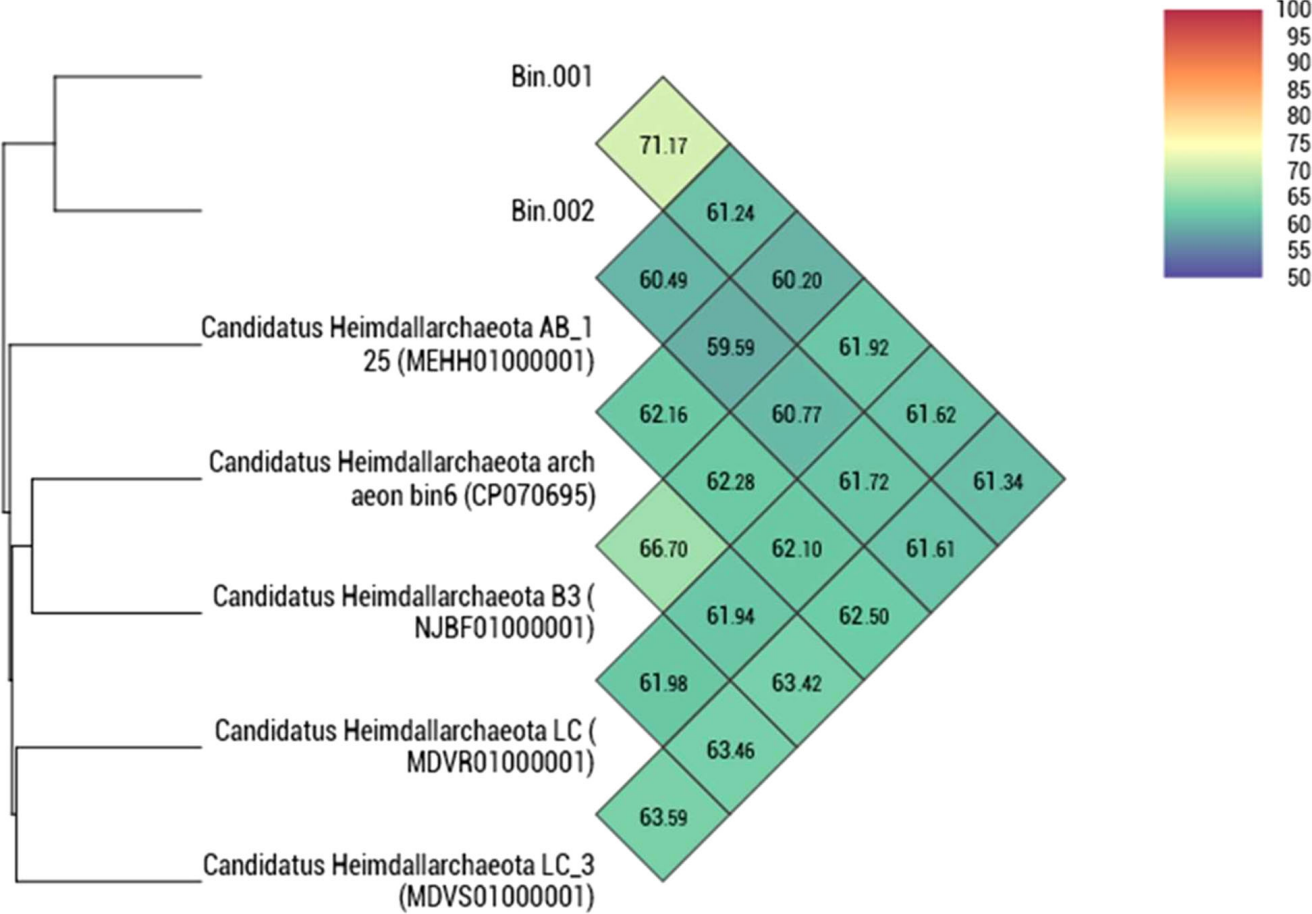
Heatmap depicting the orthoANI values among Bin001 and Bin002 along with the members of “*Candidatus* Heimdallarchaeota”.

The taxonomic position of the two binned genomes was assessed using the GTDB database (https://gtdb.ecogenomic.org/). This analysis showed that both the genomes belong to the phylum *Asgardarchaeota* with the presence of 79 archaeal marker genes out of the total 122 markers (covering 65% of archaeal marker genes; **Table S2**). The phylogenomic standing of the binned archaeal genomes was analyzed across the archaeal tree using PhyloPhlAn V 3.0 (Asnicar et al., 2020). The nearest reference archaeal organisms based on the GTDB database are the members of ‘*Ca.* Hemidallarchaeota’ (**Figure 1**, **Figure S3**). Between the two assembled genomes, the average nucleotide index (ANI) was 71.2%. With ‘*Ca.* Hemidallarchaeota’ (MEHH00000000) the ANI value of Bin001 and Bin002 were 61.2% and 60.4%, respectively (**Figure 2**).

### Metabolic annotation of the metagenome-assembled genomes

Both the binned genomes possessed carbon, energy, nucleotide, and amino acid metabolism-related genes. Further, genes related to archaeal ribosomal genes (S8, L10, L15, L13), protein biosynthesis, heat shock proteins (*GrpE*), and protein folding genes (Thermosome subunit, chaperone proteins *DnaK, DnaJ, ClpB* protein) were also found (**Tables S2–S4**).

### Carbon metabolism

Both the binned genomes have genes belonging to glycolysis/gluconeogenesis, citrate cycle, and pentose phosphate pathway. It is also further predicted that the acetyl-CoA formed from sugar/protein degradation could enter the other metabolic pathways mainly by the tricarboxylic acid pathway (TCA) or the reductive acetyl-CoA/Wood-Ljundahl (WL) pathway. The WL process is an essential pathway for archaeal methanogenesis, acetogenesis, and carbon fixation. However, most of the genes related to the Wood-Ljundahl (WL) pathway were absent except for methylenetetrahydrofolate reductase (EC 1.5.1.20), which was present in both genomes. One of the other enzymes involved in the WL pathway viz., formate-tetrahydrofolate (THF) ligase (EC 6.3.4.3) catalyzes the conversion of THF into 5, 10-methylene THF. Genes coding for this enzyme is present only in Bin 001. Both the binned genomes showed the presence of gene coding for tetrahydromethanopterin, a carrier of the C_1_ compound in archaea for carbon fixation (MacLeod et al., 2019). Both the binned genomes have genes that code for enzymes catalyzing the production of metabolites like tetrahydrofolate and tetrahydromethanopterin, which help in C_1_ metabolism, as found in *Thorarchaeota. Asgard* archaea are known to metabolize autotrophically and heterotrophically with a mixotrophic lifestyle (Liu et al., 2018; MacLeod et al., 2019).

Methanol utilization pathway (MUT) genes were also detected in the binned genomes. The functional and critical genes related to MUT like catalase (EC 1.11.1.6) were detected along with formate dehydrogenase (EC 1.17.1.9), S-(hydroxymethyl) glutathione (EC 1.1.1.284), and S-formylglutathione hydrolase (EC 3.1.2.12). Further, genes encoding ribulose biphosphate carboxylase (RuBisCo) were not found in both the genomes. In *Asgard* archaea, RuBisCo gene (the central gene in Calvin-Benson-Bassham phototrophic pathway) was shown not to be photosynthetic but assimilatory in nature (Aono et al., 2012). Therefore, the absence of the RuBisCo gene shows that these archaea are incapable of assimilating nucleotides into the central carbon metabolic pathways. Also, the gene complex of methyl coenzyme M reductase (*mcr*) was not detected in the genomes, highlighting inability for methane production (**Table S5**). These archaea showed the capability to utilize amino acids and sugars, suggesting a heterotrophic lifestyle.

### Other metabolic processes

Compared to the other members of the *Heimdallarchaeota*, nitrogen cycling genes like those for nitrate reductase and nitrite reductase were absent in both the binned genomes, thus, lacking potential for denitrification and dissimilatory nitrate reduction. In Bin 001 genome, genes related to sulfur metabolism like sulfite reductase and phosphoadenylyl-sulfate reductase (thioredoxin) show their putative role in the dissimilatory reduction of sulphur and sulfate activation. However, genes coding thiosulfate sulfur transferase were absent in both the binned genomes, suggesting that these archaea may not be central to the transformation or reduction of the intermediate sulfur molecules. However, it needs to be noted that, the absence of certain genes from these processes could be because of the draft genome resulting from the generation of the fragmented metagenomic DNA data (**Figure S3**). In the archaeal Bin 002 genome, several SNARE proteins like syntaxin 6N, vesicle (V)-SNARE, and syntaxin-2 were found and in Bin 001 SNARE protein belonging to Syntaxin 6 was predicted. The *Heimdallarchaeota*, previously referred to as the ancient archaea group (AAG) contains eukaryotic signature proteins (ESPs). The annotation also predicted the presence of hypothetical protein with small GTP-binding domain (Rab), hypothetical protein with small GTP-binding domain and PHA03378 domain (Rab), hypothetical protein with ubiquitin-conjugating enzyme-like domain (Ubiquitin-conjugating enzyme), Vps25-like protein (ESCRT-II), Vps4 ATPase and EAP30 domain protein (ESCRT-II) (Vps22/36-like) which helps in membrane remodelling. The annotation also predicted genes related to actin family, OST3 protein N-linked glycosylation etc (**Tables S3–S5**).

*Candida tropicalis* is an emerging opportunistic pathogen. The MAGs showed few virulent genes encoding for putative sodium:solute symporter, Serine/threonine protein phosphatase (EC 3.1.3.16), Glucosamine-6-phosphate deaminase (EC 3.5.99.6), DNA topoisomerase I, eukaryotic-type (EC 5.99.1.2), Chaperone protein ClpB (ATP-dependent unfoldase) and few hypothetical proteins that were found in *C. albicans* also. The bins also showed a few numbers of genes coding for resistance against antimicrobials like fusidic acid, isoniazid, triclosan, and mupirocin which might be helping the yeast to survive against these antimicrobials and the human host-associated bacterial pathogens.

## Discussion

*Candida tropicalis* is one of the most significant *Candida* species and is considered as virulent after *C. albicans*. The presence of endosymbionts may have some clinical applications i.e, a unique conversation between host and endosymbiont entities allows them to live, reproduce, and occupy distinct niches that neither of them or other living things could take over. By employing bacteria, which operate as slaves when within the yeast host and for food when removed, yeast is able to survive (Zuza-Alves et al., 2017). The endosymbiotic archaea might also contribute to the development of immune responses like pathogen elimination or immune homeostasis with some degree of selectivity in the human host and provide defence against pathogens and antibiotics, thus helping the yeast to survive in the human host (Oka et al., 2022).

To the best of our knowledge, there is no published information on the endo-archaea relationship with the yeasts. Therefore, this may be the first report of the enigmatic relationship between the two entities. However, these findings have to be corroborated by culturing them and studying their cellular mechanisms. As no single ‘*Candidatus*’ strain of archaea has been isolated from the clade of ‘*Heimdallarchaeota’*, we have to depend on the structural and functional annotations of their metagenome-assembled genomes (MAGs) or single amplified genome (SAGs) for their genetic and protein information. The *Heimdallarchaeota*, formerly grouped as the ancient archaeal group (AAG) are the nearest known relatives of eukaryotes as shown by phylogenomic studies (Takai and Horikoshi, 1999; Zaremba-Niedzwiedzka et al., 2017).

The metagenomic studies permit the exploration of microbial diversity and metagenomic binning is proven to be one of the powerful tools for the extraction of genomes belonging to rare community members (<1%). Various binning software uses criteria like nucleotide signatures, marker gene phylogenies and depth of DNA sequence coverage etc. for the construction of high-quality bins. The MAGs constructed may show data quality problems mainly due to downstream analysis involving various bioinformatics tools. These include the assembly of metagenomic sequences and binning of assembled sequences based on innate sequence properties and abundance across samples and might result in low-quality MAGs which might represent unnatural constructs, genome-like patchworks of genes resulting from assembly and/or the binning process (Sriram et al., 2021). Thus, low-quality draft genomes as obtained in this study might not be used in genetic linkage studies but may be suitable for fragment recruitment analyses, metabolic predictions and phylogenetic analysis (Bowers et al., 2017).

The low-quality draft genome completeness [classified according to Bowers et al., 2017] of the MAGs in our study could be attributed to the source of their isolation since most studies related to the endosymbionts with high-quality (85-90%) draft genomes were from samples that were enriched for endosymbionts before isolation and analysis (Lind et al., 2018; Cooper and Cressler, 2020). Nevertheless, the two draft genomes of this study indeed provide pivotal information on the existence of archaeal members in the yeast, *C. tropicalis*. Further, our study revealed that both archaeal genomes clade separately (**Figure S3**) within the ‘*Candidatus’* superphylum, designated as *Asgard* archaea. In contrast to the bacterial endosymbionts and free-living members with reduced genomes (McCutcheon and Moran, 2012), neither of the binned genomes had a reduced genome size (**Table S1**). This might be due to the gain of significant amounts of genes and most of the genes might be in the phase of undergoing pseudogenization (Lind et al., 2018).

Besides validating the Bin001 and Bin002 genomes position in the archaeal tree of life, a glimpse of the novel insights into the endo-archaeal relationship with the eukaryotic host was discussed in this study. It has revealed that the endo-archaea encode several essential genes, which are central in vesicle fusion and transport. Both the binned genomes have signature protein coding genes of eukaryote and bacteria. To date, 24 SNARE proteins were reported from the yeast members (Furukawa and Mima, 2014). Syntaxin 6 found in Bin001 genome is said to be homologous to the Tlg1p protein in yeasts involved in post-Golgi trafficking events (Gerrard et al., 2000). Therefore, it can be hypothesized that the endo-archaea of *C. tropicalis* in this study could be a probable linking and transitional genetic juncture in the origin of eukaryotes. The presence of ESPs like small GTPases, proteins helping in membrane remodelling in the bins extracted from the metagenome of hologenome of *C. tropicalis* JY101 strongly boosted eocyte hypothesis that states *Asgard* archaea as closest relative to eukaryotes (Wu et al., 2022). Overall, it is also proven that ancestral elements are all more complex than previously anticipated. However, in-depth molecular studies are required for better discernment of the relationship between the two entities.

An interesting aspect of the endo-archaeon relationship with *C. tropicalis* can also be predicted based on symbiotic and nutritional dependency. Metabolic genes involved in C_1_ fixation like those coding for tetrahydrofolate and tetrahydromethanopterin were detected in both the bins enabling them to fix carbon autotrophically and heterotrophically. Therefore, taking us to a common point that the yeast could have retained archaea for its nutritional fulfilment, as such a phenomenon is often seen in phagotrophic protists (Takashi et al., 2017). Further, methylotrophy in yeasts is often restricted to a limited number of genera like *Candida*, *Hansenula*, and *Pichia* (Yurimoto and Sakai, 2019; Rozanov et al., 2020). The important genes of the methanol utilisation (MUT) pathway were also found in both the binned genomes. This raises the question of whether the ability of the yeast to metabolize methanol is by using its own MUT genes or from those derived from the endomicrobiome?

Both the binned genomes showed that they have genes associated with DNA replication, DNA repair, transcription, and translation but lack the biosynthetic capacity to synthesize nucleotides, a few amino acids, lipids, and other cofactors. The absence of such essential biosynthetic pathways implies that they may likely have a symbiotic lifestyle (Xie et al., 2022). The binned genomes also showed the presence of partial tricarboxylic acid (TCA) cycle and capability of the archaea in having anaerobic metabolism. The binned genomes also showed genes coding for proteasome regulatory subunit Rpn2, proteasome subunit beta7 (EC 3.4.25.1), proteasome subunit beta6 (EC 3.4.25.1), proteasome subunit beta1 (EC 3.4.25.1) as well as several molecular chaperones indicating that they have the ability to degrade damaged or misfolded proteins into oligopeptides. The binned genomes also have genes coding for peptidases belonging to different families (M3, Aminopeptidase, C19, M16 and M18 family), peptide and oligopeptide transporters. This shows that oligopeptides and amino acids are likely to be important substrates for the survival of the archaea and may be obtained from the yeast cell indicating a probable endosymbiotic relationship. The archaea residing in the yeast cells may obtain diverse nutrients from hosts, and in turn, they may provide dietary carbon, particularly during C1 carbon metabolism.

Both the binned genomes revealed a variegated character of eukaryotic genomic signatures and prokaryotic functional properties. Our examination of these MAGs shows diverse nutrient acquisition and metabolic pathway genes that may benefit the host directly or indirectly. The holobionts with their hologenomes have to be further tested in terms of diversity, coevolution and functioning to uncover the biological facts of yeast-endo-microbiota interactions. There is still a gap between the understanding of the evolution of *Asgard* archaea, endosymbiotic association and the geochemical roles of these archaea. This is mainly due to the non-availability of high-quality genomes of highly diverse, evolutionarily and ecologically important *Asgard* archaea. Analysing the additional genomes and cultivation of *Asgard* archaea will provide insights and enable a greater understanding of the ecological and geochemical roles of archaea in Earth’s history and their role in endosymbiosis.

## Data availability statement

The datasets presented in this study can be found in online repositories. The names of the repository/repositories and accession number(s) can be found below: https://www.ncbi.nlm.nih.gov/, JAMCOH00000000, https://www.ncbi.nlm.nih.gov/, JAMCOI00000000.

## Author contributions

UJ, AR, BI, are involved in the analysis of data. SI contributed to the C1 metabolism. All authors contributed to the article and approved the submitted version.
