# Supplementary material for: Characterization of metagenome-assembled genomes of two endo-archaea of Candida tropicalis
Source: Front Microbiomes. 2023 Feb 1;1:1020341. doi: 10.3389/frmbi.2022.1020341 (PMC12993457; doi:10.3389/frmbi.2022.1020341)
Supplement: Supplementary file 1 [file DataSheet_1.pdf]

Characterization of Metagenome-Assembled Genomes of two endo-archaea of *Candida tropicalis*

[1]Uppada Jagadeeshwari., [1*]Chintalapati Sasikala ,[2]Rai Anusha., B. Indu[2]., Sahu Ipsita[2] and [1*]Chintalapati Venkata Ramana

[1] Bacterial Discovery Laboratory, Centre for Environment, IST, JNT University Hyderabad, Kukatpally, Hyderabad-500 085, India .

[2]Department of Plant Sciences, School of Life Sciences, University of Hyderabad, P.O. Central University, Hyderabad 500 046, India.

*Author for correspondence: Sasikala, Ch., Ramana, Ch. V.

E-mail: sasi449@yahoo.ie; sasikala.ch@gmail.com; cvr449@gmail.com

Running title: Metagenomics assisted genomes of the two endophytic archaeal genomes binned from *Candida tropicalis* JY101

Keywords: Metagenome-assisted genomes, *Heimdallarchaeota, Candida tropicalis*, *Asgardarchaeota*

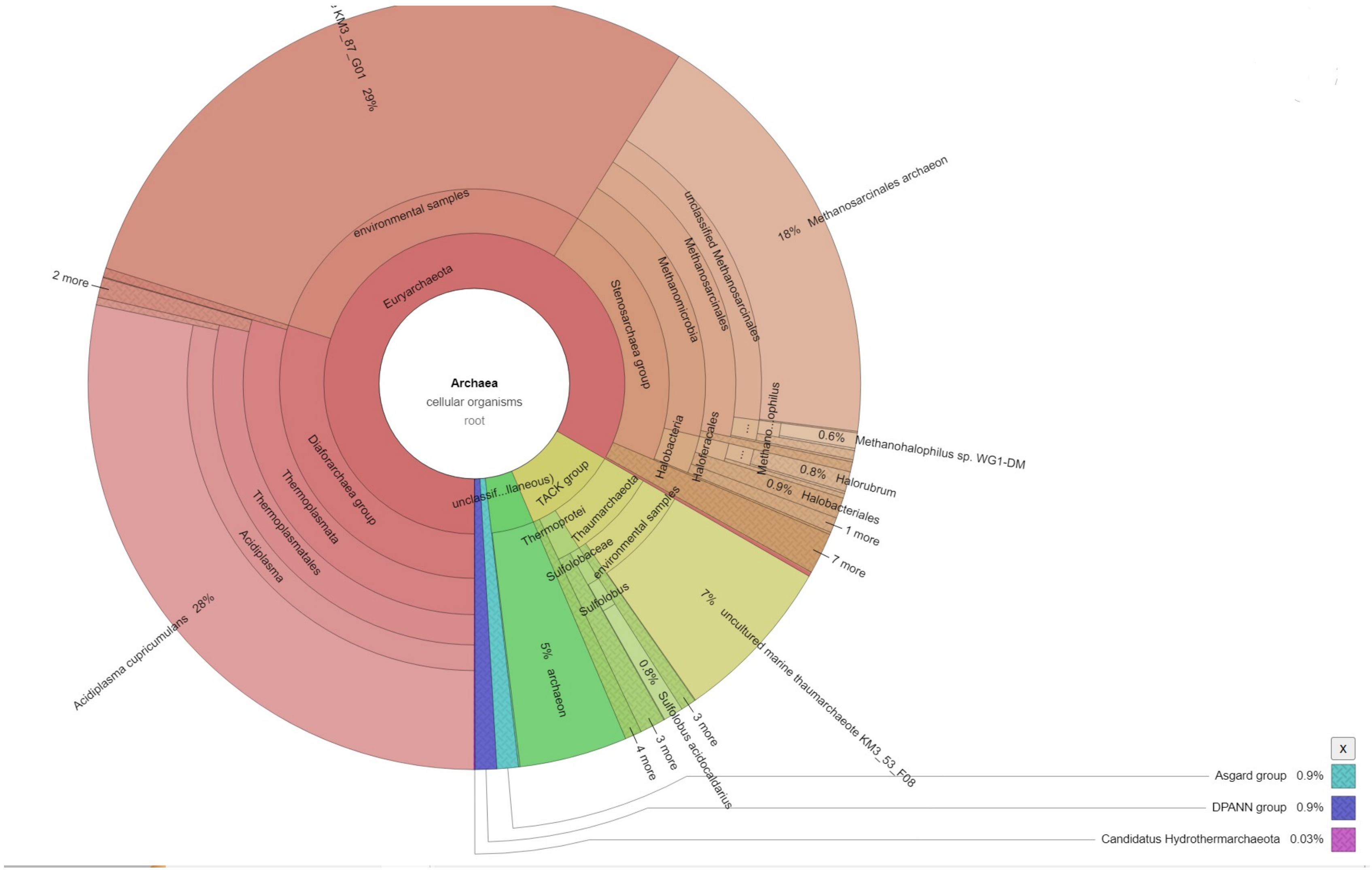

Fig S1: Krona plot depicting the overview of community structure of the archaeal microbiome associated with the host *Candida tropicalis* JY101.

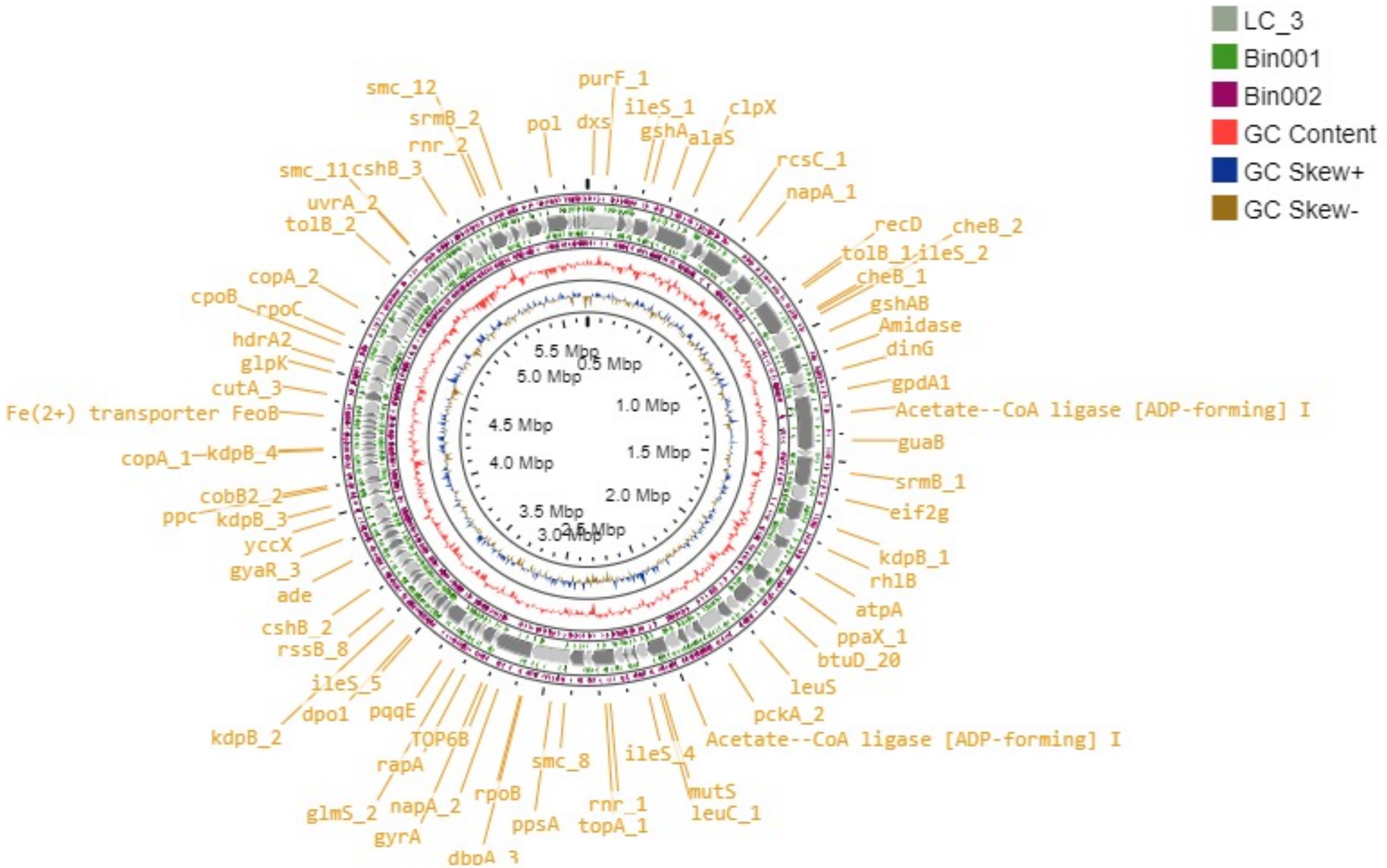

Fig. S2: Circular representation of genomes of Bin 001' and Bin 002 along with the members of ' *Candidatus* Heimdallarchaeota' LC 3.

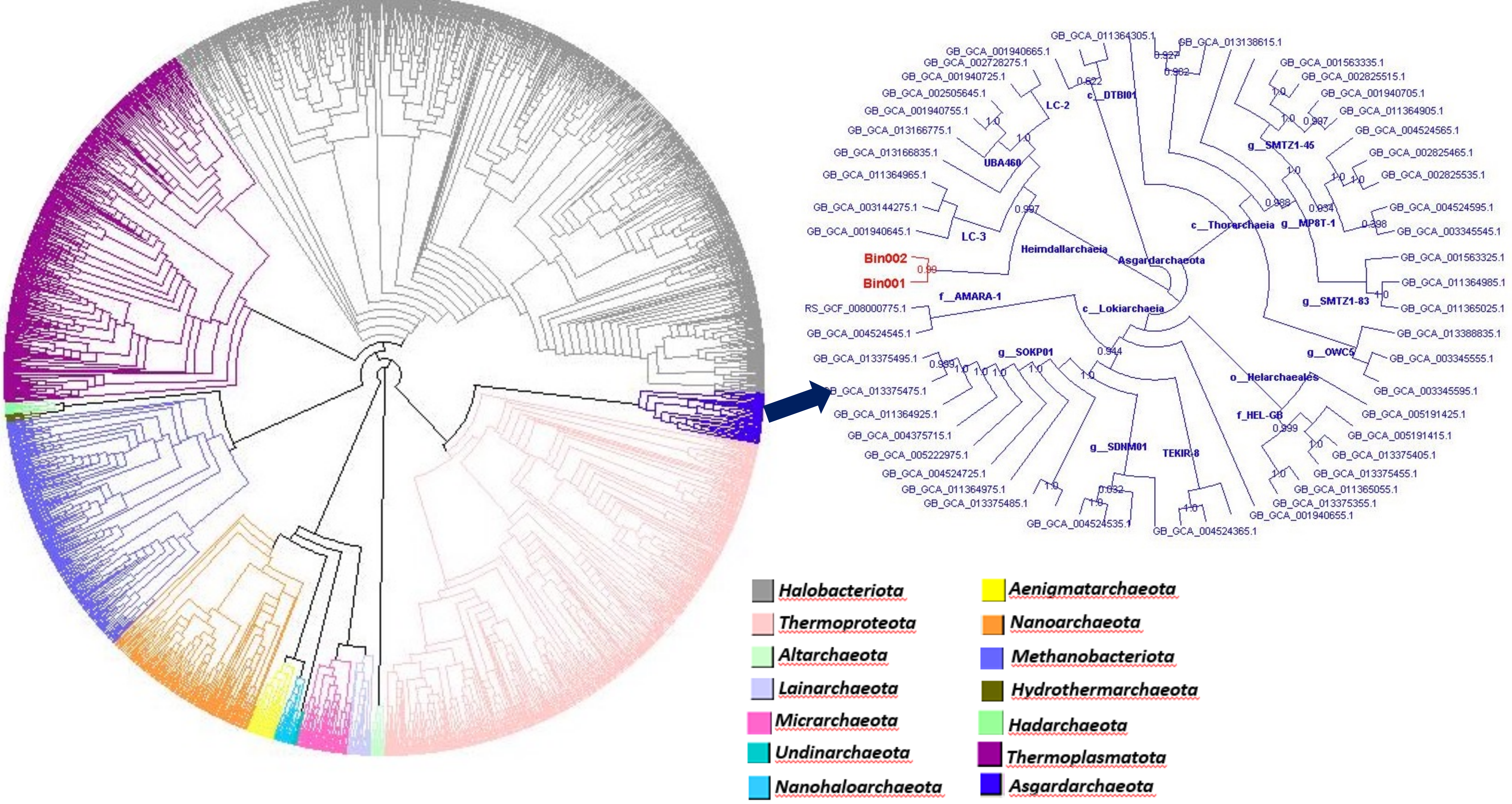

Fig. S3. Phylogenomic tree of members belonging to domain Archaea. The phylogenomic tree was constructed using phylophlan v3.0

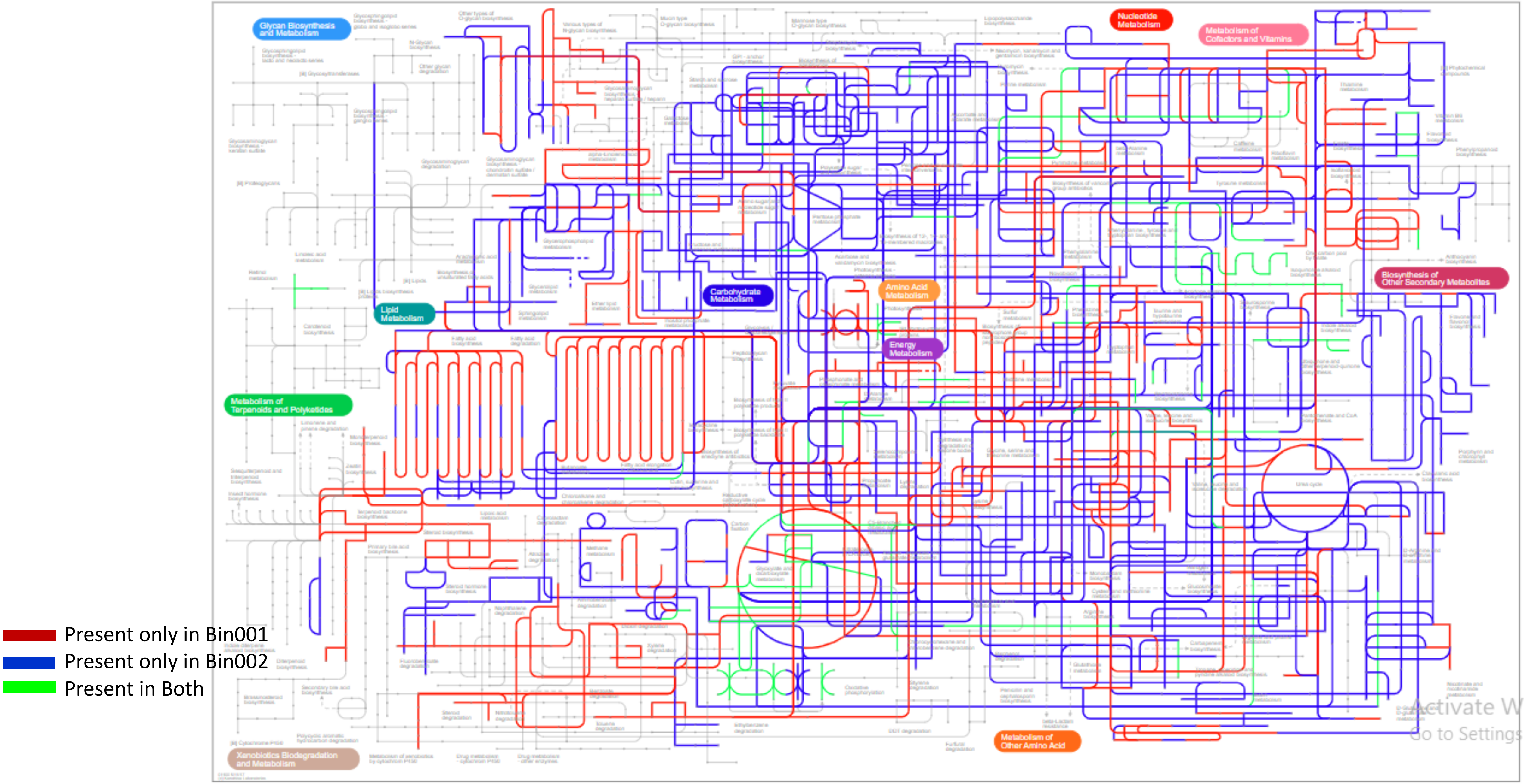

Fig S4: Comparison of metabolic reconstruction of metagenome assisted genomes (MAG's) of two endo-archaea Bin 001 and Bin 002 of *Candida tropicalis* JY101

Table S1: Comparative genomic features among the Metagenome assisted genomes (MAGs) Bin001 and Bin002 obtained from Candida tropicals JY101 and nearest neighbor ‘Ca.’ Heimdallarchaeota LC_3

Organism	Accession No	Sequencing Technology	Genome coverage	Genome size (Mb)	No of contigs	GC %	N50 (bp)	Genes (total)	CDS (Total)	CDS (Protein)	RNA	Pseudogene
Bin 001	JAMCOH010000000		50	5.38	230	34.3	23,859	2,748	2,698	2,673	50	25
Bin002	JAMCOI000000000	Illumina HiSeq	50	4.22	164	31.2	15,902	1,920	1,898	1,886	22	12
‘Ca.’ Heimdallarchaeota LC_3	MDVS00000000		136	5.68	157	29.7	59,313	5,542	5516	5514	28	2

Table S2: Description of GTDB archeal marker genes observed in metagenome assisted genomes (MAGs)

Marker Id	Name	Description	Bin001	Bin002
PF00368.19	HMG-CoA_red	Hydroxymethylglutaryl-coenzyme A reductase	+	+
PF00410.20	Ribosomal_S8	Ribosomal protein S8	+	+
PF00466.21	Ribosomal_L10	Ribosomal protein L10	-	+
PF00687.22	Ribosomal_L1	Ribosomal protein L1p/L10e family	+	+
PF00827.18	Ribosomal_L15e	Ribosomal L15	+	+
PF00900.21	Ribosomal_S4e	Ribosomal family S4e	-	+
PF01000.27	RNA_pol_A_bac	RNA polymerase Rpb3/RpoA insert domain	-	+
PF01015.19	Ribosomal_S3Ae	Ribosomal S3Ae family	+	+
PF01090.20	Ribosomal_S19e	Ribosomal protein S19e	-	+
PF01092.20	Ribosomal_S6e	Ribosomal protein S6e	+	+
PF01157.19	Ribosomal_L21e	Ribosomal protein L21e	-	+
PF01191.20	RNA_pol_Rpb5_C	RNA polymerase Rpb5, C-terminal domain	-	+
PF01194.18	RNA_pol_N	RNA polymerases N / 8 kDa subunit	+	+
PF01198.20	Ribosomal_L31e	Ribosomal protein L31e	-	+
PF01200.19	Ribosomal_S28e	Ribosomal protein S28e	-	-
PF01269.18	Fibrillarin	Fibrillarin	+	+
PF01280.21	Ribosomal_L19e	Ribosomal protein L19e	-	+
PF01282.20	Ribosomal_S24e	Ribosomal protein S24e	+	+
PF01496.20	V_ATPase_I	V-type ATPase 116kDa subunit family	+	+
PF01655.19	Ribosomal_L32e	Ribosomal protein L32	-	+
PF01798.19	-p	s-RNA binding domain, fibrillarin	+	+
PF01864.18	CarS-like	CDP-archaeol synthase	-	-
PF01866.18	Diphthamide_syn	Putative diphthamide synthesis protein	+	+
PF01868.17	UPF0086	Domain of unk-wn function UPF0086	-	+
PF01984.21	dsDNA_bind	Double-stranded DNA-binding domain	-	+
PF01990.18	ATP-synt_F	ATP synthase (F/14-kDa) subunit	-	+
PF02006.17	PPS_PS	Phosphopantothenate/pantothenate synthetase	-	-
PF02978.20	SRP_SPB	Signal peptide binding domain	+	+
PF03874.17	RNA_pol_Rpb4	RNA polymerase Rpb4	+	+
PF04019.13	DUF359	Protein of unk-wn function (DUF359)	-	-
PF04104.15	DNA_primase_lrg	Eukaryotic and archaeal DNA primase, large subunit	-	+
PF04919.13	DUF655	Protein of unk-wn function (DUF655)	-	-
PF07541.13	EIF_2_alpha	Eukaryotic translation initiation factor 2 alpha subunit	+	+
PF13656.7	RNA_pol_L_2	RNA polymerase Rpb3/Rpb11 dimerisation domain	-	+
PF13685.7	Fe-ADH_2	Iron-containing alcohol dehydrogenase	+	+

TIGR00021	rpiA	ribose 5-phosphate isomerase A	-	+
TIGR00037	eIF_5A	translation elongation factor IF5A	-	+
TIGR00042	TIGR00042	-n-ca-nical purine NTP pyrophosphatase, RdgB/HAM1 family	-	+
TIGR00064	ftsY	signal recognition particle-docking protein FtsY	-	-
TIGR00111	pelota	mRNA surveillance protein pelota	+	+
TIGR00134	gatE_arch	glutamyl-tRNA(Gln) amidotransferase, subunit E	-	-
TIGR00240	ATCase_reg	aspartate carbamoyltransferase, regulatory subunit	-	-
TIGR00264	TIGR00264	alpha-NAC homolog	-	-
TIGR00270	TIGR00270	TIGR00270 family protein	-	-
TIGR00279	uL16_euk_arch	ribosomal protein uL16	-	+
TIGR00283	arch_pth2	peptidyl-tRNA hydrolase	+	+
TIGR00291	RNA_SBDS	rRNA metabolism protein, SBDS family	-	+
TIGR00293	TIGR00293	prefoldin, alpha subunit	-	+
TIGR00307	eS8	ribosomal protein eS8	+	+
TIGR00308	TRM1	N2,N2-dimethylgua-sine tRNA methyltransferase	+	+
TIGR00323	eIF-6	putative translation initiation factor eIF-6	+	+
TIGR00324	endA	tRNA-intron lyase	+	+
TIGR00335	primase_sml	putative DNA primase, eukaryotic-type, small subunit	+	+
TIGR00336	pyrE	orotate phosphoribosyltransferase	-	+
TIGR00337	PyrG	CTP synthase	-	+
TIGR00373	TIGR00373	transcription factor E	-	-
TIGR00389	glyS_dimeric	glycine--tRNA ligase	+	+
TIGR00392	ileS	isoleucine--tRNA ligase	+	+
TIGR00398	metG	methionine--tRNA ligase	+	+
TIGR00405	KOW_elon_Spt5	transcription elongation factor Spt5	-	-
TIGR00408	proS_fam_I	proline--tRNA ligase	+	+
TIGR00422	valS	valine--tRNA ligase	+	+
TIGR00425	CBF5	putative rRNA pseudouridine synthase	-	+
TIGR00432	arcsn_tRNA_tgt	tRNA-guanine(15) transglycosylase	-	-
TIGR00442	hisS	histidine--tRNA ligase	-	+
TIGR00448	rpoE	DNA-directed RNA polymerase	-	+
TIGR00456	argS	arginine--tRNA ligase	-	+
TIGR00458	aspS_-ndisc	aspartate--tRNA(Asn) ligase	+	+
TIGR00463	gltX_arch	glutamate--tRNA ligase	-	+
TIGR00468	pheS	phenylalanine--tRNA ligase, alpha subunit	+	+
TIGR00471	pheT_arch	phenylalanine--tRNA ligase, beta subunit	-	+
TIGR00490	aEF-2	translation elongation factor aEF-2	+	+
TIGR00491	aIF-2	translation initiation factor aIF-2	-	+
TIGR00501	met_pdase_II	methionine ami-peptidase, type II	-	+

TIGR00521	coaBC_dfp	phosphopantothe-ylcysteine decarboxylase / phosphopantothenate--cysteine ligase	-	-
TIGR00522	dph5	diphthine synthase	-	-
TIGR00549	mevalon_kin	mevalonate kinase	+	+
TIGR00658	orni_carb_tr	ornithine carbamoyltransferase	-	+
TIGR00670	asp_carb_tr	aspartate carbamoyltransferase	+	+
TIGR00729	TIGR00729	ribonuclease HII	-	+
TIGR00936	ahcY	ade-sylhomocysteinase	-	+
TIGR00982	uS12_E_A	ribosomal protein uS12	-	+
TIGR01008	uS3_euk_arch	ribosomal protein uS3	-	+
TIGR01012	uS2_euk_arch	ribosomal protein uS2	+	+
TIGR01018	uS4_arch	ribosomal protein uS4	-	+
TIGR01020	uS5_euk_arch	ribosomal protein uS5	-	+
TIGR01025	uS19_arch	ribosomal protein uS19	+	+
TIGR01028	uS7_euk_arch	ribosomal protein uS7	-	+
TIGR01038	uL22_arch_euk	ribosomal protein uL22	-	+
TIGR01046	uS10_euk_arch	ribosomal protein uS10	+	+
TIGR01052	top6b	DNA topoisomerase VI, B subunit	-	-
TIGR01060	e-	phosphopyruvate hydratase	-	+
TIGR01077	L13_A_E	ribosomal protein uL13	+	+
TIGR01080	rplX_A_E	ribosomal protein uL24	-	+
TIGR01213	pseudo_Pus10arc	tRNA pseudouridine(54/55) synthase	-	-
TIGR01309	uL30_arch	ribosomal protein uL30	-	-
TIGR01952	nusA_arch	NusA family KH domain protein, archaeal	-	-
TIGR02076	pyrH_arch	putative uridylate kinase	-	-
TIGR02153	gatD_arch	glutamyl-tRNA(Gln) amidotransferase, subunit D	-	-
TIGR02236	recomb_radA	DNA repair and recombination protein RadA	-	-
TIGR02258	2_5_ligase	2'-5' RNA ligase	-	-
TIGR02338	gimC_beta	prefoldin, beta subunit	-	-
TIGR02389	RNA_pol_rpoA2	DNA-directed RNA polymerase, subunit A''	-	-
TIGR02390	RNA_pol_rpoA1	DNA-directed RNA polymerase subunit A'	-	-
TIGR02651	RNase_Z	ribonuclease Z	+	+
TIGR03626	L3_arch	ribosomal protein uL3	-	-
TIGR03627	uS9_arch	ribosomal protein uS9	-	-
TIGR03628	arch_S11P	ribosomal protein uS11	-	-
TIGR03629	uS13_arch	ribosomal protein uS13	-	-
TIGR03636	uL23_arch	ribosomal protein uL23	-	-
TIGR03653	uL6_arch	ribosomal protein uL6	-	-
TIGR03665	arCOG04150	arCOG04150 universal archaeal KH domain protein	-	-
TIGR03670	rpoB_arch	DNA-directed RNA polymerase subunit B	-	-
TIGR03671	cca_archaeal	CCA-adding enzyme	-	-
TIGR03672	rpl4p_arch	50S ribosomal protein uL4	-	-

TIGR03673	uL14_arch	50S ribosomal protein uL14	-	-
TIGR03674	fen_arch	flap structure-specific endonuclease	-	-
TIGR03677	eL8_ribo	ribosomal protein eL8	-	-
TIGR03680	eif2g_arch	translation initiation factor 2, gamma subunit	-	-
TIGR03683	A-tRNA_syn_arch	alanine--tRNA ligase	-	-
TIGR03684	arCOG00985	arCOG04150 universal archaeal PUA-domain protein	-	-
TIGR03722	arch_KAE1	universal archaeal protein Kae1	-	-

Table S3: Annotation data for proteins obtained from Bin001 obtained from shotgun metagenome sequence of *Candida tropicalis* JY101. The locus tag, predicted product, KEGG/COG/CAZY annotation are provided (if available)

Locus Tag	Product	COG	KEGG	CAZY
JAMCOH010000001.1	ATP-binding RNA helicase involved	COG0513	K13181	-
JAMCOH010000001.1	hypothetical protein		K20318	-
JAMCOH010000001.1	hypothetical protein	COG2072	-	-
JAMCOH010000001.1	Adenylate cyclase	COG2114	K01768	-
JAMCOH010000001.1	hypothetical protein		K19880	-
JAMCOH010000001.1	hypothetical protein	COG5198	K10703	-
JAMCOH010000001.1	hypothetical protein	COG5198	K10703	-
JAMCOH010000001.1	Glutamine amidotransferases class-	COG0449	K18802	-
JAMCOH010000001.1	SIT4 phosphatase-associated protein		K15457,K15458,K15501	-
JAMCOH010000001.1	Serine/Threonine protein kinases, catalytic domain		K11229	-
JAMCOH010000001.1	Mannosyltransferase		K03847	GT22
JAMCOH010000001.1	Metal chaperone, involved in Zn ho	COG0523	-	-
JAMCOH010000001.1	hypothetical protein		K17792	-
JAMCOH010000001.1	Dihydrofolate reductase (EC 1.5.1.3	COG0262	K00287	-
JAMCOH010000001.1	hypothetical protein		K18646,K18647	-
JAMCOH010000001.1	hypothetical protein		K11275	-
JAMCOH010000001.1	Pyridoxamine 5'-phosphate oxidase	COG5135	K00275	-
JAMCOH010000002.1	hypothetical protein		K10863	-
JAMCOH010000002.1	hypothetical protein	COG5175	K10643	-
JAMCOH010000002.1	Isoleucyl-tRNA synthetase (EC 6.1.1	COG0060	K01870	-
JAMCOH010000002.1	hypothetical protein		K10885	-
JAMCOH010000002.1	hypothetical protein	COG1161	K19828	-
JAMCOH010000002.1	hypothetical protein	COG4088	K15456	-
JAMCOH010000002.1	hypothetical protein	COG5223	K14769	-
JAMCOH010000002.1	hypothetical protein		K10729	-
JAMCOH010000002.1	hypothetical protein	COG5084	-	-
JAMCOH010000002.1	Protein tyrosine kinase		K12765	-
JAMCOH010000002.1	hypothetical protein	COG0604	-	-
JAMCOH010000002.1	Uncharacterized MFS-type transpor	COG0477	-	-
JAMCOH010000002.1	Uncharacterized MFS-type transpor	COG0477	-	-
JAMCOH010000002.1	hypothetical protein	COG3332	-	-
JAMCOH010000002.1	Uncharacterized NRDE family prote	COG3332	-	-
JAMCOH010000002.1	Chaperone protein DnaJ		K09506	-
JAMCOH010000002.1	hypothetical protein		K08342	-
JAMCOH010000003.1	hypothetical protein		K12612	-
JAMCOH010000003.1	hypothetical protein		K11361	-
JAMCOH010000003.1	hypothetical protein		K07204	-
JAMCOH010000003.1	hypothetical protein		K15119	-
JAMCOH010000003.1	hypothetical protein		K17786	-
JAMCOH010000003.1	hypothetical protein	COG5459	-	-
JAMCOH010000003.1	SSU ribosomal protein S27e	COG2051	K02978	-
JAMCOH010000003.1	hypothetical protein	COG0201	K10956	-
JAMCOH010000003.1	6,7-dimethyl-8-ribityllumazine synt	COG0054	K00794	-
JAMCOH010000003.1	Monocarboxylate transporter		-	-
JAMCOH010000003.1	Transcription initiation factor IIB	COG1405	K03124	-
JAMCOH010000003.1	2-polyprenylphenol hydroxylase	COG0654	K06126	-
JAMCOH010000003.1	Putative serine esterase	COG1075	K01046	-
JAMCOH010000003.1	Aldehyde dehydrogenase	COG1012	-	-
JAMCOH010000004.1	hypothetical protein	COG1457	-	-
JAMCOH010000004.1	nitrosoguanidine resistance protein		K07101	-
JAMCOH010000004.1	Amino acid permease	COG0531	K16261	-
JAMCOH010000004.1	Choline/Carnitine o-acyltransferase		K00624	-
JAMCOH010000004.1	hypothetical protein cation transpo	COG0474	K01530	-
JAMCOH010000004.1	hypothetical protein	COG1236	K14402	-
JAMCOH010000004.1	NADH:ubiquinone oxidoreductase		K03938	-
JAMCOH010000004.1	hypothetical protein		K12869	-

JAMCOH010000004.1	Ubiquitin-2 like Rad60 SUMO-like	COG5272	K12158	-
JAMCOH010000004.1	eRF1 methyltransferase catalytic su	COG2890	K19589	-
JAMCOH010000004.1	hypothetical protein	COG5176	K13095	-
JAMCOH010000004.1	Nitrogen permease regulator 2		K20405	-
JAMCOH010000004.1	hypothetical protein	COG5262	K11251	-
JAMCOH010000004.1	Inosine-uridine preferring nucleosid	COG1957	-	-
JAMCOH010000004.1	hypothetical protein	COG1308	K03626	-
JAMCOH010000004.1	hypothetical protein	COG5243	-	-
JAMCOH010000004.1	hypothetical protein	COG0652	K01802	-
JAMCOH010000004.1	Squalene/phytoene synthase	COG1562	K18163	-
JAMCOH010000005.1	Oligo-1,6-glucosidase (EC 3.2.1.10)	COG0366	K01182,K01187	GH13,GH31
JAMCOH010000005.1	hypothetical protein	COG1204	K18664	-
JAMCOH010000005.1	hypothetical protein		K08141	-
JAMCOH010000005.1	Oligo-1,6-glucosidase (EC 3.2.1.10)	COG0366	K01182,K01187	GH13,GH31
JAMCOH010000005.1	Ribosome biogenesis ATP-depende	COG0513	K13179	-
JAMCOH010000005.1	DNA polymerase alpha subunit B	COG1311	K02328	-
JAMCOH010000005.1	hypothetical protein	COG0557	K18748	-
JAMCOH010000005.1	GTP cyclohydrolase I (EC 3.5.4.16) t	COG0302	K01495	-
JAMCOH010000005.1	Aspartyl-tRNA(Asn) amidotransfera	COG0154	K02433	-
JAMCOH010000005.1	Methionyl-tRNA synthetase (EC 6.1	COG0143	K01874	-
JAMCOH010000005.1	Phosphatidylserine decarboxylase (	COG0688	K01613	-
JAMCOH010000005.1	Signal recognition particle receptor	COG0552	K13431,K21989	-
JAMCOH010000005.1	hypothetical protein	COG5080	K20363	-
JAMCOH010000006.1	hypothetical protein	COG0814	K14997	-
JAMCOH010000006.1	hypothetical protein		K11236	-
JAMCOH010000006.1	Electron transfer flavoprotein-ubiq	COG2440	K00311	-
JAMCOH010000006.1	Oligopeptide ABC transporter, periplasmic oligopeptic		-	-
JAMCOH010000006.1	hypothetical protein	COG5102	-	-
JAMCOH010000006.1	hypothetical protein		K20792	-
JAMCOH010000006.1	Heat Shock Protein	COG0457	K09553	-
JAMCOH010000006.1	hypothetical protein		K09043	-
JAMCOH010000006.1	hypothetical protein	COG0531	K16261	-
JAMCOH010000007.1	hypothetical protein		K22494	-
JAMCOH010000007.1	hypothetical protein	COG0557	K19782	-
JAMCOH010000007.1	hypothetical protein		K14709	-
JAMCOH010000007.1	hypothetical protein		K14709	-
JAMCOH010000007.1	hypothetical protein	COG5034	K11379,K19197,K19198	-
JAMCOH010000007.1	asparagine synthetase	COG0367	-	-
JAMCOH010000007.1	hypothetical protein Thioesterase superfamily		K00860	-
JAMCOH010000007.1	hypothetical protein	COG5021	K10591	-
JAMCOH010000007.1	25S rRNA (adenine(645)-N(1))-methyltransferase (EC :		K14850	-
JAMCOH010000007.1	hypothetical protein		K11117	-
JAMCOH010000007.1	hypothetical protein	COG2351	K07127	-
JAMCOH010000007.1	DNA glycosylase	COG0122	K01247	-
JAMCOH010000007.1	Serine aminopeptidase, S33		-	-
JAMCOH010000007.1	hypothetical protein		K03507	-
JAMCOH010000008.1	hypothetical protein		K11682	-
JAMCOH010000008.1	hypothetical protein		K17774	-
JAMCOH010000008.1	Imidazoleglycerol-phosphate dehyd	COG0131	K01693	-
JAMCOH010000008.1	23S rRNA (guanosine-2'-O-) -m	COG0566	K03218,K15507	-
JAMCOH010000008.1	Putative serine esterase		-	-
JAMCOH010000008.1	hypothetical protein	COG2930	K20523	-
JAMCOH010000008.1	Transcription initiation factor IIIA		K09191	-
JAMCOH010000008.1	hypothetical protein		K12879	-
JAMCOH010000008.1	Phospholipase B		K13333	-
JAMCOH010000008.1	hypothetical protein		K15562	-
JAMCOH010000008.1	hypothetical protein		K08150	-
JAMCOH010000008.1	Ubiquinol-cytochrome C reductase,	COG2857	K00413	-
JAMCOH010000008.1	hypothetical protein	COG5069	-	-

JAMCOH010000008.1	Serine/threonine protein kinase PrkC, regulator of sta		K03097	-
JAMCOH010000008.1	LSU ribosomal protein L15p (L27Ae	COG0200	K02876	-
JAMCOH010000009.1	N-acetylglucosaminyl transferase component (Gpi1)		K03860	-
JAMCOH010000009.1	hypothetical protein		K18182	-
JAMCOH010000009.1	SSU ribosomal protein SAe (S2p)	COG0052	K02998	-
JAMCOH010000009.1	hypothetical protein	COG4282	K00706	GT48
JAMCOH010000009.1	glucosyltransferase		K03850	GT59
JAMCOH010000009.1	hypothetical protein	COG2319	K12782	-
JAMCOH010000009.1	hypothetical protein	COG2319	K12782	-
JAMCOH010000009.1	hypothetical protein		K20522	-
JAMCOH010000009.1	hypothetical protein		K12462	-
JAMCOH010000009.1	ATP-dependent DNA helicase UvrD,	COG0210	K03657	-
JAMCOH010000009.1	hypothetical protein	COG0330	K17081	-
JAMCOH010000009.1	hypothetical protein (Ubiquitin elor	COG5113	K10597	-
JAMCOH010000009.1	LSU ribosomal protein L35e (L29p)	COG0255	K02918	-
JAMCOH010000009.1	Belongs to the small GTPase superfamily. Arf family	COG1100	K07937,K07977	-
JAMCOH010000009.1	hypothetical protein	COG0020	K19177	-
JAMCOH010000009.1	Belongs to the major facilitator superfamily. Sugar transporter (TC 2.A.1.1) family		K08139	-
JAMCOH010000009.1	hypothetical protein	COG1131	K08711,K08712	-
JAMCOH010000010.1	Dihydroorotase (EC 3.5.2.3)	COG0418	K01465	-
JAMCOH010000010.1	Thioredoxin	COG0526	K03671	-
JAMCOH010000010.1	hypothetical protein		K12859	-
JAMCOH010000010.1	Phosphatidylethanolamine-binding	COG1881	K06910	-
JAMCOH010000010.1	hypothetical protein	COG5208	K08066	-
JAMCOH010000010.1	Lysine methyltransferase	COG2940	K07117,K11426	-
JAMCOH010000010.1	hypothetical protein	COG2802	K16275	-
JAMCOH010000010.1	hypothetical protein	COG1958	K20824	-
JAMCOH010000010.1	Ribosomal protein L19	COG0335	-	-
JAMCOH010000010.1	hypothetical protein	COG0500	K19306	-
JAMCOH010000010.1	hypothetical protein		K14785	-
JAMCOH010000010.1	hypothetical protein	COG5221	-	-
JAMCOH010000010.1	Heme O synthase, protoheme IX fa	COG0109	K02257	-
JAMCOH010000010.1	proteasome regulatory subunit Rpn	COG1310	K03038	-
JAMCOH010000010.1	GPN-loop GTPase 2	COG1100	K06883	-
JAMCOH010000010.1	hypothetical protein		K17560	-
JAMCOH010000010.1	hypothetical protein		K17560	-
JAMCOH010000010.1	Acetyl-CoA hydrolase/transferase fa	COG0427	K01067	-
JAMCOH010000011.1	Fumarate hydratase class II (EC 4.2.	COG0114	K01679	-
JAMCOH010000011.1	hypothetical protein	COG5535	K10838	-
JAMCOH010000011.1	hypothetical protein		K05287	-
JAMCOH010000011.1	Serine hydrolase (FSH1)		K00287	-
JAMCOH010000011.1	mRNA (guanine-N(7)-)-methyltransferase (EC 2.1.1.56		K00565	-
JAMCOH010000011.1	hypothetical protein		K21455	-
JAMCOH010000011.1	hypothetical protein		K20286	-
JAMCOH010000011.1	hypothetical protein		K14551	-
JAMCOH010000011.1	epoxide hydrolase	COG0596	K01253	-
JAMCOH010000011.1	hypothetical protein	COG5244	-	-
JAMCOH010000011.1	hypothetical protein		K18715	-
JAMCOH010000011.1	Protein translocase subunit SecY	COG0201	K10956	-
JAMCOH010000012.1	hypothetical protein	COG5273	K18932	-
JAMCOH010000012.1	proteasome subunit alpha7 (EC 3.4	COG0638	K02727	-
JAMCOH010000012.1	hypothetical protein	COG5354	K03253	-
JAMCOH010000012.1	hypothetical protein	COG0271	K22075	-
JAMCOH010000012.1	hypothetical protein		K09561	-
JAMCOH010000012.1	Chitin synthase (EC 2.4.1.16)	COG1215	K00698	GT2
JAMCOH010000012.1	hypothetical protein	COG5184	K11493	-
JAMCOH010000012.1	DNA polymerase delta catalytic sub	COG0417	K02327	-
JAMCOH010000012.1	D-serine dehydratase (EC 4.3.1.18)	COG3616	K20498	-

JAMCOH010000012.1	Two-component system sensor hist	COG0642	K19691	-
JAMCOH010000012.1	hypothetical protein		K17768	-
JAMCOH010000012.1	FKBP-type peptidyl-prolyl cis-trans i	COG0545	K09568	-
JAMCOH010000012.1	hypothetical protein	COG5133	-	-
JAMCOH010000013.1	4-alpha-glucanotransferase (amylor	COG3408	K01196	GH13
JAMCOH010000013.1	Methylmalonate-semialdehyde dehydrogenase (EC 1.		K00140	-
JAMCOH010000013.1	Protein tyrosine kinase		K21157,K21158	-
JAMCOH010000013.1	Serine/threonine protein kinase		K08269	-
JAMCOH010000013.1	Translation elongation factor 1 beta	COG2092	K03232	-
JAMCOH010000013.1	hypothetical protein		K15204	-
JAMCOH010000013.1	hypothetical protein		K11135,K14457	-
JAMCOH010000014.1	hypothetical protein	COG0531	K09885	-
JAMCOH010000014.1	hypothetical protein	COG0531	K09885	-
JAMCOH010000014.1	hypothetical protein	COG5210	K02179	-
JAMCOH010000014.1	hypothetical protein		K12837	-
JAMCOH010000014.1	hypothetical protein	COG0443	K09486	-
JAMCOH010000014.1	Xanthine-guanine phosphoribosyltr	COG2236	K07101,K19836	-
JAMCOH010000014.1	Ubiquitin fusion degradation protei	COG5140	K14016	-
JAMCOH010000014.1	hypothetical protein	COG4581	K20223	-
JAMCOH010000014.1	hypothetical protein		K14545	-
JAMCOH010000014.1	D-Lactate dehydrogenase, cytochro	COG0277	K00102	-
JAMCOH010000014.1	Aldehyde dehydrogenase (EC 1.2.1.	COG1012	K00128,K00129	-
JAMCOH010000014.1	hypothetical protein		K15219	-
JAMCOH010000014.1	5-oxoprolinase (EC 3.5.2.9), HyuA-li	COG0145	K01469	-
JAMCOH010000014.1	Amino-transferase class IV	COG0115	K02619	-
JAMCOH010000014.1	Phosphoserine phosphatase (EC 3.1	COG0560	K01079	-
JAMCOH010000015.1	hypothetical protein		K13682	GT91
JAMCOH010000015.1	NADH dehydrogenase (EC 1.6.99.3)	COG1252	K17871	-
JAMCOH010000015.1	hypothetical protein	COG1131	K08711,K08712	-
JAMCOH010000015.1	hypothetical protein belonging AB(	COG1132	-	-
JAMCOH010000015.1	hypothetical protein	COG0531	-	-
JAMCOH010000015.1	Coproporphyrinogen III oxidase, ae	COG0408	K00228	-
JAMCOH010000015.1	hypothetical protein	COG0531	-	-
JAMCOH010000015.1	hypothetical protein	COG0596	-	-
JAMCOH010000016.1	hypothetical protein		K17263	-
JAMCOH010000016.1	haloacid dehalogenase-like hydrolase		-	-
JAMCOH010000016.1	hypothetical protein	COG0457	-	-
JAMCOH010000016.1	hypothetical protein		K19829	-
JAMCOH010000016.1	hypothetical protein	COG0051	K01867,K02946	-
JAMCOH010000016.1	tRNA (cytidine(32)/guanosine(34)-2	COG0293	K14864	-
JAMCOH010000016.1	Lysyl-tRNA synthetase (class II) (EC	COG1190	K04567	-
JAMCOH010000016.1	hypothetical protein		K03247	-
JAMCOH010000016.1	contains Pfam profile PF00300: phosphoglycerate mu		-	-
JAMCOH010000016.1	Triosephosphate isomerase (EC 5.3	COG0149	K01803	-
JAMCOH010000016.1	hypothetical protein	COG0084	-	-
JAMCOH010000016.1	hypothetical protein	COG5190	K15732	-
JAMCOH010000016.1	hypothetical protein		K10599	-
JAMCOH010000016.1	Phosphomannomutase (EC 5.4.2.8)	COG1109	K01835,K15779	-
JAMCOH010000016.1	Vanadate resistance protein		K05530	GT62
JAMCOH010000016.1	hypothetical protein	COG5080	-	-
JAMCOH010000016.1	oxidoreductase of aldo/keto reduct	COG0656	K18097	-
JAMCOH010000016.1	hypothetical protein	COG5272	K08770	-
JAMCOH010000016.1	hypothetical protein		K11886	-
JAMCOH010000016.1	Translation initiation factor 2B delta	COG1184	K03680	-
JAMCOH010000016.1	hypothetical protein	COG0474	K14802	-
JAMCOH010000016.1	Gamma-glutamyl phosphate reduct	COG0014	K00147	-
JAMCOH010000016.1	hypothetical protein	COG1648	K02304	-
JAMCOH010000016.1	hypothetical protein		K13682	GT91
JAMCOH010000016.1	hypothetical protein		K13682	GT91
JAMCOH010000016.1	hypothetical protein		K13682	GT91

JAMCOH010000016.1	hypothetical protein		K03107	-
JAMCOH010000016.1	UDP-galactose transporter	COG0697	K15275	-
JAMCOH010000016.1	hypothetical protein	COG5273	K20028	-
JAMCOH010000016.1	Deacetylases, including yeast histor	COG0123	K06067	-
JAMCOH010000016.1	NAD kinase (EC 2.7.1.23)	COG0061	K19386	-
JAMCOH010000016.1	hypothetical protein	COG0457	K06665	-
JAMCOH010000016.1	ADP-ribose pyrophosphatase (EC 3.	COG0494	K01515	-
JAMCOH010000016.1	hypothetical protein		K11368	-
JAMCOH010000016.1	NADH-ubiquinone oxidoreductase c	COG0377	K03940	-
JAMCOH010000016.1	hypothetical protein	COG2036	K11253	-
JAMCOH010000017.1	hypothetical protein		K09448	-
JAMCOH010000017.1	Serine/Threonine protein kinases		K12771	-
JAMCOH010000017.1	Putative GTP-ase activating protein	COG5347	K12488	-
JAMCOH010000017.1	hypothetical protein with small GTP-binding domain (		K07874	-
JAMCOH010000017.1	proteasome regulatory subunit Rpn3		K03033,K03126	-
JAMCOH010000017.1	hypothetical protein		K15134	-
JAMCOH010000017.1	Oligosaccharyltransferase subunit Ribophorin II		K12667	-
JAMCOH010000017.1	Signal peptidase I (EC 3.4.21.89)	COG0681	K09647	-
JAMCOH010000017.1	SSU ribosomal protein S15Ae (S8p)	COG0096	K02957	-
JAMCOH010000017.1	SSU ribosomal protein S15Ae (S8p)	COG0096	K02957	-
JAMCOH010000017.1	hypothetical protein		K20403	-
JAMCOH010000017.1	DNA polymerase epsilon subunit C	COG5208	K03506	-
JAMCOH010000017.1	LSU ribosomal protein L41mt, mitochondrial		K17422	-
JAMCOH010000017.1	FAD dependent oxidoreductase	COG0665	K00272,K00273	-
JAMCOH010000017.1	hypothetical protein	COG5498	K01180	-
JAMCOH010000017.1	hypothetical protein	COG5233	-	-
JAMCOH010000018.1	hypothetical protein		K06867	-
JAMCOH010000018.1	hypothetical protein	COG5543	-	-
JAMCOH010000018.1	hypothetical protein		K12872	-
JAMCOH010000018.1	hypothetical protein		K07441	GT1
JAMCOH010000018.1	hypothetical protein	COG1236	-	-
JAMCOH010000018.1	DNA-directed RNA polymerase III 1	COG1594	K03019	-
JAMCOH010000018.1	hypothetical protein	COG5575	K02604,K06199	-
JAMCOH010000018.1	Lipopolysaccharide kinase (Kdo/WaaP) family		K08286,K08853	-
JAMCOH010000018.1	Translation initiation factor 1A	COG0361	K03236	-
JAMCOH010000018.1	hypothetical protein		K15565	-
JAMCOH010000018.1	Alpha,alpha-trehalose-phosphate sy	COG1877	K00697,K16055,K22337	GT20
JAMCOH010000018.1	hypothetical protein		K14649	-
JAMCOH010000018.1	Heat shock chaperonin-binding mo	COG5272	K04523	-
JAMCOH010000018.1	hypothetical protein		K18658	-
JAMCOH010000019.1	Midasin, associated with 60S pre-ril	COG5271	K14572	-
JAMCOH010000019.1	Protein phosphatase 2C	COG0631	K17508	-
JAMCOH010000019.1	hypothetical protein	COG1752	K14675	-
JAMCOH010000019.1	Oligopeptide ABC transporter, periplasmic oligopeptid		K11377	-
JAMCOH010000019.1	CCA tRNA nucleotidyltransferase (E	COG0617	K00974	-
JAMCOH010000019.1	3-dehydroquinate synthase (EC 4.2.	COG0169	K13830	-
JAMCOH010000020.1	O-acetylhomoserine sulfhydrylase (	COG0626	K17069	-
JAMCOH010000020.1	Ornithine aminotransferase (EC 2.6	COG4992	K00819	-
JAMCOH010000020.1	Peptidase, M48 family	COG0501	K06013	-
JAMCOH010000020.1	hypothetical protein	COG4638	-	-
JAMCOH010000020.1	hypothetical protein		K11771	-
JAMCOH010000020.1	hypothetical protein		K05531	GT34
JAMCOH010000020.1	hypothetical protein	COG5574	K13346	-
JAMCOH010000020.1	hypothetical protein		K01549	-
JAMCOH010000020.1	hypothetical protein	COG0631	K17508	-
JAMCOH010000020.1	E3 ubiquitin-protein ligase listerin (	COG5219	K00507,K22377	-
JAMCOH010000020.1	Ammonium transporter	COG0004	K03320	-
JAMCOH010000020.1	hypothetical protein		K20042	-
JAMCOH010000020.1	hypothetical protein	COG0598	K16073	-

JAMCOH010000020.1	Aldo/keto reductase family	COG0667	K00062	-
JAMCOH010000020.1	hypothetical protein		K20121	-
JAMCOH010000021.1	hypothetical protein	COG0666	K06647	-
JAMCOH010000021.1	hypothetical protein	COG2036	K11253,K11495	-
JAMCOH010000021.1	Belongs to the actin family	COG5277	K09252,K11768	-
JAMCOH010000021.1	hypothetical protein	COG2116	K21767	-
JAMCOH010000021.1	hypothetical protein	COG1841	K02907	-
JAMCOH010000021.1	hypothetical protein		K18174	-
JAMCOH010000021.1	SAM-dependent methyltransferase	COG0500	K18162	-
JAMCOH010000021.1	ATP-dependent DNA ligase I (EC 6.5	COG1793	K10747	-
JAMCOH010000021.1	Glycerol-3-phosphate O-acyltransfe	COG0204	K13507	-
JAMCOH010000022.1	DNA repair and recombination prot	COG0553	K10841	-
JAMCOH010000022.1	hypothetical protein		K11270	-
JAMCOH010000022.1	hypothetical protein	COG1073	-	-
JAMCOH010000022.1	EAP30 domain protein (ESCRT-II) (Vps22/36-like)		K12188	-
JAMCOH010000022.1	tRNA pseudouridine(38/39) synthas	COG0101	K01855	-
JAMCOH010000022.1	ATP-dependent rRNA helicase SPB4	COG0513	K14809	-
JAMCOH010000022.1	hypothetical protein	COG0476	K04532	-
JAMCOH010000022.1	Translation elongation factor 3	COG0488	K03235	-
JAMCOH010000022.1	lysophospholipase		K13357	-
JAMCOH010000022.1	hypothetical protein	COG0695	K03676	-
JAMCOH010000022.1	SSU ribosomal protein S11e (S17p)	COG0186	K02949	-
JAMCOH010000023.1	hypothetical protein	COG5197	K20362	-
JAMCOH010000023.1	Malonyl CoA-acyl carrier protein tra	COG0331	K00645	-
JAMCOH010000023.1	hypothetical protein	COG5648	K22483	-
JAMCOH010000023.1	hypothetical protein		K14856	-
JAMCOH010000023.1	hypothetical protein	COG1405	K15196	-
JAMCOH010000023.1	Dead-Box Protein 8, ATP-dependen	COG0513	K14778	-
JAMCOH010000023.1	GTP-binding protein Obg	COG0536	K03979,K10277	-
JAMCOH010000023.1	NAD(P)H dehydrogenase (quinone)	COG0655	K03809	-
JAMCOH010000023.1	Ribonucleotide reductase of class Ia	COG0209	K10807	-
JAMCOH010000023.1	Transcription initiation factor IIH p5	COG5144	K03144	-
JAMCOH010000023.1	DNA-directed RNA polymerase III su	COG0085	K03021	-
JAMCOH010000023.1	hypothetical protein	COG0655	K03809	-
JAMCOH010000024.1	hypothetical protein		K20643	-
JAMCOH010000024.1	hypothetical protein		K14969	-
JAMCOH010000024.1	hypothetical protein	COG5647	K10609	-
JAMCOH010000024.1	Ubiquinol-cytochrome-c reductase		K00420	-
JAMCOH010000024.1	hypothetical protein	COG0236	K03955	-
JAMCOH010000024.1	SSU ribosomal protein S12p (S23e)	COG0048	K02950	-
JAMCOH010000024.1	hypothetical protein	COG4690	K05758	-
JAMCOH010000024.1	Protein kinase domain		K08832	-
JAMCOH010000024.1	hypothetical protein	COG0443	K09485,K09489	-
JAMCOH010000024.1	hypothetical protein	COG5100	K14015	-
JAMCOH010000024.1	hypothetical protein		K16230	-
JAMCOH010000024.1	Pyridoxal kinase (EC 2.7.1.35)	COG2240	K00868	-
JAMCOH010000024.1	hypothetical protein	COG1893	-	-
JAMCOH010000024.1	Phosphatidylinositol 3- and 4-kinase		K13711	-
JAMCOH010000025.1	23S rRNA (uridine(2552)-2'-O)	COG0293	K02427,K15508	-
JAMCOH010000025.1	hypothetical protein	COG5580	-	-
JAMCOH010000025.1	hypothetical protein		K18179	-
JAMCOH010000025.1	hypothetical protein		K20306	-
JAMCOH010000025.1	proteasome subunit beta2 (EC 3.4.2	COG0638	K02739	-
JAMCOH010000025.1	Serine phosphatase RsbU, regulator of sigma subunit	-		-
JAMCOH010000025.1	hypothetical protein	COG0531	K19564	-
JAMCOH010000025.1	Dethiobiotin synthase BioD (EC 6.3.	COG0132	K01935	-
JAMCOH010000025.1	Adenosylmethionine-8-amino-7-oxo	COG4992	K00833,K19562	-
JAMCOH010000025.1	hypothetical protein		K04563,K05916	-
JAMCOH010000025.1	Cytochrome oxidase biogenesis protein Mss51, requir		K17656	-
JAMCOH010000025.1	hypothetical protein		K10088	-

JAMCOH010000026.1	DNA repair protein RAD51	COG0468	K04482	-
JAMCOH010000026.1	hypothetical protein	COG0143	K15437	-
JAMCOH010000026.1	DNA replication licensing factor MC	COG1241	K02542	-
JAMCOH010000026.1	Glutathione S-transferase, omega (I	COG0435	K07393	-
JAMCOH010000026.1	hypothetical protein	COG1100	K04353,K07836,K07837	-
JAMCOH010000026.1	hypothetical protein		K01078	-
JAMCOH010000026.1	ABC transporter, ATP-binding prote	COG1132	K02021,K05657	-
JAMCOH010000026.1	sterol 3-beta-glucosyltransferase		K05841	GT1
JAMCOH010000026.1	Homoserine O-acetyltransferase (E	COG2021	K00641	-
JAMCOH010000026.1	hypothetical protein		K01078	-
JAMCOH010000027.1	hypothetical protein	COG0513	K17679	-
JAMCOH010000027.1	Peptidyl-tRNA hydrolase (EC 3.1.1.2	COG0193	K01056	-
JAMCOH010000027.1	hypothetical protein		K05730	-
JAMCOH010000027.1	hypothetical protein	COG1485	K18798	-
JAMCOH010000027.1	hypothetical protein		K18083	-
JAMCOH010000027.1	Protoporphyrinogen IX oxidase, aer	COG1232	K00231	-
JAMCOH010000027.1	hypothetical protein		K11094	-
JAMCOH010000027.1	NAD-dependent protein deacetylas	COG0846	K11121	-
JAMCOH010000027.1	LSU ribosomal protein L3e His-243	COG3897	K18803	-
JAMCOH010000027.1	hypothetical protein		K02263	-
JAMCOH010000027.1	NADH-ubiquinone oxidoreductase	COG1143	K03941	-
JAMCOH010000027.1	Nuclear prelamin A recognition fact	COG4624	-	-
JAMCOH010000028.1	hypothetical protein with small GTP-binding domain (		K07889,K07976	-
JAMCOH010000028.1	hypothetical protein	COG0664	K14676,K18550	-
JAMCOH010000028.1	hypothetical protein	COG5251	K03135	-
JAMCOH010000028.1	ATP-dependent protease La (EC 3.4	COG0466	K01338	-
JAMCOH010000028.1	hypothetical protein		K01383,K06005,K06009	-
JAMCOH010000028.1	hypothetical protein		K18466	-
JAMCOH010000028.1	hypothetical protein		K03099	-
JAMCOH010000028.1	hypothetical protein		K11252	-
JAMCOH010000028.1	hypothetical protein	COG5262	K11251	-
JAMCOH010000028.1	Acid Phosphatase		K17619	-
JAMCOH010000028.1	hypothetical protein	COG0666	-	-
JAMCOH010000029.1	hypothetical protein	COG5491	K12193	-
JAMCOH010000029.1	Replication factor C small subunit	COG0470	K10756	-
JAMCOH010000029.1	Peptide-methionine (S)-S-oxide red	COG0225	K07304	-
JAMCOH010000029.1	hypothetical protein		K11842,K11872	-
JAMCOH010000029.1	hypothetical protein		K08139	-
JAMCOH010000029.1	hypothetical protein		K08139	-
JAMCOH010000029.1	hypothetical protein	COG0197	-	-
JAMCOH010000029.1	hypothetical protein		K16750	-
JAMCOH010000029.1	hypothetical protein	COG0484	K17867	-
JAMCOH010000029.1	DNA-directed RNA polymerase I 49 kDa polypeptide (		K03005	-
JAMCOH010000029.1	hypothetical protein (Belongs to the SNF7 family)		K12195	-
JAMCOH010000029.1	Porphobilinogen synthase (EC 4.2.1	COG0113	K01698	-
JAMCOH010000029.1	hypothetical protein		K09660	-
JAMCOH010000029.1	hypothetical protein		K14306	-
JAMCOH010000030.1	25S rRNA (cytosine(2278)-C(5))-met	COG0144	K15264	-
JAMCOH010000030.1	Putative serine/threonine protein kinase		K08286,K08853	-
JAMCOH010000030.1	hypothetical protein	COG0123	K11407	-
JAMCOH010000030.1	Alcohol dehydrogenase (EC 1.1.1.1)	COG1064	K13953	-
JAMCOH010000030.1	5-aminolevulinate synthase (EC 2.3	COG0156	K00643	-
JAMCOH010000030.1	hypothetical protein		K16570	-
JAMCOH010000030.1	hypothetical protein		K15109	-
JAMCOH010000030.1	hypothetical protein	COG1390	K02150	-
JAMCOH010000031.1	hypothetical protein		K22493	-
JAMCOH010000031.1	ISWI chromatin-remodeling comple	COG0553	K11654	-
JAMCOH010000031.1	hypothetical protein		K11427	-
JAMCOH010000031.1	L-serine dehydratase, (PLP)-depend	COG1171	K17989	-
JAMCOH010000031.1	hypothetical protein	COG0652	K01802	-
JAMCOH010000031.1	hypothetical protein		K20352	-

JAMCOH010000031.1	hypothetical protein	COG0267	-	-
JAMCOH010000031.1	Mitochondrial presequence protea:	COG1026	K06972	-
JAMCOH010000031.1	hypothetical protein		K15117	-
JAMCOH010000032.1	Lysine-specific permease	COG0531	K16261	-
JAMCOH010000032.1	DNA topoisomerase I, eukaryotic-ty	COG3569	K03163	-
JAMCOH010000032.1	Ketol-acid reductoisomerase (NAD(	COG0059	K00053	-
JAMCOH010000032.1	Glutathione peroxidase (EC 1.11.1.9	COG0386	K00432	-
JAMCOH010000032.1	Glutathione peroxidase (EC 1.11.1.9	COG0386	K00432	-
JAMCOH010000032.1	Glutathione peroxidase (EC 1.11.1.9	COG0386	K00432	-
JAMCOH010000033.1	Prolyl-tRNA synthetase (EC 6.1.1.15	COG0442	K01881	-
JAMCOH010000033.1	Asparaginyl-tRNA synthetase (EC 6.	COG0017	K01893	-
JAMCOH010000033.1	Chaperone protein DnaK	COG0443	K04043	-
JAMCOH010000033.1	proteasome subunit alpha6 (EC 3.4.	COG0638	K02725	-
JAMCOH010000033.1	hypothetical protein	COG5657	-	-
JAMCOH010000033.1	Nicotinate phosphoribosyltransfera	COG1488	K00763	-
JAMCOH010000033.1	Chaperone protein DnaJ	COG0484	K09518	-
JAMCOH010000034.1	hypothetical protein	COG0143	K15437	-
JAMCOH010000034.1	hypothetical protein	COG0477	-	-
JAMCOH010000034.1	LSU ribosomal protein L27Ae (L15p)	COG0200	K02900,K11273	-
JAMCOH010000034.1	metal-dependent phosphohydrolas	COG1896	K07023	-
JAMCOH010000034.1	sulfhydryl oxidase	COG5054	K17783,K18182	-
JAMCOH010000034.1	GTP cyclohydrolase II (EC 3.5.4.25)	COG0807	K01497	-
JAMCOH010000034.1	Suppressor protein SRP40		K11294	-
JAMCOH010000034.1	Ribonuclease III family	COG0571	-	-
JAMCOH010000034.1	Transcription initiation factor IIH p9	COG1061	K10843	-
JAMCOH010000034.1	Ribosomal protein S21		K17415	-
JAMCOH010000035.1	hypothetical protein		K14311	-
JAMCOH010000035.1	Mevalonate kinase (EC 2.7.1.36)	COG1577	K00869	-
JAMCOH010000035.1	Putative transporter		-	-
JAMCOH010000035.1	Holo-[acyl-carrier protein] synthase (EC 2.7.8.7)		K00997	-
JAMCOH010000035.1	GMP synthase [glutamine-hydrolyz	COG0519	K01951	-
JAMCOH010000035.1	hypothetical protein		K14863	-
JAMCOH010000035.1	hypothetical protein		K19787	-
JAMCOH010000035.1	geranylgeranyltransferase type I alp	COG5536	K05955	-
JAMCOH010000035.1	Arginine methyltransferase		-	-
JAMCOH010000036.1	5'-3' exoribonuclease 2	COG5049	K12619	-
JAMCOH010000036.1	ubiquitin / LSU ribosomal protein L4	COG1552	K02927	-
JAMCOH010000036.1	hypothetical protein		K09291	-
JAMCOH010000037.1	hypothetical protein	arCOG05967	-	-
JAMCOH010000037.1	hypothetical protein		K18468	-
JAMCOH010000037.1	hypothetical protein	COG5147	K09425,K21769	-
JAMCOH010000037.1	Diphthamide biosynthesis protein 3	COG5216	K15455	-
JAMCOH010000037.1	SSU ribosomal protein S8e	COG2007	K02995	-
JAMCOH010000037.1	hypothetical protein	COG0518	K01951,K22314	-
JAMCOH010000037.1	hypothetical protein	COG0604	K18980	-
JAMCOH010000037.1	hypothetical protein	COG0604	K18980	-
JAMCOH010000038.1	hypothetical protein		K15071	-
JAMCOH010000038.1	Thermosome subunit	COG0459	K09499	-
JAMCOH010000038.1	hypothetical protein	COG5024	-	-
JAMCOH010000038.1	hypothetical protein	COG5022	K10352	-
JAMCOH010000038.1	Ubiquinol-cytochrome-c reductase	COG0612	K01412	-
JAMCOH010000038.1	Inositol polyphosphate phosphatase, catalytic domain homologues	COG5411	K01106,K01974	-
JAMCOH010000038.1	hypothetical protein	COG5188	K10777,K12827	-
JAMCOH010000039.1	hypothetical protein		K14550	-
JAMCOH010000039.1	hypothetical protein	COG0234	K04078	-
JAMCOH010000039.1	GTP-binding protein YPT52		K07889,K17785	-
JAMCOH010000039.1	LSU ribosomal protein L1e (L4p)	COG0088	K02930	-
JAMCOH010000039.1	hypothetical protein	COG2340	-	-
JAMCOH010000039.1	iron permease	COG0672	K07243	-

JAMCOH010000039.1	hypothetical protein		K07117	-
JAMCOH010000039.1	hypothetical protein		K17805	-
JAMCOH010000039.1	hypothetical protein		K11968	-
JAMCOH010000039.1	hypothetical protein		K17553	-
JAMCOH010000039.1	hypothetical protein	COG0672	K07243	-
JAMCOH010000039.1	hypothetical protein	COG5537	K06671	-
JAMCOH010000039.1	hypothetical protein	COG5259	K11762	-
JAMCOH010000039.1	hypothetical protein		K20241	-
JAMCOH010000039.1	hypothetical protein		K03248,K20304	-
JAMCOH010000039.1	hypothetical protein		K14263	-
JAMCOH010000039.1	Fibrillarin	COG0724	K14651	-
JAMCOH010000039.1	hypothetical protein		K12385	-
JAMCOH010000039.1	dCMP deaminase (EC 3.5.4.12)	COG2131	K01493	-
JAMCOH010000039.1	Fumarylacetoacetate hydrolase fam	COG0179	K01557	-
JAMCOH010000039.1	tRNA-specific 2-thiouridylase MnmA	COG0482	K21027	-
JAMCOH010000039.1	hypothetical protein		K17553	-
JAMCOH010000039.1	hypothetical protein		K11244	-
JAMCOH010000039.1	hypothetical protein		K18155	-
JAMCOH010000039.1	DNA-directed RNA polymerases I	COG1996	K03009	-
JAMCOH010000039.1	hypothetical protein	COG0323	K08739	-
JAMCOH010000039.1	hypothetical protein	COG1958	K12624	-
JAMCOH010000039.1	hypothetical protein		K11885	-
JAMCOH010000039.1	hypothetical protein	COG5044	K17255	-
JAMCOH010000039.1	Xaa-Pro aminopeptidase (EC 3.4.11	COG0006	K14213	-
JAMCOH010000039.1	proteasome regulatory subunit Rpn	COG1310	K03030	-
JAMCOH010000039.1	hypothetical protein		K12847	-
JAMCOH010000039.1	hypothetical protein		K17715	-
JAMCOH010000039.1	Acetyltransferase (GNAT) domain	COG0454	K19952	-
JAMCOH010000039.1	hypothetical protein		K10752	-
JAMCOH010000039.1	Sulfate transporter	COG0659	-	-
JAMCOH010000039.1	Electron transfer flavoprotein, alph	COG2025	K03522	-
JAMCOH010000039.1	hypothetical protein	COG5026	K00844	-
JAMCOH010000039.1	Glucosamine-6-phosphate deamina	COG0363	K02564	-
JAMCOH010000039.1	N-acetylglucosamine-6-phosphate d	COG1820	K01443	-
JAMCOH010000040.1	Anthranilate phosphoribosyltransfe	COG0547	K00766	-
JAMCOH010000040.1	hypothetical protein	COG1204	K12854	-
JAMCOH010000040.1	Pyruvate dehydrogenase E1 compo	COG0462	K00161	-
JAMCOH010000040.1	hypothetical protein		K11239	-
JAMCOH010000040.1	GTP-binding and nucleic acid-bindir	COG0012	K19788	-
JAMCOH010000040.1	COPI associated protein		-	-
JAMCOH010000040.1	hypothetical protein		K11397	-
JAMCOH010000040.1	hypothetical protein		K06672	-
JAMCOH010000040.1	hypothetical protein		K11402	-
JAMCOH010000041.1	hypothetical protein		K14650	-
JAMCOH010000041.1	hypothetical protein		K17815	-
JAMCOH010000041.1	Citrate synthase (si) (EC 2.3.3.1)	COG0372	K01647	-
JAMCOH010000041.1	hypothetical protein		K17785	-
JAMCOH010000041.1	hypothetical protein		K14169	-
JAMCOH010000041.1	hypothetical protein	COG2453	K14819	-
JAMCOH010000042.1	LSU ribosomal protein L34e	COG2174	K02915	-
JAMCOH010000042.1	RidA/YER057c/UK114 superfamily p	COG0251	-	-
JAMCOH010000042.1	hypothetical protein (Component of the coat protein		K14005	-
JAMCOH010000042.1	hypothetical protein	COG1554	K01194	GH37
JAMCOH010000042.1	hypothetical protein	COG0564	K15453	-
JAMCOH010000042.1	Eukaryotic translation initiation fact	COG1093	K03237	-
JAMCOH010000042.1	hypothetical protein	COG4886	-	-
JAMCOH010000043.1	hypothetical protein	COG0366	K01182,K01187	GH13,GH31
JAMCOH010000043.1	hypothetical protein		K22241	-
JAMCOH010000043.1	hypothetical protein		K12669	-

JAMCOH010000043.1	hypothetical protein		K08576	-
JAMCOH010000043.1	Mannosyltransferase		K05286	GT22
JAMCOH010000043.1	Alcohol dehydrogenase (EC 1.1.1.1)	COG1064	K13953	-
JAMCOH010000043.1	Oligopeptide transport ATP-binding protein OppF (TC		K08340	-
JAMCOH010000043.1	hypothetical protein		K02646	-
JAMCOH010000043.1	hypothetical protein		K17798	-
JAMCOH010000043.1	hypothetical protein		K06641	-
JAMCOH010000043.1	hypothetical protein		K20474	-
JAMCOH010000043.1	Thermosome subunit	COG0459	K09495	-
JAMCOH010000043.1	Dephospho-CoA kinase (EC 2.7.1.24	COG0237	K00859	-
JAMCOH010000043.1	hypothetical protein	COG1893	K00077,K00511	-
JAMCOH010000043.1	hypothetical protein	COG5126	K06268	-
JAMCOH010000043.1	hypothetical protein		K12572	-
JAMCOH010000043.1	hypothetical protein		K21197	-
JAMCOH010000043.1	hypothetical protein		K04630,K04640,K19860	-
JAMCOH010000043.1	hypothetical protein	COG0612	K00414	-
JAMCOH010000043.1	hypothetical protein	COG0049	K02992	-
JAMCOH010000043.1	hypothetical protein		K00237	-
JAMCOH010000043.1	hypothetical protein	COG0199	K02954	-
JAMCOH010000043.1	Riboflavin synthase eubacterial/euk	COG0307	K00793	-
JAMCOH010000043.1	proteasome subunit beta4 (EC 3.4.2	COG0638	K02734	-
JAMCOH010000043.1	ATP phosphoribosyltransferase (EC	COG0040	K00765	-
JAMCOH010000043.1	Dehydrodolichyl diphosphate synth	COG0020	K11778	-
JAMCOH010000043.1	hypothetical protein		K14818	-
JAMCOH010000043.1	DNA primase small subunit (EC 2.7.	COG1467	K02684	-
JAMCOH010000043.1	ATP-dependent helicase HrpA	COG1643	K12818	-
JAMCOH010000043.1	proteasome regulatory subunit Rpn	COG5071	K03035	-
JAMCOH010000043.1	hypothetical protein		K12393	-
JAMCOH010000043.1	Dihydroxy-acid dehydratase (EC 4.2	COG0129	K01687	-
JAMCOH010000043.1	ubiquitin-conjugating enzyme	COG5078	K10689	-
JAMCOH010000043.1	hypothetical protein		K11132	-
JAMCOH010000043.1	Dihydrosphingosine delta-4 desaturase (EC 1.14.99.-)		K04712	-
JAMCOH010000043.1	Histidyl-tRNA synthetase (EC 6.1.1.2	COG0124	K01892	-
JAMCOH010000044.1	hypothetical protein	COG1584	K07034	-
JAMCOH010000044.1	nitrilase superfamily	COG0388	K01431,K11206	-
JAMCOH010000044.1	hypothetical protein	COG0642	K00898	-
JAMCOH010000044.1	hypothetical protein		K13344	-
JAMCOH010000044.1	hypothetical protein	COG0724	K14407	-
JAMCOH010000044.1	proteasome regulatory subunit Rpn	COG5187	K03037	-
JAMCOH010000044.1	Acyl-CoA dehydrogenase	COG3173	K00249	-
JAMCOH010000044.1	hypothetical protein		K12620	-
JAMCOH010000044.1	tRNA (adenine(58)-N(1))-methyltran	COG2519	K07442	-
JAMCOH010000044.1	Actin-related protein 2	COG5277	K17260	-
JAMCOH010000044.1	Protein tyrosine kinase		K08866	-
JAMCOH010000045.1	Allophanate hydrolase (EC 3.5.1.54)	COG0154	K14541	-
JAMCOH010000045.1	Histone acetyltransferase type B, catalytic subunit (EC		K11303	-
JAMCOH010000045.1	hypothetical protein		K12780	-
JAMCOH010000045.1	hypothetical protein	COG2234	K01301	-
JAMCOH010000045.1	hypothetical protein		K14854	-
JAMCOH010000045.1	Homocitrate synthase (EC 2.3.3.14)	COG0119	K01655	-
JAMCOH010000046.1	hypothetical protein	COG1100	K07937,K07977	-
JAMCOH010000046.1	Enoyl-CoA hydratase/isomerase	COG1024	K13239	-
JAMCOH010000046.1	hypothetical protein	COG3938	K20406	-
JAMCOH010000046.1	Enoyl-CoA hydratase/isomerase	COG1024	K13239	-
JAMCOH010000046.1	Pre-mRNA splicing factor PRP8	COG5178	K12856	-
JAMCOH010000046.1	Aminotransferase class I and II	COG0156	K00654	-
JAMCOH010000046.1	hypothetical protein	COG0013	K03355	-
JAMCOH010000046.1	hypothetical protein		K22139	-
JAMCOH010000047.1	hypothetical protein	COG1052	K00015	-

JAMCOH010000047.1	hypothetical protein	COG1104	K01763,K20247	-
JAMCOH010000047.1	Threonyl-tRNA synthetase (EC 6.1.1	COG0441	K01868	-
JAMCOH010000047.1	hypothetical protein	COG5157	K15175	-
JAMCOH010000047.1	hypothetical protein		K08329	-
JAMCOH010000047.1	hypothetical protein		K13103	-
JAMCOH010000047.1	hypothetical protein	COG1052	K00015	-
JAMCOH010000047.1	hypothetical protein		K17782	-
JAMCOH010000047.1	hypothetical protein		K07897	-
JAMCOH010000047.1	hypothetical protein	COG5143	K08516	-
JAMCOH010000047.1	hypothetical protein		K01120	-
JAMCOH010000048.1	Peptidyl-prolyl cis-trans isomerase (	COG0652	K05864	-
JAMCOH010000048.1	DNA-directed RNA polymerase II lar	COG0086	K03006	-
JAMCOH010000048.1	DNA-directed RNA polymerase III la	COG0086	K03018,K21594	-
JAMCOH010000048.1	Sodium/bile acid symporter family	COG0385	K02731,K14347	-
JAMCOH010000048.1	Septum formation protein Maf	COG0424	K06287	-
JAMCOH010000048.1	hypothetical protein	COG5411	K20279	-
JAMCOH010000048.1	hypothetical protein	COG5540	-	-
JAMCOH010000048.1	Indoleamine 2,3-dioxygenase (EC 1.13.11.52)		K00463	-
JAMCOH010000049.1	hypothetical protein	COG1537	K06965	-
JAMCOH010000049.1	hypothetical protein	COG5126	-	-
JAMCOH010000049.1	hypothetical protein	COG1196	-	-
JAMCOH010000049.1	Glutamyl-tRNA synthetase (EC 6.1.1	COG0008	K01885	-
JAMCOH010000049.1	hypothetical protein		K08501	-
JAMCOH010000049.1	tRNA(Phe) 7-((3-amino-3-carboxypr	COG1590	K15450	-
JAMCOH010000049.1	Aspartyl-tRNA synthetase (EC 6.1.1.	COG0017	K01876,K22503	-
JAMCOH010000049.1	hypothetical protein		K16186	-
JAMCOH010000049.1	hypothetical protein		K01164	-
JAMCOH010000049.1	hypothetical protein	COG5211	K15544	-
JAMCOH010000050.1	hypothetical protein		K18156	-
JAMCOH010000050.1	Spermidine synthase (EC 2.5.1.16)	COG0421	K00797	-
JAMCOH010000050.1	Adenosine deaminase (EC 3.5.4.4)	COG1816	K01488	-
JAMCOH010000050.1	Ankyrin repeat-containing protein Y	COG0666	K06867	-
JAMCOH010000050.1	Ribosomal protein L9		-	-
JAMCOH010000050.1	hypothetical protein		K12831	-
JAMCOH010000050.1	Quinone oxidoreductase (EC 1.6.5.5	COG0604	K00344	-
JAMCOH010000050.1	hypothetical protein	COG1398	K00507	-
JAMCOH010000051.1	hypothetical protein		K08287	-
JAMCOH010000051.1	18S rRNA (adenine(1779)-N(6)/ader	COG0030	K14191	-
JAMCOH010000051.1	hypothetical protein		K11553	-
JAMCOH010000051.1	Actin	COG5277	K05692	-
JAMCOH010000051.1	hypothetical protein	COG5143	K08513	-
JAMCOH010000051.1	hypothetical protein	COG5143	K08513	-
JAMCOH010000051.1	Glycine cleavage system H protein	COG0509	K02437	-
JAMCOH010000051.1	soluble fumarate reductase, cytopla	COG1053	K18561	-
JAMCOH010000051.1	DNA repair and recombination prot	COG0553	K10875	-
JAMCOH010000051.1	hypothetical protein		K09281	-
JAMCOH010000052.1	hypothetical protein		K20476	-
JAMCOH010000052.1	hypothetical protein		K03950	-
JAMCOH010000052.1	Tubulin beta chain	COG5023	K07375	-
JAMCOH010000052.1	hypothetical protein	COG0457	K02993	-
JAMCOH010000052.1	Translation elongation factor Tu	COG0050	K02358	-
JAMCOH010000052.1	Translation elongation factor Tu	COG0050	K02358	-
JAMCOH010000052.1	hypothetical protein		K03348	-
JAMCOH010000052.1	Phosphatidylserine decarboxylase (	COG0688	K01613	-
JAMCOH010000053.1	hypothetical protein	COG0328	K03469	-
JAMCOH010000053.1	hypothetical protein	COG0591	K20989	-
JAMCOH010000053.1	Vacuolar protein sorting-associated	COG5158	K12479,K20367	-
JAMCOH010000053.1	Isoleucyl-tRNA synthetase (EC 6.1.1	COG0060	K01870	-
JAMCOH010000053.1	tRNA(Ile)-lysidine synthetase (EC 6.	COG0037	K04075	-
JAMCOH010000053.1	hypothetical protein		K03128	-
JAMCOH010000054.1	hypothetical protein		K22493	-

JAMCOH010000054.1	hypothetical protein		K03858	-
JAMCOH010000054.1	hypothetical protein	COG5118	K15198	-
JAMCOH010000054.1	High-affnity carbon uptake protein Hat/HatR		-	-
JAMCOH010000054.1	Homoisocitrate dehydrogenase (EC	COG0473	K05824	-
JAMCOH010000054.1	Heat shock protein GrpE	COG0576	K03687	-
JAMCOH010000054.1	hypothetical protein	COG0184	K02956	-
JAMCOH010000054.1	hypothetical protein		K11562	-
JAMCOH010000054.1	hypothetical protein		K15296	-
JAMCOH010000055.1	hypothetical protein	COG1587	K01719	-
JAMCOH010000055.1	Acetyl-CoA synthetase (EC 6.2.1.1)	COG0365	K01895	-
JAMCOH010000055.1	Porphobilinogen deaminase (EC 2.5	COG0181	K01749	-
JAMCOH010000055.1	hypothetical protein	COG1948	K10848	-
JAMCOH010000055.1	hypothetical protein		K15364	-
JAMCOH010000055.1	5,10-methylenetetrahydrofolate re	COG0685	K00297	-
JAMCOH010000055.1	hypothetical protein		K21141	-
JAMCOH010000055.1	Malate dehydrogenase (EC 1.1.1.37	COG0039	K00026	-
JAMCOH010000056.1	hypothetical protein		K13348,K20311	-
JAMCOH010000056.1	hypothetical protein		K07976	-
JAMCOH010000056.1	hypothetical protein	COG5260	K03514,K12596	-
JAMCOH010000056.1	hypothetical protein		K11118	-
JAMCOH010000056.1	hypothetical protein		K18712	-
JAMCOH010000057.1	hypothetical protein	COG5193	K18757	-
JAMCOH010000057.1	hypothetical protein		K13350	-
JAMCOH010000057.1	hypothetical protein	COG0526	K09580	-
JAMCOH010000057.1	hypothetical protein		K22382	-
JAMCOH010000058.1	hypothetical protein		K14284	-
JAMCOH010000058.1	hypothetical protein		K17278	-
JAMCOH010000058.1	hypothetical protein	COG0456	K20793	-
JAMCOH010000058.1	hypothetical protein	COG5407	K09540	-
JAMCOH010000058.1	IMP cyclohydrolase (EC 3.5.4.10) / I	COG0138	K00602	-
JAMCOH010000058.1	LSU ribosomal protein L15e	COG1632	K02877	-
JAMCOH010000058.1	hypothetical protein		K14763	-
JAMCOH010000058.1	DNA mismatch repair protein MutS	COG0249	-	-
JAMCOH010000059.1	hypothetical protein		K19613	-
JAMCOH010000059.1	hypothetical protein		K19613	-
JAMCOH010000059.1	hypothetical protein		K19613	-
JAMCOH010000059.1	Glutathione S-transferase (EC 2.5.1.	COG0625	K00799	-
JAMCOH010000059.1	Xaa-Pro aminopeptidase (EC 3.4.11	COG0006	K18573	-
JAMCOH010000059.1	hypothetical protein	COG2828	K08597	-
JAMCOH010000059.1	proteasome subunit alpha3 (EC 3.4.	COG0638	K02728	-
JAMCOH010000059.1	hypothetical protein		K18158	-
JAMCOH010000059.1	Sphingosine-1-phosphate lyase (EC	COG0076	K01634	-
JAMCOH010000059.1	hypothetical protein		K12881	-
JAMCOH010000059.1	hypothetical protein		K12881	-
JAMCOH010000059.1	hypothetical protein	COG4266	K13354	-
JAMCOH010000059.1	hypothetical protein		K15114	-
JAMCOH010000059.1	SSU ribosomal protein S15e (S19p)	COG0185	K02958	-
JAMCOH010000059.1	LSU ribosomal protein P2 (L7/L12)	COG2058	K02943	-
JAMCOH010000059.1	hypothetical protein		K18716	-
JAMCOH010000059.1	hypothetical protein	COG5254	K21848	-
JAMCOH010000059.1	hypothetical protein	COG5594	K21989	-
JAMCOH010000059.1	DNA topoisomerase I (EC 5.99.1.2)	COG0550	K03165	-
JAMCOH010000059.1	hypothetical protein	COG1112	K10706	-
JAMCOH010000059.1	hypothetical protein	COG0445	K20366	-
JAMCOH010000059.1	hypothetical protein		K11971	-
JAMCOH010000059.1	hypothetical protein		K13199	-
JAMCOH010000059.1	hypothetical protein	COG5307	K18442	-
JAMCOH010000059.1	Acyl carrier protein of FASI / 3-oxoa	COG0331	K00667	-
JAMCOH010000060.1	GTP-binding and nucleic acid-binding protein YchF		K11234	-
JAMCOH010000060.1	hypothetical protein	COG5536	K14050	-
JAMCOH010000060.1	hypothetical protein		K02537	-

JAMCOH010000060.1	hypothetical protein	COG5333	K05349,K15564	GH3
JAMCOH010000060.1	hypothetical protein		K15040	-
JAMCOH010000060.1	hypothetical protein		K13107	-
JAMCOH010000060.1	hypothetical protein		K14824	-
JAMCOH010000061.1	Glycosyltransferase	COG0438	K03857	GT4
JAMCOH010000061.1	hypothetical protein	COG5244	K04648	-
JAMCOH010000061.1	hypothetical protein	COG5126	K16466	-
JAMCOH010000061.1	hypothetical protein		K02895	-
JAMCOH010000061.1	proteasome regulatory subunit Rpt	COG1222	K03064	-
JAMCOH010000061.1	hypothetical protein	COG1208	K03241	-
JAMCOH010000061.1	hypothetical protein	COG5143	K08517	-
JAMCOH010000061.1	hypothetical protein		K20195	-
JAMCOH010000061.1	hypothetical protein	COG0233	K02838	-
JAMCOH010000061.1	hypothetical protein		K14827	-
JAMCOH010000061.1	hypothetical protein	COG5085	-	-
JAMCOH010000062.1	hypothetical protein	COG0624	K01293	-
JAMCOH010000062.1	hypothetical protein	COG1131	K08711,K08712	-
JAMCOH010000063.1	hypothetical protein		K08336	-
JAMCOH010000063.1	Beta-glucosidase (SUN family)		K01238	-
JAMCOH010000063.1	hypothetical protein	COG0642	K11233	-
JAMCOH010000063.1	hypothetical protein	COG0488	-	-
JAMCOH010000063.1	Serine palmitoyltransferase, subuni	COG0156	K00654	-
JAMCOH010000064.1	Putative sodium:solute symporter,	COG0591	K20989	-
JAMCOH010000064.1	hypothetical protein	COG5226	-	-
JAMCOH010000064.1	hypothetical protein		K01530	-
JAMCOH010000064.1	ATP synthase alpha chain (EC 3.6.3.	COG0056	K02132	-
JAMCOH010000064.1	hypothetical protein		K11372	-
JAMCOH010000064.1	hypothetical protein	COG2513	K01637	-
JAMCOH010000064.1	hypothetical protein		K06655,K08282	-
JAMCOH010000064.1	tRNA(Ser) (uridine(44)-2'-O)-methyltransferase (		K15447	-
JAMCOH010000064.1	hypothetical protein	COG5109	-	-
JAMCOH010000064.1	hypothetical protein		K03872	-
JAMCOH010000064.1	hypothetical protein		K20180	-
JAMCOH010000064.1	hypothetical protein		K11551	-
JAMCOH010000064.1	hypothetical protein	COG5422	K19842,K19843	-
JAMCOH010000064.1	hypothetical protein	COG1028	-	-
JAMCOH010000064.1	hypothetical protein	COG1028	K16216	-
JAMCOH010000064.1	hypothetical protein	COG1028	K16216	-
JAMCOH010000064.1	Adenylate kinase (EC 2.7.4.3)	COG0563	K13800	-
JAMCOH010000064.1	hypothetical protein	COG0760	K09578	-
JAMCOH010000064.1	Puromycin-sensitive aminopeptidas	COG0308	K13721	-
JAMCOH010000064.1	hypothetical protein		K14573	-
JAMCOH010000064.1	Serine/threonine protein phosphat	COG0639	K15427,K15498	-
JAMCOH010000064.1	hypothetical protein		K02963	-
JAMCOH010000064.1	hypothetical protein		K22255	-
JAMCOH010000064.1	proteasome regulatory subunit Rpn4		K03034	-
JAMCOH010000064.1	Phosphoglycerate mutase (EC 5.4.2	COG0588	K01834	-
JAMCOH010000064.1	DnaK-like protein	COG0443	K03283	-
JAMCOH010000064.1	Acetyl-CoA synthetase (EC 6.2.1.1)	COG0365	K01895	-
JAMCOH010000064.1	hypothetical protein	COG5542	K07542	GT76
JAMCOH010000064.1	hypothetical protein		K03846	GT22
JAMCOH010000064.1	hypothetical protein	COG2036	K11253	-
JAMCOH010000064.1	hypothetical protein	COG2036	K11254	-
JAMCOH010000064.1	hypothetical protein		K06691	-
JAMCOH010000064.1	hypothetical protein		K06691	-
JAMCOH010000064.1	hypothetical protein	COG1546	-	-
JAMCOH010000064.1	hypothetical protein	COG5019	K16944	-
JAMCOH010000064.1	hypothetical protein		K20724	-
JAMCOH010000064.1	phosphatidylinositol deacylase activity		K05294	-
JAMCOH010000064.1	hypothetical protein		K11252	-

JAMCOH010000064.1	hypothetical protein	COG5262	K11251	-
JAMCOH010000064.1	hypothetical protein	COG0494	K03574,K17816	-
JAMCOH010000064.1	hypothetical protein	COG0667	K17647	-
JAMCOH010000065.1	hypothetical protein	COG0642	-	-
JAMCOH010000065.1	hypothetical protein		K10276	-
JAMCOH010000065.1	Argininosuccinate synthase (EC 6.3.	COG0137	K01940	-
JAMCOH010000065.1	Biotin carboxylase of acetyl-CoA ca	COG0439	K11262	-
JAMCOH010000066.1	Ferric reductase		-	-
JAMCOH010000066.1	Ferric reductase (1.6.99.14)		-	-
JAMCOH010000066.1	hypothetical protein	COG0507	K03507,K15255	-
JAMCOH010000066.1	hypothetical protein		K20475	-
JAMCOH010000066.1	hypothetical protein		K12622	-
JAMCOH010000066.1	LSU ribosomal protein L47mt, mitochondrial		K17428	-
JAMCOH010000067.1	hypothetical protein	COG0666	K06867	-
JAMCOH010000067.1	hypothetical protein	COG5028	K14007	-
JAMCOH010000067.1	hypothetical protein		K15109	-
JAMCOH010000067.1	hypothetical protein	COG2423	-	-
JAMCOH010000068.1	hypothetical protein	COG5638	-	-
JAMCOH010000068.1	hypothetical protein		K14822	-
JAMCOH010000068.1	LSU ribosomal protein L7Ae	COG1358	K02936	-
JAMCOH010000069.1	Succinate dehydrogenase flavoprot	COG1053	K00234	-
JAMCOH010000069.1	hypothetical protein		K22493	-
JAMCOH010000070.1	hypothetical protein	COG1793	K10777	-
JAMCOH010000070.1	hypothetical protein		K12609	-
JAMCOH010000070.1	Ribonucleotide reductase of class Ia	COG1678	K10808	-
JAMCOH010000070.1	Ribosome biogenesis protein TSR3	COG2042	K09140	-
JAMCOH010000070.1	Aldehyde dehydrogenase (EC 1.2.1.	COG1012	K00128,K00129	-
JAMCOH010000071.1	hypothetical protein		K22493	-
JAMCOH010000071.1	hypothetical protein	COG4178	K15628	-
JAMCOH010000071.1	Nicotinate-nucleotide adenylyltrans	COG1057	K06210	-
JAMCOH010000071.1	3-isopropylmalate dehydratase larg	COG0065	K01702	-
JAMCOH010000071.1	hypothetical protein	COG5647	K03869	-
JAMCOH010000071.1	hypothetical protein		K11279	-
JAMCOH010000071.1	chitin deacetylase	COG0726	K01452	-
JAMCOH010000071.1	Uncharacterized protein YqjZ	COG2329	-	-
JAMCOH010000071.1	Serine/threonine protein kinase		K19852	-
JAMCOH010000071.1	hypothetical protein		K02139	-
JAMCOH010000071.1	hypothetical protein	COG1958	K12625	-
JAMCOH010000071.1	hypothetical protein		K14310	-
JAMCOH010000071.1	hypothetical protein	COG2124	K00493	-
JAMCOH010000071.1	hypothetical protein	COG2124	K00493	-
JAMCOH010000071.1	Phosphoenolpyruvate carboxykinas	COG1866	K01610	-
JAMCOH010000071.1	Beta-hexosaminidase (EC 3.2.1.52)	COG1472	K01207	-
JAMCOH010000071.1	hypothetical protein	COG0258	K15339	-
JAMCOH010000071.1	hypothetical protein		K01183	GH18
JAMCOH010000071.1	3-ketoacyl-CoA thiolase (EC 2.3.1.1	COG0183	K07513	-
JAMCOH010000071.1	Aspartate aminotransferase (EC 2.6	COG0436	-	-
JAMCOH010000071.1	hypothetical protein		K04464,K19847	-
JAMCOH010000071.1	hypothetical protein	COG2319	-	-
JAMCOH010000071.1	hypothetical protein		K11322	-
JAMCOH010000071.1	hypothetical protein		K05740,K11238,K17971	-
JAMCOH010000071.1	hypothetical protein	COG0702	-	-
JAMCOH010000071.1	Formate--tetrahydrofolate ligase (E	COG2759	K00288	-
JAMCOH010000071.1	Belongs to the peroxidase family	COG0685	K00428	-
JAMCOH010000071.1	Ascorbate peroxidase (EC 1.11.1.11	COG0685	K00428	-
JAMCOH010000072.1	Reverse transcriptase (RNA-dependent DNA polymerase)		-	-
JAMCOH010000073.1	Belongs to the glycosyl hydrolase 5 (cellulase A) family	COG2730	K01210	-

JAMCOH010000073.1	hypothetical protein	COG1205	K06877	-
JAMCOH010000073.1	hypothetical protein	COG5096	K11825	-
JAMCOH010000073.1	COG1355, Predicted dioxygenase	COG1355	K06990	-
JAMCOH010000073.1	hypothetical protein		K12462	-
JAMCOH010000073.1	hypothetical protein		K08293	-
JAMCOH010000074.1	Sphingomyelin phosphodiesterase		K01117	-
JAMCOH010000074.1	hypothetical protein (Belongs to the syntaxin family)		K08490	-
JAMCOH010000074.1	Vps20/32/60-like protein (ESCRT-III)		K12194	-
JAMCOH010000074.1	hypothetical protein	COG5108	K10908	-
JAMCOH010000074.1	D-Lactate dehydrogenase, cytochro	COG0277	K00102	-
JAMCOH010000075.1	Diphthamide biosynthesis protein 2	COG1736	K17866	-
JAMCOH010000075.1	hypothetical protein		K15014	-
JAMCOH010000075.1	hypothetical protein		K05389	-
JAMCOH010000075.1	hypothetical protein	COG0186	K02961	-
JAMCOH010000075.1	Histidinol-phosphate aminotransfer	COG0079	K00817	-
JAMCOH010000075.1	hypothetical protein	COG0457	K12274	-
JAMCOH010000076.1	hypothetical protein	COG0531	-	-
JAMCOH010000076.1	hypothetical protein		K00223	-
JAMCOH010000076.1	hypothetical protein		K12947	-
JAMCOH010000076.1	hypothetical protein	COG1028	-	-
JAMCOH010000076.1	hypothetical protein		K05747,K20521	-
JAMCOH010000076.1	hypothetical protein	COG0591	K20989	-
JAMCOH010000076.1	hypothetical protein		K01383,K06005,K06009	-
JAMCOH010000077.1	hypothetical protein	COG0814	K14209	-
JAMCOH010000077.1	hypothetical protein	COG0531	-	-
JAMCOH010000078.1	hypothetical protein	COG0738	-	-
JAMCOH010000078.1	hypothetical protein	COG1902	K00354	-
JAMCOH010000078.1	hypothetical protein		K11324	-
JAMCOH010000078.1	hypothetical protein	COG5087	K11309	-
JAMCOH010000078.1	hypothetical protein	COG0271	-	-
JAMCOH010000079.1	Mannose-1-phosphate guanylyltran	COG1208	K00966	-
JAMCOH010000079.1	hypothetical protein	COG2313	K16329,K16330	-
JAMCOH010000079.1	hypothetical protein	COG2313	K16329,K16330	-
JAMCOH010000079.1	Pseudouridine 5'-phosphate g	COG2313	K16329,K16330	-
JAMCOH010000079.1	hypothetical protein		K17780	-
JAMCOH010000079.1	hypothetical protein		K10882	-
JAMCOH010000079.1	Fructose-1,6-bisphosphatase, type	COG0158	K03841	-
JAMCOH010000080.1	Ferric reductase		-	-
JAMCOH010000080.1	hypothetical protein	COG1131	K08711,K08712	-
JAMCOH010000080.1	hypothetical protein	COG1131	K08711,K08712	-
JAMCOH010000080.1	hypothetical protein		K20410	-
JAMCOH010000081.1	hypothetical protein	COG0062	K12615	-
JAMCOH010000081.1	hypothetical protein		K03352	-
JAMCOH010000081.1	CDP-diacylglycerol--inositol 3-phosp	COG0558	K00999	-
JAMCOH010000081.1	hypothetical protein		K20179	-
JAMCOH010000081.1	hypothetical protein		K06695	-
JAMCOH010000081.1	hypothetical protein		K21768	-
JAMCOH010000081.1	Translation machinery-associated p	COG2016	K07575	-
JAMCOH010000081.1	Putative ATP-dependent RNA helica	COG1111	K14635	-
JAMCOH010000081.1	GTPase Nug1p, which associates wi	COG1161	K14538	-
JAMCOH010000081.1	hypothetical protein	COG5371	K14642	-
JAMCOH010000081.1	hypothetical protein		K19983	-
JAMCOH010000081.1	putative 3 beta-hydroxysteroid deh	COG0451	K07748	-
JAMCOH010000081.1	hypothetical protein		K11593	-
JAMCOH010000081.1	hypothetical protein		K18718	-
JAMCOH010000081.1	Oxidoreductase, short-chain dehyd	COG1028	-	-
JAMCOH010000081.1	hypothetical protein		K17978	-
JAMCOH010000081.1	NADP-specific glutamate dehydrog	COG0334	K00262	-
JAMCOH010000081.1	Tryptophan synthase beta chain (E	COG0133	K01694	-
JAMCOH010000081.1	Mannosyl-oligosaccharide glucosidase (EC 3.2.1.106)		K01228	-

JAMCOH010000081.1	hypothetical protein		K15218	-
JAMCOH010000081.1	Succinate-acetate/proton symporte	COG1584	K07034	-
JAMCOH010000081.1	hypothetical protein	COG1028	K06123	-
JAMCOH010000081.1	2-oxoglutarate dehydrogenase E1 c	COG0567	K00164	-
JAMCOH010000081.1	hypothetical protein	COG5192	K14569	-
JAMCOH010000082.1	hypothetical protein		K13341	-
JAMCOH010000082.1	Choline kinase (EC 2.7.1.32)	COG0510	K00866,K00894	-
JAMCOH010000083.1	FAD dependent oxidoreductase	COG0665	-	-
JAMCOH010000083.1	hypothetical protein	COG5114	K11314	-
JAMCOH010000083.1	SSU ribosomal protein S17e	COG1383	K02962	-
JAMCOH010000083.1	hypothetical protein	COG5191	K14557	-
JAMCOH010000084.1	Chromosome partition protein smc	COG1196	K06674	-
JAMCOH010000084.1	hypothetical protein		K05533	GT62
JAMCOH010000085.1	Long-chain-fatty-acid--CoA ligase (E	COG1022	K01897	-
JAMCOH010000085.1	Serine threonine-protein phosphatase	COG0639	K06269	-
JAMCOH010000085.1	hypothetical protein	COG1131	K08712	-
JAMCOH010000086.1	Thermosome subunit	COG0459	K09494	-
JAMCOH010000086.1	hypothetical protein		K14684	-
JAMCOH010000086.1	tRNA-dihydrouridine(16/17) syntha	COG0042	K05542	-
JAMCOH010000087.1	hypothetical protein	COG5379	K13621	-
JAMCOH010000087.1	GDP-mannose 4,6 dehydratase	COG0451	K17741	-
JAMCOH010000088.1	Lysine-specific permease	COG0531	K03293	-
JAMCOH010000088.1	AAA family ATPase Cdc48	COG0464	K13525	-
JAMCOH010000088.1	Histidine triad (HIT) nucleotide-bind	COG0537	K01518	-
JAMCOH010000088.1	hypothetical protein	COG5102	-	-
JAMCOH010000088.1	Amino acid permease	COG0531	K16261	-
JAMCOH010000089.1	hypothetical protein		K20044	-
JAMCOH010000089.1	hypothetical protein	COG5105	K18064,K18065	-
JAMCOH010000089.1	Phosphocholine cytidylyltransferase	COG0615	K00968	-
JAMCOH010000089.1	hypothetical protein		K17764	-
JAMCOH010000089.1	hypothetical protein	COG4581	K20223	-
JAMCOH010000090.1	hypothetical protein		K17434	-
JAMCOH010000090.1	hypothetical protein		K21849	-
JAMCOH010000090.1	hypothetical protein		K15457,K15458,K15501	-
JAMCOH010000091.1	Glutamine synthetase type II, eukar	COG0174	K01915	-
JAMCOH010000091.1	hypothetical protein	COG5231	K02144	-
JAMCOH010000092.1	Ferric reductase		-	-
JAMCOH010000092.1	hypothetical protein	COG0605	K04564	-
JAMCOH010000092.1	Superoxide dismutase [Mn] (EC 1.1	COG0605	K04564	-
JAMCOH010000092.1	hypothetical protein		K17969	-
JAMCOH010000092.1	hypothetical protein		K15440	-
JAMCOH010000092.1	hypothetical protein	COG0666	K06867	-
JAMCOH010000092.1	Ribulose-phosphate 3-epimerase (E	COG0036	K01783	-
JAMCOH010000092.1	hypothetical protein		K14814	-
JAMCOH010000093.1	Glycogen [starch] synthase, eukaryo	COG0438	K00693	GT3
JAMCOH010000093.1	WD repeat protein		K14558	-
JAMCOH010000093.1	hypothetical protein		K12594	-
JAMCOH010000093.1	hypothetical protein	COG1739	-	-
JAMCOH010000093.1	hypothetical protein	COG1739	-	-
JAMCOH010000093.1	hypothetical protein	COG0457	-	-
JAMCOH010000093.1	hypothetical protein		K00706	GT48
JAMCOH010000093.1	hypothetical protein	COG5273	K20003	-
JAMCOH010000093.1	hypothetical protein		K13682	GT91
JAMCOH010000093.1	hypothetical protein	COG1584	K07034	-
JAMCOH010000093.1	hypothetical protein		K17676	-
JAMCOH010000093.1	tRNA (guanine(26)-N(2))-dimethyltr	COG1867	K00555	-
JAMCOH010000093.1	hypothetical protein	COG0642	K11231,K19691	-
JAMCOH010000093.1	hypothetical protein	COG0681	K13280	-

JAMCOH010000093.1	Phosphopantothenoylcysteine deca	COG0452	K00652,K01598	-
JAMCOH010000093.1	hypothetical protein		K05755	-
JAMCOH010000093.1	hypothetical protein	COG5104	K12821	-
JAMCOH010000093.1	Acyl-CoA-dependent ceramide synt	COG5058	K04709	-
JAMCOH010000093.1	hypothetical protein		K16738	-
JAMCOH010000093.1	hypothetical protein	COG0477	-	-
JAMCOH010000093.1	hypothetical protein		K11269	-
JAMCOH010000093.1	hypothetical protein		K09827	-
JAMCOH010000093.1	hypothetical protein	COG2319	K20409	-
JAMCOH010000093.1	Transcription initiation factor IID 23	COG5162	K03134	-
JAMCOH010000093.1	Signal recognition particle protein F	COG0541	K03106	-
JAMCOH010000093.1	hypothetical protein		K18185	-
JAMCOH010000093.1	hypothetical protein	COG0477	-	-
JAMCOH010000093.1	hypothetical protein		K04705	-
JAMCOH010000093.1	hypothetical protein		K08505	-
JAMCOH010000093.1	hypothetical protein		K10364	-
JAMCOH010000093.1	hypothetical protein	COG5021	K10590	-
JAMCOH010000094.1	S-adenosylmethionine synthetase (	COG0192	K00789	-
JAMCOH010000094.1	hypothetical protein		K11272	-
JAMCOH010000094.1	hypothetical protein	COG1094	K06961	-
JAMCOH010000095.1	poly(A) polymerase	COG5186	K14376	-
JAMCOH010000095.1	hypothetical protein	COG5032	K19801	-
JAMCOH010000095.1	hypothetical protein		K22285	-
JAMCOH010000095.1	hypothetical protein		K09359	-
JAMCOH010000096.1	Inorganic pyrophosphatase (EC 3.6.	COG0221	K01507	-
JAMCOH010000096.1	hypothetical protein	COG5594	K21989	-
JAMCOH010000096.1	hypothetical protein	COG0672	K07243	-
JAMCOH010000097.1	hypothetical protein	COG0474	K01536	-
JAMCOH010000097.1	Dolichyl-phosphate-mannose-prote	COG1928	K00728	GT39
JAMCOH010000098.1	hypothetical protein		K19984	-
JAMCOH010000098.1	ubiquitin / SSU ribosomal protein S	COG5272	K02977	-
JAMCOH010000098.1	hypothetical protein	COG0526	-	-
JAMCOH010000099.1	GTP-binding protein RHO3	COG1100	K07975	-
JAMCOH010000099.1	hypothetical protein	COG5597	-	-
JAMCOH010000099.1	Ferric reductase		-	-
JAMCOH010000099.1	hypothetical protein		K08139	-
JAMCOH010000099.1	hypothetical protein		K18849	-
JAMCOH010000099.1	hypothetical protein	COG0513	K14805	-
JAMCOH010000099.1	Eukaryotic peptide chain release fa	COG1503	K03265	-
JAMCOH010000099.1	hypothetical protein	COG5035	-	-
JAMCOH010000099.1	hypothetical protein	COG2130	K07119	-
JAMCOH010000099.1	hypothetical protein		K06683	-
JAMCOH010000099.1	Ferric reductase (1.6.99.14)		-	-
JAMCOH010000099.1	hypothetical protein	COG5119	K12260	-
JAMCOH010000099.1	hypothetical protein		K06664	-
JAMCOH010000099.1	hypothetical protein		K14844	-
JAMCOH010000099.1	hypothetical protein		K00860	-
JAMCOH010000099.1	Thymidylate synthase (EC 2.1.1.45)	COG0207	K00560	-
JAMCOH010000099.1	Phosphoserine aminotransferase (E	COG1932	K00831	-
JAMCOH010000099.1	hypothetical protein		K02544	-
JAMCOH010000099.1	Cleavage and polyadenylation endo	COG1236	K14403	-
JAMCOH010000099.1	hypothetical protein	COG1958	K11096	-
JAMCOH010000099.1	DNA replication licensing factor MC	COG1241	K02209	-
JAMCOH010000099.1	hypothetical protein		K14841	-
JAMCOH010000099.1	High-affnity carbon uptake protein Hat/HatR		K14299	-
JAMCOH010000099.1	UDP-N-acetylglucosamine--dolichyl	COG0472	K01001	-
JAMCOH010000099.1	hypothetical protein		K19475	-
JAMCOH010000099.1	hypothetical protein		K19475	-
JAMCOH010000099.1	Diphthine methyltransferase (EC 3.1.1.97)		K17868	-
JAMCOH010000099.1	NAD-specific glutamate dehydroge	COG0334	K15371	-
JAMCOH010000099.1	Ribose-phosphate pyrophosphokin	COG0462	K00948	-

JAMCOH010000099.1	hypothetical protein		K11826	-
JAMCOH010000099.1	hypothetical protein		K18667	-
JAMCOH010000099.1	hypothetical protein		K10082	-
JAMCOH010000099.1	hypothetical protein		K17663	-
JAMCOH010000099.1	putative phosphoglycerate mutase	COG0588	K22315	-
JAMCOH010000099.1	Inosine-uridine preferring nucleosid	COG1957	K01240	-
JAMCOH010000099.1	hypothetical protein	COG0596	-	-
JAMCOH010000099.1	RNA terminal phosphate cyclase-lik	COG0430	K11108	-
JAMCOH010000099.1	hypothetical protein	COG5098	K06677	-
JAMCOH010000099.1	hypothetical protein	COG5202	K13519	-
JAMCOH010000099.1	Ferrochelatase, protoheme ferro-ly	COG0276	K01772	-
JAMCOH010000099.1	hypothetical protein		K15146	-
JAMCOH010000099.1	NAD-dependent aldehyde dehydro	COG1012	-	-
JAMCOH010000099.1	hypothetical protein		K02152	-
JAMCOH010000099.1	hypothetical protein	COG1275	-	-
JAMCOH010000099.1	Ribonucleotide reductase of class Ia	COG1678	K10808	-
JAMCOH010000099.1	hypothetical protein	COG0553	K11681	-
JAMCOH010000099.1	hypothetical protein	COG0553	K11681	-
JAMCOH010000099.1	hypothetical protein		K03964	-
JAMCOH010000099.1	protein serine/threonine kinase activity		K09490,K11835	-
JAMCOH010000099.1	hypothetical protein		K06641,K08286,K08794,K12776	-
JAMCOH010000099.1	hypothetical protein		K11267	-
JAMCOH010000099.1	hypothetical protein		K18723	-
JAMCOH010000099.1	hypothetical protein	COG0094	K02931	-
JAMCOH010000099.1	hypothetical protein		K14798	-
JAMCOH010000099.1	GTP-binding protein RHO2	COG1100	K07975	-
JAMCOH010000099.1	hypothetical protein	COG0697	-	-
JAMCOH010000099.1	Actin-related protein 3	COG5277	K18584	-
JAMCOH010000099.1	hypothetical protein	COG5032	K07203	-
JAMCOH010000099.1	hypothetical protein		K14771	-
JAMCOH010000099.1	DNA replication licensing factor MC	COG1241	K02541	-
JAMCOH010000099.1	hypothetical protein		K14292	-
JAMCOH010000099.1	Chaperone protein DnaK	COG0443	K04043	-
JAMCOH010000099.1	hypothetical protein		K01381	-
JAMCOH010000099.1	hypothetical protein	COG0429	K07019	-
JAMCOH010000099.1	Thermosome subunit	COG0459	K09497	-
JAMCOH010000099.1	DNA-directed RNA polymerase I 13	COG1594	K03000	-
JAMCOH010000099.1	Deoxyhypusine hydroxylase (EC 1.1	COG1413	K06072	-
JAMCOH010000099.1	O-acetyl-ADP-ribose deacetylase	COG2110	-	-
JAMCOH010000099.1	Nitrilotriacetate monooxygenase co	COG1853	-	-
JAMCOH010000099.1	3-dehydroquinate dehydratase II (E	COG0757	K03786	-
JAMCOH010000099.1	Catechol 1,2-dioxygenase 1 (EC 1.1	COG3485	-	-
JAMCOH010000099.1	Succinyl-CoA:3-ketoacid-coenzyme	COG2057	K01027	-
JAMCOH010000099.1	hypothetical protein		K11876,K11880	-
JAMCOH010000099.1	hypothetical protein		K04345	-
JAMCOH010000099.1	DNA-directed RNA polymerase II 13	COG1594	K03017	-
JAMCOH010000099.1	LSU ribosomal protein L7/L12 (L23e	COG0222	K02935	-
JAMCOH010000099.1	Manganese transport protein MntH	COG1914	K12346	-
JAMCOH010000099.1	Manganese transport protein MntH	COG1914	K12346	-
JAMCOH010000099.1	2-hydroxy-3-keto-5-methylthiopent	COG4359	-	-
JAMCOH010000099.1	Serine/threonine protein kinase		K08286	-
JAMCOH010000099.1	hypothetical protein	COG0553	K15710	-
JAMCOH010000099.1	hypothetical protein	COG5235	K10739	-
JAMCOH010000099.1	hypothetical protein	COG5531	K06276,K11650,K11760,K11775	-
JAMCOH010000100.1	hypothetical protein		K14022	-
JAMCOH010000100.1	hypothetical protein		K10751	-
JAMCOH010000100.1	hypothetical protein	COG1362	K01268	-
JAMCOH010000100.1	Chaperone protein DnaJ	COG0484	K03686	-
JAMCOH010000100.1	Beta-hexosaminidase (EC 3.2.1.52)	COG3525	K12373	GH20
JAMCOH010000100.1	Dimethylallyltransferase (EC 2.5.1.1	COG0142	K00804	-

JAMCOH010000101.1	Protein MSP1	COG0464	K01509	-
JAMCOH010000101.1	Ulp1 protease family, C-terminal catalytic domain	COG5160	K08592	-
JAMCOH010000102.1	Glutamate decarboxylase (EC 4.1.1.	COG0076	K01580	-
JAMCOH010000102.1	hypothetical protein		K01527	-
JAMCOH010000102.1	Diacylglycerol pyrophosphate phos	COG0671	K18693	-
JAMCOH010000102.1	hypothetical protein		K11243	-
JAMCOH010000102.1	Homoserine O-acetyltransferase (E	COG2021	K00641	-
JAMCOH010000102.1	SVP26 protein associated with early Golgi proteins (SN		-	-
JAMCOH010000102.1	Phospholipase	COG1502	K01115	-
JAMCOH010000102.1	hypothetical protein	COG3805	K10253	-
JAMCOH010000102.1	hypothetical protein	COG0218	K03978	-
JAMCOH010000102.1	H/ACA ribonucleoprotein complex s	COG3277	K11128	-
JAMCOH010000102.1	hypothetical protein		K12472	-
JAMCOH010000102.1	hypothetical protein	COG2175	K03119,K19245	-
JAMCOH010000102.1	Lead, cadmium, zinc and mercury t	COG2217	-	-
JAMCOH010000102.1	Serine/threonine protein phosphat	COG0631	K19704	-
JAMCOH010000102.1	Chaperone protein DnaJ	COG0484	-	-
JAMCOH010000102.1	hypothetical protein		K12481	-
JAMCOH010000102.1	hypothetical protein	COG1524	K05285	-
JAMCOH010000102.1	Indole-3-glycerol phosphate syntha	COG0134	K01656,K13501	-
JAMCOH010000102.1	hypothetical protein		K08158	-
JAMCOH010000102.1	hypothetical protein	COG1474	K02213	-
JAMCOH010000102.1	SSU ribosomal protein S9p (S16e)	COG0103	K02996	-
JAMCOH010000102.1	Alanyl-tRNA synthetase family prot	COG2872	K01872,K07050	-
JAMCOH010000102.1	hypothetical protein		K01383,K06005,K06009	-
JAMCOH010000102.1	Dehydrodolichyl diphosphate synth	COG0020	K11778	-
JAMCOH010000102.1	hypothetical protein	COG0020	K00293,K11778	-
JAMCOH010000102.1	hypothetical protein	COG0517	-	-
JAMCOH010000102.1	hypothetical protein	COG0705	K20028	-
JAMCOH010000102.1	hypothetical protein	COG5154	K14820	-
JAMCOH010000102.1	hypothetical protein		K02143	-
JAMCOH010000102.1	Exosome complex exonuclease DIS3	COG0557	K12585	-
JAMCOH010000102.1	3,4-dihydroxy-2-butanone 4-phosp	COG0807	K02858	-
JAMCOH010000102.1	hypothetical protein		K11881	-
JAMCOH010000102.1	Tryptophanyl-tRNA synthetase (EC	COG0180	K01867	-
JAMCOH010000102.1	LSU ribosomal protein L27e	COG2163	K02901	-
JAMCOH010000102.1	Protein kinase		K06276	-
JAMCOH010000103.1	DNA mismatch repair protein MutL	COG0323	K10858	-
JAMCOH010000103.1	hypothetical protein	COG1132	-	-
JAMCOH010000104.1	hypothetical protein	COG0501	K06013	-
JAMCOH010000104.1	hypothetical protein	COG0656	K19654	-
JAMCOH010000104.1	Serine hydroxymethyltransferase (E	COG0112	K00600	-
JAMCOH010000104.1	hypothetical protein		K15160	-
JAMCOH010000105.1	hypothetical protein		K19845	-
JAMCOH010000106.1	hypothetical protein		K01078	-
JAMCOH010000106.1	hypothetical protein		K01098	-
JAMCOH010000107.1	hypothetical protein	COG1131	K08711,K08712	-
JAMCOH010000107.1	hypothetical protein		K11558	-
JAMCOH010000107.1	Sphingomyelinase (EC 3.1.4.12)		K12351	-
JAMCOH010000108.1	hypothetical protein		K15205	-
JAMCOH010000108.1	Sulfur carrier protein adenylyltrans	COG0476	K11996	-
JAMCOH010000108.1	hypothetical protein		K20346	-
JAMCOH010000108.1	hypothetical protein		K20346	-
JAMCOH010000108.1	Serine/threonine-protein kinase RI	COG1718	K07178	-
JAMCOH010000109.1	hypothetical protein	COG5032	K19801	-
JAMCOH010000109.1	hypothetical protein		K22285	-
JAMCOH010000109.1	poly(A) polymerase	COG5186	K14376	-
JAMCOH010000110.1	hypothetical protein		K22193	-
JAMCOH010000110.1	Glycyl-tRNA synthetase (EC 6.1.1.14	COG0423	K01880	-
JAMCOH010000110.1	Thimet oligopeptidase (EC 3.4.24.1	COG0339	K01405,K13726	-

JAMCOH010000111.1	hypothetical protein	COG1131	K08711,K08712	-
JAMCOH010000111.1	Thiazole biosynthetic enzyme Thi4	COG1635	K03146	-
JAMCOH010000111.1	tRNA (guanine(10)-N(2))-methyltra	COG1041	K15430	-
JAMCOH010000112.1	hypothetical protein	COG1132	-	-
JAMCOH010000113.1	tRNA dimethylallyltransferase (EC 2	COG0324	K00791	-
JAMCOH010000113.1	hypothetical protein		K12200	-
JAMCOH010000113.1	hypothetical protein	COG5147	K12860	-
JAMCOH010000114.1	hypothetical protein	COG0474	K01537	-
JAMCOH010000115.1	ATP-dependent RNA helicase CHL1	COG1199	K11273	-
JAMCOH010000115.1	L-serine dehydratase, (PLP)-depend	COG1171	K17989	-
JAMCOH010000116.1	Guanidinobutyrase (EC 3.5.3.7)	COG0010	K01480	-
JAMCOH010000116.1	Endo-1,3(4)-beta-glucanase (EC 3.2	COG5498	K01180	-
JAMCOH010000118.1	hypothetical protein		K03849	GT57
JAMCOH010000118.1	hypothetical protein		K18655	-
JAMCOH010000118.1	Iron/manganese superoxide dismut	COG0605	K04564	-
JAMCOH010000119.1	Galactose/methyl galactoside ABC transporter, substr		K19720	-
JAMCOH010000120.1	hypothetical protein		K03261	-
JAMCOH010000120.1	hypothetical protein		K21842	-
JAMCOH010000121.1	hypothetical protein	COG5432	K10627	-
JAMCOH010000121.1	Formate transporter	COG2116	K00122	-
JAMCOH010000122.1	hypothetical protein		K12345	-
JAMCOH010000122.1	hypothetical protein		K12184	-
JAMCOH010000122.1	hypothetical protein		K03031	-
JAMCOH010000122.1	hypothetical protein		K15902	-
JAMCOH010000122.1	hypothetical protein	COG0666	K06867	-
JAMCOH010000122.1	Serine/threonine protein kinase		K08286	-
JAMCOH010000122.1	hypothetical protein		K15448	-
JAMCOH010000122.1	DNA mismatch repair protein MutS	COG0249	K08736	-
JAMCOH010000122.1	hypothetical protein	COG0596	K13535	-
JAMCOH010000122.1	hypothetical protein		K11676	-
JAMCOH010000122.1	hypothetical protein	COG2256	K07478	-
JAMCOH010000122.1	Eukaryotic translation initiation factor 4G		K03260	-
JAMCOH010000122.1	proteasome regulatory subunit Rpt	COG0554	K03066	-
JAMCOH010000122.1	hypothetical protein	COG0484	K09523	-
JAMCOH010000122.1	Aminopeptidase Y (Arg, Lys, Leu pre	COG2234	K01264	-
JAMCOH010000122.1	Inorganic pyrophosphatase (EC 3.6.	COG0221	K01507	-
JAMCOH010000122.1	hypothetical protein	COG1697	K10878	-
JAMCOH010000122.1	Protein kinase		K20872	-
JAMCOH010000122.1	hypothetical protein		K09467	-
JAMCOH010000122.1	hypothetical protein	COG5063	-	-
JAMCOH010000122.1	ATP-dependent DNA helicase RecQ	COG0514	K10901	-
JAMCOH010000122.1	hypothetical protein	COG5078	K10688	-
JAMCOH010000123.1	Chaperone protein DnaJ	COG2214	-	-
JAMCOH010000123.1	hypothetical protein	COG0308	K01254,K15428	-
JAMCOH010000123.1	hypothetical protein	COG3805	K10253	-
JAMCOH010000123.1	hypothetical protein		K14017	-
JAMCOH010000124.1	Nuclear protein STH1/NPS1	COG0553	K11786	-
JAMCOH010000124.1	hypothetical protein		K17806	-
JAMCOH010000124.1	hypothetical protein		K06005	-
JAMCOH010000125.1	Chromosome partition protein smc	COG1196	K06675	-
JAMCOH010000125.1	tRNA-dihydrouridine(20a/20b) synt	COG0042	K05545	-
JAMCOH010000125.1	hypothetical protein	COG1864	K01173	-
JAMCOH010000126.1	hypothetical protein	COG1052	K00015	-
JAMCOH010000126.1	hypothetical protein		K17979	-
JAMCOH010000126.1	hypothetical protein	COG1104	K01763,K20247	-
JAMCOH010000126.1	hypothetical protein	COG1052	K00015	-
JAMCOH010000127.1	Carbamoyl-phosphate synthase larg	COG0458	K01955	-
JAMCOH010000128.1	hypothetical protein	COG1960	K00232	-
JAMCOH010000128.1	hypothetical protein	COG1960	K00232	-
JAMCOH010000128.1	hypothetical protein		K17772	-
JAMCOH010000128.1	hypothetical protein	COG5238	K14319	-

JAMCOH010000128.1	hypothetical protein	COG0419	K10866	-
JAMCOH010000128.1	hypothetical protein	COG0204	K13513,K13523	-
JAMCOH010000128.1	hypothetical protein		K14787	-
JAMCOH010000128.1	hypothetical protein	COG0705	K09650	-
JAMCOH010000128.1	hypothetical protein		K14817	-
JAMCOH010000128.1	Formamidase (EC 3.5.1.49)	COG2421	K01455	-
JAMCOH010000128.1	hypothetical protein	COG2175	K03119,K19245	-
JAMCOH010000128.1	hypothetical protein	COG5047	K14006	-
JAMCOH010000128.1	hypothetical protein		K19841	-
JAMCOH010000128.1	LSU ribosomal protein L12e (L11p)	COG0080	K02870	-
JAMCOH010000128.1	hypothetical protein	COG4281	-	-
JAMCOH010000128.1	hypothetical protein		K14548	-
JAMCOH010000128.1	Sterol 24-C-methyltransferase (EC 2	COG0500	K00559	-
JAMCOH010000128.1	hypothetical protein	COG5218	K06678	-
JAMCOH010000128.1	hypothetical protein		K09043	-
JAMCOH010000128.1	hypothetical protein		K20823	-
JAMCOH010000128.1	Deoxyhypusine synthase (EC 2.5.1.4	COG1899	K00809	-
JAMCOH010000128.1	Vacuolar aminopeptidase I (EC 3.4.1	COG1362	K01268	-
JAMCOH010000128.1	hypothetical protein		K04630,K04640	-
JAMCOH010000128.1	hypothetical protein		K00916	-
JAMCOH010000128.1	Glutamine--fructose-6-phosphate a	COG0449	K00820	-
JAMCOH010000128.1	hypothetical protein	COG1723	-	-
JAMCOH010000129.1	hypothetical protein	COG5271	K07763,K14572,K14766,K17046	-
JAMCOH010000130.1	ATP synthase gamma chain (EC 3.6.	COG0224	K02136	-
JAMCOH010000132.1	hypothetical protein	COG2801	-	-
JAMCOH010000134.1	hypothetical protein		K19720	-
JAMCOH010000135.1	hypothetical protein	COG2801	-	-
JAMCOH010000136.1	hypothetical protein		K05857	-
JAMCOH010000136.1	hypothetical protein	COG5210	K20168	-
JAMCOH010000138.1	hypothetical protein		K22493	-
JAMCOH010000139.1	Sulfite reductase [NADPH] flavopro	COG0369	K00380	-
JAMCOH010000141.1	hypothetical protein	COG2801	K07497	-
JAMCOH010000142.1	hypothetical protein		K22493	-
JAMCOH010000143.1	Alkaline phosphatase (EC 3.1.3.1)	COG1785	K01077	-
JAMCOH010000144.1	hypothetical protein	COG1132	-	-
JAMCOH010000145.1	hypothetical protein		K14309	-
JAMCOH010000146.1	NAD-dependent protein deacetylas	COG0846	K11121	-
JAMCOH010000146.1	Amino acid permease	COG0531	K19564	-
JAMCOH010000148.1	Superoxide dismutase [Cu-Zn] prec	COG2032	K04565	-
JAMCOH010000149.1	Cytochrome c peroxidase (EC 1.11.1	COG0685	K00428	-
JAMCOH010000149.1	Nuclear protein STH1/NPS1	COG0553	K11786	-
JAMCOH010000150.1	hypothetical protein		K15216	-
JAMCOH010000150.1	Steryl acetyl hydrolase	COG0657	-	-
JAMCOH010000151.1	hypothetical protein	COG5640	-	-
JAMCOH010000151.1	hypothetical protein	COG5200	-	-
JAMCOH010000151.1	hypothetical protein	COG0564	K15265	-
JAMCOH010000151.1	hypothetical protein		K06005	-
JAMCOH010000151.1	hypothetical protein		K14709	-
JAMCOH010000151.1	Methylated-DNA--protein-cysteine	COG0350	K00567	-
JAMCOH010000151.1	hypothetical protein		K14686	-
JAMCOH010000151.1	Phosphoadenylyl-sulfate reductase	COG0175	K00390	-
JAMCOH010000151.1	hypothetical protein	COG2124	K00493	-
JAMCOH010000151.1	hypothetical protein		K04711,K05848	-
JAMCOH010000151.1	DNA mismatch repair protein MutS	COG0249	K08735,K19476	-
JAMCOH010000151.1	hypothetical protein	COG1131	-	-
JAMCOH010000151.1	Arsenical-resistance protein ACR3	COG0798	K03325	-
JAMCOH010000151.1	hypothetical protein		K10881	-
JAMCOH010000151.1	proteasome regulatory subunit Rpt	COG1222	K03065	-
JAMCOH010000151.1	Cytidine deaminase (EC 3.5.4.5)	COG0295	K01489	-
JAMCOH010000151.1	Methionine aminopeptidase (EC 3.4	COG0024	K01265	-
JAMCOH010000151.1	hypothetical protein	COG2178	-	-

JAMCOH010000151.1	hypothetical protein	COG0459	K09498	-
JAMCOH010000151.1	hypothetical protein		K21633	-
JAMCOH010000151.1	Hydrophilic suppressor of ypt1 invo	COG5158	K19998	-
JAMCOH010000151.1	RuvB-like DNA helicase RVB1 implic	COG1224	K04499	-
JAMCOH010000151.1	GPN-loop GTPase 3		K06883	-
JAMCOH010000151.1	hypothetical protein	COG1230	K14692	-
JAMCOH010000151.1	Glycerol-3-phosphate dehydrogena	COG0578	K00111	-
JAMCOH010000151.1	hypothetical protein		K14013	-
JAMCOH010000151.1	hypothetical protein	COG0589	-	-
JAMCOH010000151.1	Box C/D RNA-guided RNA methyltra	COG1889	K14563	-
JAMCOH010000151.1	hypothetical protein		K11599	-
JAMCOH010000151.1	hypothetical protein		K18730	-
JAMCOH010000151.1	hypothetical protein		K18730	-
JAMCOH010000151.1	hypothetical protein	COG5391	K17917	-
JAMCOH010000155.1	Sulfate permease and related trans	COG0659	K03321	-
JAMCOH010000156.1	Glycosyl hydrolase		-	-
JAMCOH010000157.1	Cobalt-zinc-cadmium resistance pro	COG1230	K14688	-
JAMCOH010000157.1	hypothetical protein		K18759	-
JAMCOH010000158.1	Glutathione S-transferase (EC 2.5.1.	COG0625	K00799	-
JAMCOH010000159.1	hypothetical protein		K21016	-
JAMCOH010000161.1	NAD-dependent formate dehydrog	COG1052	K00122	-
JAMCOH010000161.1	hypothetical protein	COG1052	K00122	-
JAMCOH010000162.1	D-3-phosphoglycerate dehydrogena	COG1052	K00122	-
JAMCOH010000162.1	NAD-dependent formate dehydrog	COG1052	K00122	-
JAMCOH010000164.1	Pyruvate decarboxylase (EC 4.1.1.1)	COG3961	K04103	-
JAMCOH010000164.1	Pyruvate decarboxylase (EC 4.1.1.1)	COG3961	K01568	-
JAMCOH010000165.1	Aconitate hydratase (EC 4.2.1.3)	COG1048	K01681	-
JAMCOH010000167.1	hypothetical protein	COG2365	K18046	-
JAMCOH010000167.1	hypothetical protein		K11839	-
JAMCOH010000168.1	hypothetical protein	COG1028	K00218	-
JAMCOH010000169.1	hypothetical protein		K19476	-
JAMCOH010000169.1	hypothetical protein	COG1225	K03564	-
JAMCOH010000169.1	hypothetical protein	COG5027	K11304	-
JAMCOH010000169.1	hypothetical protein		K11723,K12862	-
JAMCOH010000169.1	hypothetical protein		K14137	-
JAMCOH010000169.1	hypothetical protein		K10579	-
JAMCOH010000169.1	High-affnity carbon uptake protein Hat/HatR		K16794	-
JAMCOH010000169.1	ATP synthase delta chain (EC 3.6.3.1	COG0712	K02137	-
JAMCOH010000169.1	LSU ribosomal protein L2p (L8e)	COG0090	-	-
JAMCOH010000169.1	hypothetical protein		K11133	-
JAMCOH010000169.1	Ribonuclease Z (EC 3.1.26.11)	COG1234	K00784	-
JAMCOH010000169.1	hypothetical protein	COG1752	K14674	-
JAMCOH010000169.1	hypothetical protein		K14715	-
JAMCOH010000169.1	hypothetical protein		K15068	-
JAMCOH010000169.1	hypothetical protein		K14770	-
JAMCOH010000169.1	hypothetical protein	COG1859	K10669	-
JAMCOH010000169.1	hypothetical protein		K18170	-
JAMCOH010000169.1	hypothetical protein		K14547	-
JAMCOH010000169.1	hypothetical protein	COG0637	K06116,K06117	-
JAMCOH010000169.1	hypothetical protein		K10666	-
JAMCOH010000169.1	proteasome regulatory subunit Rpt	COG0554	K03062	-
JAMCOH010000169.1	hypothetical protein	COG5160	K08596	-
JAMCOH010000169.1	hypothetical protein	COG5210	-	-
JAMCOH010000169.1	Kynurenine 3-monooxygenase (EC 1	COG0654	K00486	-
JAMCOH010000169.1	hypothetical protein		K17804	-
JAMCOH010000169.1	hypothetical protein	COG5347	K12493	-
JAMCOH010000169.1	hypothetical protein	COG0604	K07512	-
JAMCOH010000169.1	Allantoinase (EC 3.5.2.5)	COG0044	K01466	-
JAMCOH010000169.1	hypothetical protein	COG5264	-	-
JAMCOH010000169.1	hypothetical protein	COG5180	-	-
JAMCOH010000169.1	hypothetical protein	COG5173	K06110	-

JAMCOH010000169.1	hypothetical protein	COG5134	-	-
JAMCOH010000169.1	SSU ribosomal protein S24e	COG2004	K02974	-
JAMCOH010000169.1	UPF0743 protein YCR087C-A		K15263	-
JAMCOH010000170.1	hypothetical protein		K19719,K19721	-
JAMCOH010000171.1	hypothetical protein		K22493	-
JAMCOH010000172.1	Thiamin-phosphate pyrophosphory	COG2145	K14154	-
JAMCOH010000173.1	Protease III precursor (EC 3.4.24.55	COG1025	K01408	-
JAMCOH010000174.1	hypothetical protein	COG0098	K02988	-
JAMCOH010000175.1	hypothetical protein		K18191	-
JAMCOH010000176.1	Aminotransferase class I and II	COG0436	-	-
JAMCOH010000177.1	Putative sodium:solute symporter,	COG0591	K20989	-
JAMCOH010000178.1	hypothetical protein	COG0496	K03787	-
JAMCOH010000180.1	hypothetical protein	COG1131	K08711,K08712	-
JAMCOH010000181.1	Guanidinobutyrase (EC 3.5.3.7)	COG0010	K01480	-
JAMCOH010000183.1	Aspartyl aminopeptidase (EC 3.4.11	COG1362	K01267	-
JAMCOH010000183.1	hypothetical protein	COG2340	-	-
JAMCOH010000186.1	hypothetical protein		K10273	-
JAMCOH010000187.1	hypothetical protein		K21436	-
JAMCOH010000188.1	hypothetical protein	COG1132	-	-
JAMCOH010000188.1	tRNA (carboxymethyluridine(34)-5-	COG0500	K15444	-
JAMCOH010000188.1	hypothetical protein		K18203	-
JAMCOH010000190.1	hypothetical protein	COG2801	-	-
JAMCOH010000191.1	Endonuclease III (EC 4.2.99.18)	COG0177	K10773	-
JAMCOH010000192.1	Cytosine/purine/uracil/thiamine/al	COG1953	K03457	-
JAMCOH010000193.1	hypothetical protein		K22494	-
JAMCOH010000196.1	hypothetical protein	COG2801	-	-
JAMCOH010000198.1	hypothetical protein	COG0672	K07243	-
JAMCOH010000202.1	Dinucleoside triphosphate hydrolase	COG0537	K01522	-
JAMCOH010000202.1	Alkyl hydroperoxide reductase subu	COG0450	K03386	-
JAMCOH010000204.1	hypothetical protein		K14801	-
JAMCOH010000204.1	hypothetical protein	COG0474	K01536	-
JAMCOH010000206.1	hypothetical protein	COG1052	K00015	-
JAMCOH010000206.1	Glucan 1,3-beta-glucosidase (EC 3.2	COG2730	K01210	-
JAMCOH010000206.1	LSU ribosomal protein P1 (L7/L12)	COG2058	K02942	-
JAMCOH010000206.1	LSU ribosomal protein L13e	COG4352	K02873	-
JAMCOH010000206.1	SSU ribosomal protein S16e (S9p)	COG0088	K02960	-
JAMCOH010000206.1	hypothetical protein		K14800	-
JAMCOH010000206.1	Origin recognition complex subunit	COG1474	K02603	-
JAMCOH010000206.1	Protein TEM1	COG1100	K06682	-
JAMCOH010000206.1	SSU ribosomal protein S3Ae	COG1890	K02984	-
JAMCOH010000206.1	hypothetical protein	COG5038	-	-
JAMCOH010000206.1	LSU ribosomal protein L6e	COG2163	K02934	-
JAMCOH010000206.1	Peptidyl-prolyl cis-trans isomerase (	COG0545	K14826	-
JAMCOH010000206.1	Hydroxymethylglutaryl-CoA reducta	COG1257	K00021	-
JAMCOH010000206.1	Cystathionine gamma-synthase (EC	COG0626	K01739	-
JAMCOH010000206.1	hypothetical protein		K01230	GH47
JAMCOH010000206.1	hypothetical protein		K09051	-
JAMCOH010000206.1	hypothetical protein		K00624	-
JAMCOH010000206.1	Gamma-glutamyltranspeptidase (E(	COG0405	K00681,K18592	-
JAMCOH010000206.1	hypothetical protein	COG5017	K07432	GT1
JAMCOH010000206.1	hypothetical protein		K01117	-
JAMCOH010000207.1	Low-specificity L-threonine aldolase	COG2008	K01620	-
JAMCOH010000210.1	UPF0061 protein YdiU	COG0397	-	-
JAMCOH010000211.1	hypothetical protein	COG5271	K07763,K14572,K14766,K17046	-
JAMCOH010000214.1	hypothetical protein	COG0477	-	-
JAMCOH010000214.1	hypothetical protein		K01771	-
JAMCOH010000214.1	hypothetical protein		K17983	-
JAMCOH010000214.1	proteasome regulatory subunit Rpt	COG1222	K03063	-
JAMCOH010000214.1	hypothetical protein		K08098	GT22
JAMCOH010000214.1	Isocitrate dehydrogenase [NAD] sul	COG0473	K00030	-

JAMCOH010000214.1	Phosphoribosylglycinamide formylt	COG0299	K00601	-
JAMCOH010000214.1	Translation elongation factor 2	COG0480	K03234	-
JAMCOH010000214.1	Monoamine oxidase (1.4.3.4)	COG3733	K00276	-
JAMCOH010000214.1	Ribonucleotide reductase of class Ia	COG0209	K10807	-
JAMCOH010000214.1	hypothetical protein	COG5021	K10592	-
JAMCOH010000214.1	hypothetical protein		K12843	-
JAMCOH010000214.1	hypothetical protein		K02143	-
JAMCOH010000214.1	hypothetical protein	COG2428	-	-
JAMCOH010000214.1	hypothetical protein	COG0664	K04739	-
JAMCOH010000214.1	Chaperone protein DnaK	COG0443	K09490	-
JAMCOH010000214.1	hypothetical protein	COG5665	K12580	-
JAMCOH010000214.1	hypothetical protein		K06653,K13345	-
JAMCOH010000215.1	hypothetical protein		K08334	-
JAMCOH010000215.1	Enoyl-(Acyl carrier protein) reductase	COG1028	K00218	-
JAMCOH010000215.1	tRNA-splicing endonuclease subuni	COG1676	K15322	-
JAMCOH010000215.1	hypothetical protein	COG5247	K21752	-
JAMCOH010000215.1	D-ribulokinase (EC 2.7.1.47)	COG0554	-	-
JAMCOH010000215.1	sulfate permease	COG0659	K14708	-
JAMCOH010000215.1	hypothetical protein		K10750	-
JAMCOH010000215.1	hypothetical protein		K15079	-
JAMCOH010000215.1	Pyruvate carboxylase (EC 6.4.1.1)	COG1038	K01958	-
JAMCOH010000215.1	hypothetical protein		K01640,K09522	-
JAMCOH010000215.1	hypothetical protein	COG1500	K14574	-
JAMCOH010000215.1	hypothetical protein		K00419	-
JAMCOH010000215.1	hypothetical protein	COG2273	K01216	-
JAMCOH010000215.1	hypothetical protein		K01383,K06005,K06009	-
JAMCOH010000215.1	Ferric reductase (1.6.99.14)		-	-
JAMCOH010000215.1	Cytochrome oxidase biogenesis pro	COG3175	K02258	-
JAMCOH010000215.1	hypothetical protein		K19985	-
JAMCOH010000215.1	hypothetical protein	COG3194	K01483	-
JAMCOH010000215.1	hypothetical protein	COG0699	K06030	-
JAMCOH010000215.1	hypothetical protein		K03122	-
JAMCOH010000215.1	hypothetical protein		K14000,K17560,K20478	-
JAMCOH010000215.1	hypothetical protein	COG0624	K01293	-
JAMCOH010000215.1	DNA-directed RNA polymerases I, II	COG1644	K03007	-
JAMCOH010000215.1	hypothetical protein		K14552	-
JAMCOH010000215.1	hypothetical protein	COG0699	K22140	-
JAMCOH010000216.1	hypothetical protein		K11399	-
JAMCOH010000216.1	SSU ribosomal protein S4p (S9e), m	COG0522	-	-
JAMCOH010000216.1	Peptide chain release factor N(5)-gl	COG2890	-	-
JAMCOH010000216.1	hypothetical protein		K03256	-
JAMCOH010000216.1	25S rRNA (cytosine(2870)-C(5))-met	COG0144	K14835	-
JAMCOH010000216.1	Actin-related protein 5		K11672	-
JAMCOH010000216.1	hypothetical protein		K17261	-
JAMCOH010000216.1	Chaperone protein DnaJ	COG0484	K09503	-
JAMCOH010000216.1	Phosphoribulokinase	COG0572	K10524	-
JAMCOH010000216.1	tRNA cytosine(12) acetyltransferase	COG1444	K14521	-
JAMCOH010000216.1	hypothetical protein		K12769	-
JAMCOH010000216.1	hypothetical protein	COG1958	K12621	-
JAMCOH010000216.1	hypothetical protein		K06316	-
JAMCOH010000216.1	hypothetical protein	COG2036	K08065	-
JAMCOH010000216.1	Aminopeptidase C (EC 3.4.22.40)	COG3579	K01372	-
JAMCOH010000216.1	hypothetical protein	COG1075	K01074	-
JAMCOH010000216.1	Multisite-specific tRNA:(cytosine-C(	COG0144	K15334	-
JAMCOH010000216.1	hypothetical protein		K15221	-
JAMCOH010000216.1	hypothetical protein	COG5096	K17301	-
JAMCOH010000216.1	H/ACA ribonucleoprotein complex s	COG1358	K11129	-
JAMCOH010000216.1	hypothetical protein		K11979,K16261	-
JAMCOH010000216.1	ATP-dependent protease La (EC 3.4	COG0466	K08675	-
JAMCOH010000216.1	Flagellar L-ring protein FlgH		-	-

JAMCOH010000216.1	Transcription initiation factor IID 90	COG2319	K03130	-
JAMCOH010000216.1	hypothetical protein		K01266,K03859	-
JAMCOH010000216.1	2-Oxobutyrate oxidase, putative	COG3491	-	-
JAMCOH010000216.1	Hexokinase (EC 2.7.1.1)	COG5026	K00844	-
JAMCOH010000216.1	hypothetical protein	COG2023	K03540	-
JAMCOH010000217.1	hypothetical protein		K22494	-
JAMCOH010000217.1	hypothetical protein		K05754	-
JAMCOH010000217.1	SAM-dependent methyltransferase	COG1565	K00231,K18164	-
JAMCOH010000217.1	hypothetical protein		K10080	-
JAMCOH010000217.1	Oxidoreductase, short-chain dehyd	COG1028	K17742	-
JAMCOH010000217.1	Hexokinase (EC 2.7.1.1)	COG5026	K00844	-
JAMCOH010000217.1	hypothetical protein		K21543	-
JAMCOH010000217.1	Carbamoyl-phosphate synthase larg	COG0458	K01954,K11541	-
JAMCOH010000217.1	hypothetical protein	COG0454	-	-
JAMCOH010000217.1	hypothetical protein		K14795	-
JAMCOH010000217.1	hypothetical protein	COG0607	-	-
JAMCOH010000217.1	hypothetical protein		K08267	-
JAMCOH010000217.1	hypothetical protein	COG5137	K10753	-
JAMCOH010000217.1	hypothetical protein	COG0679	K07088	-
JAMCOH010000218.1	hypothetical protein		K11843	-
JAMCOH010000218.1	hypothetical protein		K17788	-
JAMCOH010000218.1	Beta-lactamase superfamily domain	COG2220	K13985	-
JAMCOH010000218.1	GCN2 activating, bis-ABC ATPase GC	COG0488	K06158	-
JAMCOH010000218.1	Glutaredoxin	COG0695	K03676	-
JAMCOH010000218.1	Lactoylglutathione lyase (EC 4.4.1.5	COG0346	K01759	-
JAMCOH010000218.1	3-methyl-2-oxobutanoate hydroxym	COG0413	K00606	-
JAMCOH010000218.1	Phosphoglycerate mutase family	COG0588	K15634	-
JAMCOH010000218.1	hypothetical protein	COG0474	K01536	-
JAMCOH010000218.1	N-acetyltransferase B complex (NatB) non catalytic subunit		K17973	-
JAMCOH010000218.1	hypothetical protein	COG2730	-	-
JAMCOH010000218.1	hypothetical protein	COG5398	-	-
JAMCOH010000218.1	AMP/CMP kinase AK6	COG1936	K18532	-
JAMCOH010000218.1	hypothetical protein	COG5601	K12605	-
JAMCOH010000218.1	Belongs to the VPS29 family	COG0622	K07095,K18467	-
JAMCOH010000218.1	hypothetical protein		K02181	-
JAMCOH010000218.1	LSU ribosomal protein L13Ae (L13p)	COG0102	K02872	-
JAMCOH010000218.1	2-isopropylmalate synthase (EC 2.3	COG0119	K01649	-
JAMCOH010000218.1	hypothetical protein		K11294	-
JAMCOH010000218.1	hypothetical protein		K20063	-
JAMCOH010000218.1	Vps53-like, N-terminal		K20299	-
JAMCOH010000218.1	Phosphoribosylanthranilate isomer	COG0135	K01817	-
JAMCOH010000218.1	hypothetical protein		K09500	-
JAMCOH010000218.1	hypothetical protein	COG0558	K08744	-
JAMCOH010000218.1	hypothetical protein		K04450,K09047	-
JAMCOH010000218.1	Diphosphomevalonate decarboxyla	COG3407	K01597	-
JAMCOH010000218.1	FOG: WD40 repeat		K06666	-
JAMCOH010000219.1	hypothetical protein	COG5593	K14832	-
JAMCOH010000219.1	hypothetical protein	COG1100	K07827	-
JAMCOH010000219.1	hypothetical protein		K02677,K18050	-
JAMCOH010000219.1	SEH-associated protein 4		K20407	-
JAMCOH010000219.1	Streptococcal hemagglutinin protein		-	-
JAMCOH010000219.1	hypothetical protein	COG3673	-	-
JAMCOH010000219.1	Ubiquinol-cytochrome C reductase	COG0723	K00411	-
JAMCOH010000219.1	hypothetical protein	COG5021	K12232	-
JAMCOH010000219.1	hypothetical protein		K14304	-
JAMCOH010000219.1	V-type ATP synthase subunit K (EC 3	COG0636	K02155	-
JAMCOH010000219.1	LSU ribosomal protein L7Ae	COG1358	K12845	-

JAMCOH010000219.1	Urb2/Npa2 family		K14862	-
JAMCOH010000219.1	chloride channel	COG0038	K05012	-
JAMCOH010000219.1	Alcohol acetyltransferase		-	-
JAMCOH010000219.1	Syntaxin-like protein	COG5325	K21155	-
JAMCOH010000219.1	DNA polymerase alpha catalytic sub	COG0417	K02320	-
JAMCOH010000219.1	SNF5 / SMARCB1 / INI1		K11648	-
JAMCOH010000219.1	3-oxoacyl-[acyl-carrier protein] redu	COG1028	K06123	-
JAMCOH010000219.1	hypothetical protein		K15100	-
JAMCOH010000219.1	hypothetical protein		K11244	-
JAMCOH010000219.1	ATP-dependent helicase HrpB	COG1643	K12813,K12814	-
JAMCOH010000219.1	Uridine kinase, type 2 (EC 2.7.1.48)	COG0572	K00876	-
JAMCOH010000220.1	Intracellular protease	COG0693	K22211	-
JAMCOH010000220.1	Acyl-CoA:1-acyl-sn-glycerol-3-phosp	COG0204	K13509	-
JAMCOH010000220.1	hypothetical protein	COG4354	K12396	-
JAMCOH010000220.1	hypothetical protein		K22243	-
JAMCOH010000220.1	hypothetical protein		K06663	-
JAMCOH010000220.1	Hydantoin racemase (EC 5.1.99.-)	COG4126	-	-
JAMCOH010000220.1	hypothetical protein	COG5142	-	-
JAMCOH010000220.1	hypothetical protein	COG2072	-	-
JAMCOH010000220.1	Phosphoribosylformylglycinamidine synthase, synthet		K11870	-
JAMCOH010000220.1	hypothetical protein		K13201	-
JAMCOH010000220.1	tRNA(Phe) (4-demethylwyosine(37)	COG2520	K07055	-
JAMCOH010000220.1	hypothetical protein		K21543	-
JAMCOH010000220.1	hypothetical protein	COG4775	K07277	-
JAMCOH010000220.1	Phosphopantothenoylcysteine synt	COG0452	K01922,K11237	-
JAMCOH010000220.1	hypothetical protein	COG5333	K15161	-
JAMCOH010000220.1	hypothetical protein	COG5058	K04709	-
JAMCOH010000220.1	Kinesin-related protein	COG5059	K10401	-
JAMCOH010000220.1	Tetratricopeptide repeat family pro	COG0790	K07126	-
JAMCOH010000220.1	Belongs to the ubiquitin-conjugating enzyme family		K04554	-
JAMCOH010000220.1	Methyltransferase domain	COG2227	-	-
JAMCOH010000220.1	hypothetical protein		K02218	-
JAMCOH010000221.1	hypothetical protein		K17302	-
JAMCOH010000221.1	hypothetical protein	COG0438	K03844	GT4
JAMCOH010000221.1	Cytosine deaminase (EC 3.5.4.1)	COG0590	K01485	-
JAMCOH010000221.1	hypothetical protein	COG5242	K03143	-
JAMCOH010000221.1	SSU ribosomal protein S13e (S15p)	COG0184	K02953	-
JAMCOH010000221.1	Cofilin/tropomyosin-type actin-binding protein		-	-
JAMCOH010000221.1	hypothetical protein	COG2453	K06639	-
JAMCOH010000221.1	hypothetical protein		K20827	-
JAMCOH010000221.1	Heme A synthase, cytochrome oxid	COG1612	K02259	-
JAMCOH010000221.1	Phosphoglycerate kinase (EC 2.7.2.3	COG0126	K00927	-
JAMCOH010000221.1	hypothetical protein	COG0197	-	-
JAMCOH010000221.1	hypothetical protein		K10882	-
JAMCOH010000221.1	hypothetical protein		K01262,K06111	-
JAMCOH010000221.1	hypothetical protein		K14686	-
JAMCOH010000221.1	hypothetical protein		K01238	-
JAMCOH010000221.1	Lysine-specific permease	COG0531	K16261	-
JAMCOH010000222.1	hypothetical protein		K11293	-
JAMCOH010000222.1	hypothetical protein		K22384	-
JAMCOH010000222.1	hypothetical protein	COG5041	K03115	-
JAMCOH010000222.1	hypothetical protein	COG1076	K04082	-
JAMCOH010000222.1	hypothetical protein	COG5152	K13127,K20793	-
JAMCOH010000222.1	cytidylyltransferase	COG0615	K00967	-
JAMCOH010000222.1	Superoxide dismutase		K04565	-
JAMCOH010000222.1	Arginyl-tRNA--protein transferase (I	COG2935	K00685	-
JAMCOH010000222.1	Transcription initiation factor IIF be	COG5090	K03139	-
JAMCOH010000222.1	hypothetical protein		K02923	-
JAMCOH010000222.1	hypothetical protein		K12817	-

JAMCOH010000222.1	Uricase (urate oxidase) (EC 1.7.3.3)	COG3648	K00365	-
JAMCOH010000222.1	HesA/MoeB/ThiF family protein	COG1179	K22132	-
JAMCOH010000222.1	hypothetical protein		K20815	-
JAMCOH010000222.1	hypothetical protein		K15084	-
JAMCOH010000222.1	hypothetical protein		K12769	-
JAMCOH010000222.1	Flavin reductase	COG1853	-	-
JAMCOH010000222.1	hypothetical protein	COG5384	K14559	-
JAMCOH010000222.1	hypothetical protein	COG0553	K15505	-
JAMCOH010000222.1	Xaa-Pro aminopeptidase (EC 3.4.11	COG0006	K01262	-
JAMCOH010000222.1	hypothetical protein	COG1599	K07466	-
JAMCOH010000222.1	hypothetical protein	COG0626	K01760	-
JAMCOH010000222.1	NADH-ubiquinone oxidoreductase c	COG1905	K03943	-
JAMCOH010000222.1	ClpB protein	COG2319	K03364	-
JAMCOH010000222.1	hypothetical protein	COG5027	K11304,K11307,K11401	-
JAMCOH010000222.1	hypothetical protein		K02326	-
JAMCOH010000222.1	hypothetical protein	COG5143	K08515	-
JAMCOH010000222.1	RNA helicase, putative	COG1643	K14780	-
JAMCOH010000222.1	hypothetical protein		K18725	-
JAMCOH010000222.1	hypothetical protein	COG2154	K01724	-
JAMCOH010000222.1	Amino acid permease	COG0531	K19564	-
JAMCOH010000222.1	Mannan endo-1,6-alpha-mannosidase (EC 3.2.1.101)		K08257	GH76
JAMCOH010000222.1	Haloacid dehalogenase-like hydrolase	COG1011	K07025	-
JAMCOH010000222.1	Amino acid permease	COG0531	K19564	-
JAMCOH010000222.1	hypothetical protein		K11766	-
JAMCOH010000222.1	Manganese transport protein MntH	COG1914	K12346	-
JAMCOH010000222.1	hypothetical protein	COG0694	K22074	-
JAMCOH010000222.1	hypothetical protein		K02142	-
JAMCOH010000222.1	Lysine methyltransferase		-	-
JAMCOH010000222.1	Allantoicase (EC 3.5.3.4)	COG4266	K01477,K01483	-
JAMCOH010000222.1	Superoxide dismutases		K04565	-
JAMCOH010000222.1	Endonuclease/Exonuclease/phosp hatase family	COG3568	-	-
JAMCOH010000222.1	Cell division-associated, ATP-depen	COG0465	K08956	-
JAMCOH010000222.1	hypothetical protein		K11761	-
JAMCOH010000222.1	hypothetical protein	COG0596	K01052	-
JAMCOH010000222.1	hypothetical protein	COG2078	-	-
JAMCOH010000222.1	3-ketoacyl-CoA thiolase (EC 2.3.1.16	COG0183	K07513	-
JAMCOH010000222.1	hypothetical protein	COG0534	K03327	-
JAMCOH010000222.1	hypothetical protein	COG0170	K16368	-
JAMCOH010000222.1	[4Fe-4S] cluster assembly scaffold p	COG0489	K03593	-
JAMCOH010000222.1	tRNA-5-carboxymethylaminomethy	COG0486	K03650	-
JAMCOH010000222.1	hypothetical protein	COG5076	K11757,K11759	-
JAMCOH010000222.1	Hypothetical flavoprotein YqcA (clu	COG0369	-	-
JAMCOH010000222.1	Phenylalanyl-tRNA synthetase (EC 6	COG0016	K01889	-
JAMCOH010000222.1	hypothetical protein	COG4886	-	-
JAMCOH010000222.1	hypothetical protein	COG1902	K00354	-
JAMCOH010000222.1	Oxidoreductase	COG0673	-	-
JAMCOH010000223.1	Putative oxidoreductase	COG0667	K17647	-
JAMCOH010000223.1	L-2-aminoadipate reductase (EC 1.2	COG1020	K00143	-
JAMCOH010000223.1	hypothetical protein	COG0141	K15083	-
JAMCOH010000223.1	Eukaryotic translation initiation factor 3 39 kDa subun		K03246	-
JAMCOH010000223.1	Candidate 1: dienelactone hydrolas	COG0412	K01061	-
JAMCOH010000223.1	ATPase GET3, required for post-trar	COG0003	K01551	-
JAMCOH010000223.1	hypothetical protein	COG5076	K11756	-
JAMCOH010000223.1	NAD-dependent protein deacetylas	COG0846	K11415	-
JAMCOH010000223.1	Phenylalanyl-tRNA synthetase alpha	COG0016	K01889	-
JAMCOH010000223.1	UPF0057 membrane protein YqaE		-	-
JAMCOH010000223.1	hypothetical protein		K17770	-
JAMCOH010000223.1	Glucan 1,3-beta-glucosidase (EC 3.2	COG2730	K01210	-
JAMCOH010000223.1	galactosyl transferase		K05532	GT34

JAMCOH010000223.1	hypothetical protein		K00706	GT48
JAMCOH010000223.1	hypothetical protein	COG5309	K01210	-
JAMCOH010000224.1	Acetolactate synthase small subuni	COG0440	K01653	-
JAMCOH010000224.1	hypothetical protein	COG2036	-	-
JAMCOH010000224.1	hypothetical protein		K11237	-
JAMCOH010000224.1	Glycylpeptide N-tetradecanoyltrans	COG5092	K00671	-
JAMCOH010000224.1	hypothetical protein		K14791	-
JAMCOH010000224.1	hypothetical protein		K10295	-
JAMCOH010000224.1	hypothetical protein		K03252	-
JAMCOH010000224.1	n-type ATP pyrophosphatase super	COG0037	K14168	-
JAMCOH010000224.1	NADP-dependent 3-hydroxy acid de	COG1028	K16066	-
JAMCOH010000224.1	LSU ribosomal protein L18Ae	COG2157	K02882	-
JAMCOH010000224.1	Fatty acid hydroxylase family (carot	COG3000	K07750	-
JAMCOH010000224.1	Carbamoyl-phosphate synthase sm	COG0458	K01956	-
JAMCOH010000224.1	hypothetical protein	COG5222	K15541	-
JAMCOH010000224.1	hypothetical protein		K14303	-
JAMCOH010000224.1	hypothetical protein		K17606	-
JAMCOH010000224.1	Transcription initiation factor IIA, ga	COG5123	K03123	-
JAMCOH010000224.1	DUF89 domain of unknown function		-	-
JAMCOH010000225.1	Ribose-phosphate pyrophosphokin	COG0462	K00948	-
JAMCOH010000225.1	heat shock factor	COG0642	K15859	-
JAMCOH010000225.1	hypothetical protein		K15078	-
JAMCOH010000225.1	Glucan 1,3-alpha-glucosidase, subu	COG1501	K05546	-
JAMCOH010000225.1	hypothetical protein		K10696	-
JAMCOH010000225.1	Bromodomain-containing factor 1	COG5076	K11684	-
JAMCOH010000225.1	[Acyl-carrier-protein] acetyl transfe	COG0331	K00667,K00668	-
JAMCOH010000225.1	hypothetical protein	COG0589	-	-
JAMCOH010000225.1	hypothetical protein	COG5064	-	-
JAMCOH010000225.1	hypothetical protein	COG1739	-	-
JAMCOH010000225.1	LSU ribosomal protein L14p (L23e)	COG0093	-	-
JAMCOH010000225.1	Threonyl-tRNA synthetase (EC 6.1.1	COG0441	K01868	-
JAMCOH010000225.1	hypothetical protein		K17268	-
JAMCOH010000225.1	hypothetical protein	COG5019	K16945,K16946	-
JAMCOH010000225.1	hypothetical protein		K03351	-
JAMCOH010000225.1	Gaa1-like, GPI transamidase component		K05289	-
JAMCOH010000225.1	hypothetical protein	COG0081	K02863	-
JAMCOH010000226.1	hypothetical protein	COG5124	-	-
JAMCOH010000226.1	Fatty alcohol oxidase	COG2303	-	-
JAMCOH010000226.1	Putative GTP-ase activating proteins for the small GTPase, ARF	COG5347	K12486	-
JAMCOH010000226.1	Chaperone protein ClpB (ATP-depe	COG0542	K03695	-
JAMCOH010000226.1	hypothetical protein		K17787	-
JAMCOH010000226.1	3-oxoacyl-[acyl-carrier protein] reductase (EC 1.1.1.10		K00039,K04708	-
JAMCOH010000226.1	hypothetical protein	COG0203	K02879	-
JAMCOH010000226.1	Dihydrolipoamide dehydrogenase (	COG1249	K00382,K17583	-
JAMCOH010000226.1	Dihydrolipoamide succinyltransfera	COG0508	K00658	-
JAMCOH010000226.1	hypothetical protein		K09202,K09238	-
JAMCOH010000226.1	hypothetical protein		K09468,K21545	-
JAMCOH010000226.1	hypothetical protein	COG0099	K02952	-
JAMCOH010000226.1	hypothetical protein		K14816	-
JAMCOH010000226.1	hypothetical protein	COG0484	K09503	-
JAMCOH010000226.1	hypothetical protein		K10875,K15305	-
JAMCOH010000226.1	hypothetical protein		K17945	-
JAMCOH010000226.1	SSU ribosomal protein S29e (S14p)	COG0199	K02980	-
JAMCOH010000226.1	hypothetical protein	COG5177	K14799	-
JAMCOH010000226.1	hypothetical protein	COG1112	K14326	-
JAMCOH010000226.1	Glutathione synthetase (EC 6.3.2.3)		K21456	-
JAMCOH010000226.1	LSU ribosomal protein P2 (L7/L12)	COG2058	K02943	-
JAMCOH010000226.1	hypothetical protein	COG0197	K14001	-

JAMCOH010000226.1	Cystathionine gamma-lyase (EC 4.4.	COG0626	K01758	-
JAMCOH010000226.1	hypothetical protein	COG5184	K15462	-
JAMCOH010000226.1	Alpha-ketoglutarate-dependent tau	COG2175	K03119,K19245	-
JAMCOH010000226.1	hypothetical protein	COG2175	K03119,K19245	-
JAMCOH010000226.1	Serine/threonine phosphatases	COG0631	K01102	-
JAMCOH010000226.1	hypothetical protein		K13337	-
JAMCOH010000226.1	AAA ATPase AFG2	COG0464	K14575	-
JAMCOH010000226.1	hypothetical protein	COG4581	K12599	-
JAMCOH010000226.1	hypothetical protein	COG5078	K10577	-
JAMCOH010000226.1	6-phosphofructo-2-kinase (EC 2.7.1	COG0406	K19029	-
JAMCOH010000226.1	2-polyprenyl-6-methoxyphenol hyd	COG0654	K03380	-
JAMCOH010000226.1	SSU ribosomal protein S20e (S10p)	COG0051	K02969	-
JAMCOH010000226.1	hypothetical protein		K18192	-
JAMCOH010000226.1	hypothetical protein	COG0494	K12613,K22073	-
JAMCOH010000226.1	Serine/threonine-protein kinase RI(	COG0478	K07179	-
JAMCOH010000226.1	DnaK-like protein	COG0443	K03283	-
JAMCOH010000226.1	Formate--tetrahydrofolate ligase (E	COG2759	K00288	-
JAMCOH010000226.1	Eukaryotic translation initiation fact	COG1976	K03264	-
JAMCOH010000226.1	hypothetical protein	COG2124	K00493	-
JAMCOH010000226.1	hypothetical protein	COG2124	K00493	-
JAMCOH010000226.1	hypothetical protein	COG5032	K00888	-
JAMCOH010000226.1	hypothetical protein	COG2124	K00493	-
JAMCOH010000227.1	Adenine phosphoribosyltransferase	COG0503	K00759	-
JAMCOH010000227.1	hypothetical protein		K10418	-
JAMCOH010000227.1	hypothetical protein		K06035	-
JAMCOH010000227.1	hypothetical protein		K15165	-
JAMCOH010000227.1	hypothetical protein	COG5164	K15172	-
JAMCOH010000227.1	Transcription initiation factor IIH p4	COG5151	K03142	-
JAMCOH010000227.1	tRNA-specific adenosine deaminase	COG0590	K15441	-
JAMCOH010000227.1	hypothetical protein	COG0596	K13617	-
JAMCOH010000227.1	NAD synthetase (EC 6.3.1.5) / Gluta	COG0171	K01950	-
JAMCOH010000227.1	hypothetical protein	COG2175	-	-
JAMCOH010000227.1	Phosphatidate phosphatase (EC 3.1	COG5083	K15728	-
JAMCOH010000227.1	hypothetical protein		K13682	GT91
JAMCOH010000227.1	hypothetical protein		K13682	GT91
JAMCOH010000227.1	3'-to-5' oligoribonucleas	COG1949	K13288	-
JAMCOH010000227.1	hypothetical protein		K02266	-
JAMCOH010000227.1	hypothetical protein	COG5170	K04354	-
JAMCOH010000227.1	SSU ribosomal protein S26e	COG4830	K02976	-
JAMCOH010000227.1	hypothetical protein		K02265	-
JAMCOH010000227.1	hypothetical protein	COG1052	-	-
JAMCOH010000227.1	hypothetical protein		K20046	-
JAMCOH010000227.1	mRNA (2'-O-methyladenosine	COG4725	K05925	-
JAMCOH010000227.1	hypothetical protein	COG4539	-	-
JAMCOH010000228.1	hypothetical protein	COG5132	K12873	-
JAMCOH010000228.1	hypothetical protein	COG5025	K09413	-
JAMCOH010000228.1	hypothetical protein	COG1675	K03136	-
JAMCOH010000228.1	hypothetical protein		K11888	-
JAMCOH010000228.1	hypothetical protein		K12600	-
JAMCOH010000228.1	GTP-binding and nucleic acid-bindir	COG0012	K06942	-
JAMCOH010000228.1	hypothetical protein	COG5082	K12597	-
JAMCOH010000228.1	Cytochrome oxidase biogenesis pro	COG3346	K14998	-
JAMCOH010000228.1	hypothetical protein		K22386	-
JAMCOH010000228.1	hypothetical protein		K02307	-
JAMCOH010000228.1	Dihydroorotate dehydrogenase (qu	COG0167	K00226,K00254	-
JAMCOH010000228.1	hypothetical protein		K14768	-
JAMCOH010000228.1	Malate synthase (EC 2.3.3.9)	COG2225	K01638	-
JAMCOH010000228.1	S-adenosyl-L-methionine depender	COG2230	K20238	-
JAMCOH010000228.1	hypothetical protein		K10740	-
JAMCOH010000228.1	hypothetical protein		K14018	-
JAMCOH010000228.1	Adenylosuccinate synthetase (EC 6.	COG0104	K01939	-

JAMCOH010000228.1	Isocitrate dehydrogenase [NAD] sul	COG0473	K00030	-
JAMCOH010000229.1	hypothetical protein	COG2259	K20369	-
JAMCOH010000229.1	Cytochrome C1 heme lyase CCHL (EC 4.4.1.17)		K01764	-
JAMCOH010000229.1	hypothetical protein		K17778,K17781	-
JAMCOH010000229.1	hypothetical protein	COG0661	K08869	-
JAMCOH010000229.1	hypothetical protein		K12825	-
JAMCOH010000229.1	SSU ribosomal protein S6e	COG2125	K02991	-
JAMCOH010000229.1	hypothetical protein	COG5068	K09265,K19807	-
JAMCOH010000229.1	Acetyltransferase		K20794	-
JAMCOH010000229.1	hypothetical protein	COG5309	K01210	-
JAMCOH010000229.1	Ferredoxin--NADP(+) reductase (EC	COG0493	K18914	-
JAMCOH010000229.1	hypothetical protein		K11663	-
JAMCOH010000229.1	hypothetical protein		K17968	-
JAMCOH010000229.1	hypothetical protein		K11566	-
JAMCOH010000229.1	hypothetical protein		K21597	-
JAMCOH010000229.1	hypothetical protein	COG0154	K01426	-
JAMCOH010000230.1	hypothetical protein	COG5167	K15445	-
JAMCOH010000230.1	hypothetical protein (N-acetyltrans	COG0454	K18852	-
JAMCOH010000230.1	Oxidoreductase, short-chain dehyd	COG1028	K17738	-
JAMCOH010000230.1	hypothetical protein		K11841	-
JAMCOH010000230.1	hypothetical protein	COG1167	-	-
JAMCOH010000230.1	hypothetical protein	COG0671	K18693	-
JAMCOH010000230.1	hypothetical protein (Helical region found in SNAREs)		K08502	-
JAMCOH010000230.1	hypothetical protein	COG0596	-	-
JAMCOH010000230.1	hypothetical protein	COG1716	K08994	-
JAMCOH010000230.1	hypothetical protein	COG1716	K08994	-
JAMCOH010000230.1	hypothetical protein	COG1269	K02154	-
JAMCOH010000230.1	hypothetical protein	COG0790	K14023,K14026	-
JAMCOH010000230.1	hypothetical protein	COG1112	K10742	-
JAMCOH010000230.1	Nucleolar RNA processing protein N	COG1498	K14565	-
JAMCOH010000230.1	hypothetical protein	COG0457	-	-
JAMCOH010000230.1	Serine/Threonine protein kinases, catalytic domain		K06276	-
JAMCOH010000230.1	Mitochondrial intermediate peptida	COG0339	K01410	-
JAMCOH010000230.1	glutamine permease	COG0531	K16261	-
JAMCOH010000230.1	Seryl-tRNA synthetase (EC 6.1.1.11)	COG0172	K01875	-
JAMCOH010000230.1	hypothetical protein		K08518	-
JAMCOH010000230.1	hypothetical protein		K09658	GT2
JAMCOH010000230.1	hypothetical protein		K12398	-
JAMCOH010000230.1	Multicopper oxidase	COG2132	K19791	-
JAMCOH010000230.1	Multicopper oxidase	COG2132	K19791	-
JAMCOH010000230.1	hypothetical protein	COG0596	K13535	-
JAMCOH010000230.1	hypothetical protein	COG0476	K10686	-
JAMCOH010000230.1	hypothetical protein	COG5055	K10873	-
JAMCOH010000230.1	hypothetical protein	COG1958	K11097	-
JAMCOH010000230.1	hypothetical protein	COG1948	K08991	-
JAMCOH010000230.1	hypothetical protein		K14315	-
JAMCOH010000230.1	hypothetical protein	COG0025	K12041	-
JAMCOH010000230.1	Triose-phosphate Transporter family		K15283	-
JAMCOH010000230.1	hypothetical protein	COG1902	K00354	-
JAMCOH010000230.1	Ribosomal protein L1p/L10e family		K14775,K14783	-
JAMCOH010000230.1	Glutathione peroxidase (EC 1.11.1.9	COG0386	K00432	-
JAMCOH010000230.1	hypothetical protein	COG2319	K03363	-
JAMCOH010000230.1	hypothetical protein		K05756	-
JAMCOH010000230.1	hypothetical protein	COG2020	K00587	-
JAMCOH010000230.1	hypothetical protein Isoprenylcyste	COG2020	K00587	-
JAMCOH010000230.1	acid desaturase	COG3239	K10256	-
JAMCOH010000230.1	hypothetical protein		K19950	-
JAMCOH010000230.1	hypothetical protein		K10868	-

JAMCOH010000230.1	Lysine-specific permease	COG0531	K16261	-
JAMCOH010000230.1	Tyrosyl-tRNA synthetase (EC 6.1.1.1	COG0162	K01866	-
JAMCOH010000230.1	RNA helicase	COG1643	K12818,K14781,K17820	-

Table S4: Annotation data for proteins obtained from Bin002 obtained from shotgun metagenome sequence of *Candida tropicalis* JY101
the locus tag, predicted product, KEGG/COG/CAZY annotation are provided (if available)

Locus Tag	Product	COG	KEGG	CAZY
JAMCOI010000001.1	hypothetical protein		K12386	-
JAMCOI010000001.1	hypothetical protein	COG0477	-	-
JAMCOI010000001.1	MICOS complex subunit MIC10		K17784	-
JAMCOI010000001.1	hypothetical protein	COG0484	K09537,K10357	-
JAMCOI010000001.1	N(6)-L-threonylcarbamoyladenine synthase (EC 2.3.1.234)	COG0533	K01409	-
JAMCOI010000001.1	hypothetical protein		K11573	-
JAMCOI010000001.1	LSU ribosomal protein L30e	COG1911	K02908	-
JAMCOI010000001.1	LSU ribosomal protein L24e	COG2075	K02896	-
JAMCOI010000001.1	Asparaginase	COG1446	K08657	-
JAMCOI010000001.1	Cytochrome b5 domain / Sphingolipid (R)-alpha-hydroxylase FAH1 (no EC)	COG3000	K19703	-
JAMCOI010000001.1	Ribonuclease III (EC 3.1.26.3)	COG0571	K03685	-
JAMCOI010000001.1	Pyruvate dehydrogenase E1 component beta subunit (EC 1.2.4.1)	COG0022	K00162	-
JAMCOI010000001.1	acyl-CoA thioester hydrolase	COG1607	K17361	-
JAMCOI010000001.1	Dystroglycan-type cadherin-like domains.		K18637	-
JAMCOI010000001.1	hypothetical protein		K00988	-
JAMCOI010000001.1	Acetyltransferase		-	-
JAMCOI010000001.1	hypothetical protein	COG5088	K15153	-
JAMCOI010000002.1	GPR1/FUN34/yaaH family	COG1584	K07034	-
JAMCOI010000002.1	Mannosyltransferase putative		K05538	GT71
JAMCOI010000002.1	hypothetical protein		K02605	-
JAMCOI010000002.1	Diacylglycerol acyltransferase		K14457,K14458	-
JAMCOI010000002.1	5'-methylthioadenosine phosphorylase (EC 2.4.2.28)	COG0005	K00772	-
JAMCOI010000002.1	Peptide transporter	COG3104	K03305	-
JAMCOI010000002.1	Peptide transporter	COG3104	K03305	-
JAMCOI010000002.1	hypothetical protein		K02831,K06641	-
JAMCOI010000002.1	hypothetical protein		K17799	-
JAMCOI010000002.1	hypothetical protein	COG5057	K17605	-
JAMCOI010000002.1	hypothetical protein	COG5623	K14399	-
JAMCOI010000002.1	oligopeptide transporter		-	-
JAMCOI010000002.1	oligopeptide transporter		-	-
JAMCOI010000002.1	hypothetical protein	COG5190	K15731	-
JAMCOI010000003.1	Glucan synthase subunit	COG2273	K21850	GH16
JAMCOI010000003.1	hypothetical protein	COG2072	-	-
JAMCOI010000003.1	hypothetical protein		K18624	-
JAMCOI010000003.1	25S rRNA (uridine(2634)-N(3))-methyltransferase (EC 2.1.1.313)		K19307	-
JAMCOI010000003.1	Glutathione S-transferase	COG0625	K00799	-
JAMCOI010000003.1	hypothetical protein		K11094	-
JAMCOI010000003.1	hypothetical protein		K17673	-
JAMCOI010000003.1	Long chain fatty acyl-CoA synthetase	COG1022	K01897	-
JAMCOI010000004.1	hypothetical protein	COG0814	K15015	-
JAMCOI010000004.1	hypothetical protein		K12763	-
JAMCOI010000004.1	4-hydroxyphenylpyruvate dioxygenase (EC 1.13.11.27)	COG3185	K00457	-
JAMCOI010000004.1	RTA1 like protein		-	-
JAMCOI010000004.1	Afadin- and alpha -actinin-Binding		-	-
JAMCOI010000004.1	RF-1 domain	COG0216	-	-
JAMCOI010000004.1	Tyrosine phosphatase family	COG2365	K18044,K18045	-
JAMCOI010000004.1	Hydroxyacylglutathione hydrolase (EC 3.1.2.6)	COG0491	K01069	-
JAMCOI010000004.1	hypothetical protein	COG5091	K12795	-
JAMCOI010000004.1	O-acyltransferase	COG5056	K00637	-
JAMCOI010000004.1	Sterol O-acyltransferase (EC 2.3.1.26)	COG5056	K00637	-
JAMCOI010000005.1	hypothetical protein	COG0819	-	-
JAMCOI010000005.1	Hydroxymethylpyrimidine synthesis protein THI5	COG0715	K18278	-
JAMCOI010000005.1	cytochrome c oxidase assembly protein	COG1999	K07152	-
JAMCOI010000005.1	SET domain		-	-
JAMCOI010000005.1	Phosphate transport (Pho88)	COG5065	-	-
JAMCOI010000005.1	hypothetical protein	COG1525	K15979	-
JAMCOI010000005.1	ubiquitin-conjugating enzyme		K04555	-
JAMCOI010000005.1	Flavin containing amine oxidoreductase	COG1231	K13367	-
JAMCOI010000005.1	hypothetical protein Striatin family		-	-
JAMCOI010000005.1	clavaminate synthase-like protein		-	-
JAMCOI010000005.1	Phytanoyl-CoA dioxygenase (PhyH)	COG5285	-	-

JAMCOI010000005.1	Cysteine dioxygenase (EC 1.13.11.20)		K00456	-
JAMCOI010000005.1	hypothetical protein	COG1275	-	-
JAMCOI010000005.1	High-affnity carbon uptake protein Hat/HatR		K12662	-
JAMCOI010000005.1	hypothetical protein ThiF family	COG0476	K10684	-
JAMCOI010000005.1	D-glycerate 3-kinase (EC 2.7.1.31), plant type	COG4240	K15918	-
JAMCOI010000005.1	Electron transfer flavoprotein, beta subunit	COG2086	K03521	-
JAMCOI010000005.1	hypothetical protein		K16803	-
JAMCOI010000005.1	C-terminal of NADH-ubiquinone oxidoreductase 21 kDa subunit		-	-
JAMCOI010000005.1	hypothetical protein		K13348	-
JAMCOI010000005.1	POC1 chaperone		K11879	-
JAMCOI010000005.1	hypothetical protein	COG5590	K18587	-
JAMCOI010000005.1	Prefoldin subunit	COG1382	K04798	-
JAMCOI010000005.1	hypothetical protein		K02619	-
JAMCOI010000005.1	hypothetical protein		K05747	-
JAMCOI010000005.1	putative membrane transporter		-	-
JAMCOI010000005.1	Phospholipase B		K13333	-
JAMCOI010000005.1	S-formylglutathione hydrolase (EC 3.1.2.12)	COG0627	K01070	CE1
JAMCOI010000005.1	Uncharacterized Nudix hydrolase NudL	COG0494	-	-
JAMCOI010000005.1	hypothetical protein		K14859	-
JAMCOI010000005.1	Required for maturation of the 35S primary transcript of pre-rRNA and is required for cleavages leading to mature 18S RNA	COG0513	K14777	-
JAMCOI010000005.1	hypothetical protein		K15152	-
JAMCOI010000005.1	protein-O-mannosyltransferase	COG1928	K00728	GT39
JAMCOI010000005.1	D-arabinono-1,4-lactone oxidase	COG0277	K00103,K00107	-
JAMCOI010000005.1	hypothetical protein	COG5644	K14567	-
JAMCOI010000005.1	Protein SAP1	COG0464	K01509,K20293	-
JAMCOI010000005.1	Uncharacterized MFS-type transporter		-	-
JAMCOI010000005.1	hypothetical protein	COG0531	K16261	-
JAMCOI010000005.1	DNA polymerase epsilon subunit		K02325	-
JAMCOI010000005.1	hypothetical protein		K16362	-
JAMCOI010000005.1	geranylgeranyltransferase beta subunit	COG5029	K05956	-
JAMCOI010000005.1	hypothetical protein		K12772	-
JAMCOI010000005.1	Nucleolar RNA processing protein Nop56	COG1498	K14564	-
JAMCOI010000005.1	hypothetical protein		K09245	-
JAMCOI010000005.1	hypothetical protein ubiquitin-conjugating enzyme family	COG5078	K06689	-
JAMCOI010000005.1	Transcriptional activator SPT7	COG5076	K11359	-
JAMCOI010000005.1	hypothetical protein		K04563,K05916	-
JAMCOI010000005.1	hypothetical protein		K14024	-
JAMCOI010000005.1	hypothetical protein ankyrin repeats	COG5184	-	-
JAMCOI010000005.1	6-O-methylguanine DNA methyltransferase	COG0350	K07443	-
JAMCOI010000006.1	Amino acid permease	COG0531	K16261	-
JAMCOI010000006.1	C-terminal region of Mon2 protein		-	-
JAMCOI010000006.1	Chitin synthase	COG1215	K00698	GT2
JAMCOI010000006.1	fatty acid desaturase	COG3239	K10256	-
JAMCOI010000006.1	Translation elongation factor G	COG0480	K02355	-
JAMCOI010000006.1	ribosomal protein	COG2163	K02875	-
JAMCOI010000006.1	LSU ribosomal protein L14e	COG2163	K02875	-
JAMCOI010000006.1	Cysteinyl-tRNA synthetase (EC 6.1.1.16)	COG0215	K01883	-
JAMCOI010000006.1	Signal peptidase		K12948	-
JAMCOI010000006.1	hypothetical protein		K17669	-
JAMCOI010000007.1	Enoyl-(Acyl carrier protein) reductase	COG1028	K17742	-
JAMCOI010000007.1	Oxidoreductase, short-chain dehydrogenase/reductase family	COG1028	K17742	-
JAMCOI010000007.1	V-type ATP synthase subunit D (EC 3.6.3.14)	COG1394	K02149	-
JAMCOI010000007.1	hypothetical protein		K12867	-
JAMCOI010000007.1	AFG1-like ATPase	COG1485	K18798	-
JAMCOI010000007.1	Nucleotide-sugar transporter	COG0697	-	-
JAMCOI010000007.1	YL1 nuclear protein C-terminal domain	COG5195	K11667	-
JAMCOI010000007.1	hypothetical protein		K20312	-
JAMCOI010000007.1	hypothetical protein of protein kinase		K04371	-
JAMCOI010000007.1	DNA primase	COG2219	K01101,K02685	-
JAMCOI010000007.1	hypothetical protein	COG5406	K01262	-
JAMCOI010000007.1	hypothetical protein	COG5057	K17605	-
JAMCOI010000007.1	RNase P subunit Pop3		K14522	-
JAMCOI010000007.1	Glutamate N-acetyltransferase (EC 2.3.1.35) @ N-acetylglutamate synthase (EC 2.3.1.1)	COG0470	K10755	-
JAMCOI010000007.1	hypothetical protein of Vps52 / Sac2 family		K20298	-
JAMCOI010000007.1	ATP-dependent DNA helicase UvrD/PcrA (EC 3.6.4.12)	COG0210	K19781	-
JAMCOI010000008.1	Succinate dehydrogenase flavoprotein subunit (EC 1.3.5.1)	COG1053	K00234	-
JAMCOI010000008.1	proteasome regulatory subunit Rpt1	COG0554	K03061	-

JAMCOI010000008.1	RNA polymerase III	COG1095	K03022	-
JAMCOI010000008.1	hypothetical protein	COG0666	K06653	-
JAMCOI010000008.1	Haloacid dehalogenase-like hydrolase	COG0647	-	-
JAMCOI010000008.1	hypothetical protein		K12389	-
JAMCOI010000008.1	2-keto-3-deoxy-D-arabino-heptulosonate-7-phosphate synthase I alpha (EC 2.5.1.54)	COG0722	K01626	-
JAMCOI010000008.1	hypothetical protein	COG1272	K07297	-
JAMCOI010000008.1	N-acetylglutamate synthase	COG0548	K00618	-
JAMCOI010000009.1	Aspartate aminotransferase	COG1448	K14454	-
JAMCOI010000009.1	catalytic domain of ctd-like phosphatases	COG5190	K17617	-
JAMCOI010000009.1	hypothetical protein		K19619	-
JAMCOI010000009.1	CoA-transferase family III	COG1804	-	-
JAMCOI010000009.1	Alpha/beta hydrolase family	COG0596	-	-
JAMCOI010000009.1	Catechol 1,2-dioxygenase 1 (EC 1.13.11.1)	COG3485	K03381	-
JAMCOI010000009.1	glycosyl hydrolase 17 family	COG5309	K01210	-
JAMCOI010000009.1	Putative RNA methyltransferase	COG2106	K09142	-
JAMCOI010000009.1	Isopentenyl-diphosphate Delta-isomerase (EC 5.3.3.2)	COG1443	K01823	-
JAMCOI010000009.1	Lipoyl synthase (EC 2.8.1.8)	COG0320	K03644	-
JAMCOI010000009.1	histone deacetylase	COG0123	K11484	-
JAMCOI010000009.1	GTPase-activator protein for Rho-like GTPases		K08773,K19840	-
JAMCOI010000010.1	Octaprenyl diphosphate synthase (EC 2.5.1.90)	COG0142	K05355	-
JAMCOI010000010.1	hypothetical protein	COG5171	K15306	-
JAMCOI010000010.1	hypothetical protein	COG0584	K06653	-
JAMCOI010000010.1	Trehalase (EC 3.2.1.28)	COG1626	K01194	GH37
JAMCOI010000010.1	Malate dehydrogenase (EC 1.1.1.37)	COG0039	K00026	-
JAMCOI010000010.1	hypothetical protein	COG5101	K14290	-
JAMCOI010000010.1	LSU ribosomal protein L6p (L9e)	COG0097	K02933	-
JAMCOI010000010.1	LSU ribosomal protein L3p (L3e)	COG0087	K02906	-
JAMCOI010000010.1	hypothetical protein	COG0522	K14560	-
JAMCOI010000010.1	hypothetical protein		K22493	-
JAMCOI010000011.1	hypothetical protein belonging to Belongs to the glutaredoxin family	COG0354	K22073	-
JAMCOI010000011.1	Purine nucleoside phosphorylase (EC 2.4.2.1)	COG0005	K03783	-
JAMCOI010000011.1	hypothetical protein		K14004	-
JAMCOI010000011.1	Cytosine/purine/uracil/thiamine/allantoin permease family protein	COG1953	K03457	-
JAMCOI010000011.1	nitrosoguanidine resistance protein		K07101	-
JAMCOI010000011.1	hypothetical protein		K11362	-
JAMCOI010000011.1	hypothetical protein	COG5059	-	-
JAMCOI010000011.1	hypothetical protein		K08165	-
JAMCOI010000012.1	Chitin synthase		-	-
JAMCOI010000012.1	hypothetical protein		K17775	-
JAMCOI010000012.1	hypothetical protein	COG5594	K21989	-
JAMCOI010000012.1	hypothetical protein		K09243,K21547	-
JAMCOI010000012.1	hypothetical protein		K01178	GH15
JAMCOI010000013.1	Ferric reductase		-	-
JAMCOI010000013.1	Fatty acid desaturase (EC 1.14.19.1); Delta-9 fatty acid desaturase (EC 1.14.19.1)	COG1398	K00507	-
JAMCOI010000013.1	hypothetical protein	COG5391	K17918	-
JAMCOI010000013.1	Alcohol dehydrogenase (EC 1.1.1.1)	COG1064	K13953	-
JAMCOI010000013.1	Squalene	COG0654	K00511	-
JAMCOI010000013.1	Translation initiation factor 2B epsilon subunit	COG1208	K03240	-
JAMCOI010000013.1	Copper transport protein		K14686	-
JAMCOI010000013.1	Mannosyltransferase		K03846	GT22
JAMCOI010000013.1	Histidine Phosphotransfer domain		K11232	-
JAMCOI010000013.1	4-nitrophenylphosphatase	COG0647	K01101	-
JAMCOI010000013.1	hypothetical protein		K10736	-
JAMCOI010000013.1	hypothetical protein		K20290	-
JAMCOI010000013.1	hypothetical protein		K10845	-
JAMCOI010000014.1	ubiquitin protease	COG1310	K03249	-
JAMCOI010000014.1	hypothetical protein	COG5333	K15563	-
JAMCOI010000014.1	Alcohol dehydrogenase	COG1063	-	-
JAMCOI010000014.1	Thioredoxin reductase (EC 1.8.1.9)	COG0492	K00344,K00384	-
JAMCOI010000014.1	hypothetical protein belongimng to syntaxin family	COG5325	-	-
JAMCOI010000014.1	cytosolic iron-sulfur (Fe S) protein		-	-
JAMCOI010000014.1	Oxidoreductase FAD-binding domain	COG0543	-	-
JAMCOI010000014.1	hypothetical protein		K11293	-
JAMCOI010000014.1	hypothetical protein	COG5041	K03115	-
JAMCOI010000014.1	Tryptophanyl-tRNA synthetase (EC 6.1.1.2)	COG0180	K01867,K02946	-
JAMCOI010000014.1	hypothetical protein		K09467	-
JAMCOI010000015.1	Alpha/beta hydrolase family		-	-

JAMCOI010000015.1	Pantoate--beta-alanine ligase (EC 6.3.2.1)	COG0414	K01918	-
JAMCOI010000015.1	ATP-dependent helicase HrpA	COG1643	K12815	-
JAMCOI010000015.1	Nucleoporin complex subunit 54		K14308	-
JAMCOI010000015.1	hypothetical protein		K03952	-
JAMCOI010000015.1	hypothetical protein		K20289	-
JAMCOI010000015.1	hypothetical protein		K09274,K22496	-
JAMCOI010000016.1	Chaperone protein DnaJ	COG0484	K14002	-
JAMCOI010000016.1	Trypsin-like peptidase domain		-	-
JAMCOI010000016.1	Sugar (and other) transporter		-	-
JAMCOI010000016.1	DNA Polymerase	COG0749	K02332	-
JAMCOI010000017.1	hypothetical protein	COG0657	-	-
JAMCOI010000017.1	hypothetical protein		K04409	-
JAMCOI010000017.1	Haloacid dehalogenase-like hydrolase	COG0647	-	-
JAMCOI010000017.1	hypothetical protein	COG5291	K13989	-
JAMCOI010000017.1	Coenzyme F420-dependent N5,N10-methylene tetrahydromethanopterin reductase and related flavin-dependent oxidoreductases	COG2141	K04091	-
JAMCOI010000017.1	hypothetical protein		K17967	-
JAMCOI010000017.1	hypothetical protein belonging to syntaxin family		K08498,K08499	-
JAMCOI010000018.1	hypothetical protein		K14967	-
JAMCOI010000018.1	Glycosyl hydrolases family 16	COG2273	-	-
JAMCOI010000018.1	6-phosphofructokinase, fungal/animal type (EC 2.7.1.11)	COG0205	K00850	-
JAMCOI010000018.1	heat shock factor	COG5169	-	-
JAMCOI010000018.1	Got1/Sft2-like family	COG0284	-	-
JAMCOI010000018.1	Delta-1-pyrroline-5-carboxylate dehydrogenase (EC 1.2.1.88)	COG1012	K00294	-
JAMCOI010000018.1	Dynamitin		K18599	-
JAMCOI010000018.1	GTP-binding protein RBG2	COG1163	K06944	-
JAMCOI010000019.1	hypothetical protein		K20178	-
JAMCOI010000019.1	Chitin synthase (EC 2.4.1.16)	COG1215	K00698	GT2
JAMCOI010000019.1	plasma membrane ATPase	COG0474	K01535	-
JAMCOI010000019.1	hypothetical protein	COG5099	-	-
JAMCOI010000020.1	Peptidyl-tRNA hydrolase	COG0193	K01056	-
JAMCOI010000020.1	Protein tyrosine kinase		K08286	-
JAMCOI010000020.1	Protein kinase		K11228	-
JAMCOI010000020.1	Calcineurin-like phosphoesterase	COG1409	-	-
JAMCOI010000020.1	hypothetical protein		K09313	-
JAMCOI010000020.1	LSU ribosomal protein L21e	COG2139	K02889	-
JAMCOI010000020.1	SSU ribosomal protein S9e (S4p)	COG0522	K02997	-
JAMCOI010000020.1	Diphthine--ammonia ligase (EC 6.3.1.14)	COG0251	K06927	-
JAMCOI010000021.1	Acetylornithine deacetylase/Succinyl-diaminopimelate desuccinylase and related deacylases	COG0624	K15428	-
JAMCOI010000021.1	hypothetical protein	COG5053	-	-
JAMCOI010000021.1	hypothetical protein	COG5641	K09184	-
JAMCOI010000021.1	Adenosylhomocysteinase (EC 3.3.1.1)	COG0499	K01251	-
JAMCOI010000021.1	hypothetical protein		K01166	-
JAMCOI010000021.1	hypothetical protein	COG0400	K06128,K06130,K	-
JAMCOI010000022.1	Putative Sin3 binding protein		-	-
JAMCOI010000022.1	DNA replication licensing factor MCM7	COG1241	K02210	-
JAMCOI010000022.1	hypothetical protein		K18732	-
JAMCOI010000022.1	Fumarylacetoacetate hydrolase family protein	COG0179	-	-
JAMCOI010000022.1	Ydr279p protein family (RNase H2 complex component)		-	-
JAMCOI010000022.1	hypothetical protein	COG0330	-	-
JAMCOI010000022.1	3' exoribonuclease family, domain 2	COG2123	K03678	-
JAMCOI010000022.1	Glycosyltransferase	COG3774	-	-
JAMCOI010000023.1	Ancient conserved domain protein 4	COG1253	K16302	-
JAMCOI010000023.1	Protein tyrosine kinase		-	-
JAMCOI010000023.1	hypothetical protein	COG0814	K14209	-
JAMCOI010000024.1	Enoyl-(Acyl carrier protein) reductase	COG1028	K13237	-
JAMCOI010000024.1	N(6)-L-threonylcarbamoyladenine synthase (EC 2.3.1.234)	COG0533	K01409	-
JAMCOI010000024.1	hypothetical protein	COG5125	-	-
JAMCOI010000024.1	DNA mismatch repair protein MutL	COG0323	K08734	-
JAMCOI010000024.1	hypothetical protein		K21845	-
JAMCOI010000024.1	hypothetical protein	COG0023	-	-
JAMCOI010000025.1	hypothetical protein	COG5017	K07432	GT1
JAMCOI010000025.1	C-8 sterol isomerase		K09829	-
JAMCOI010000025.1	hypothetical protein	COG5024	K06650,K12760	-
JAMCOI010000025.1	hypothetical protein		K12182	-
JAMCOI010000025.1	hypothetical protein		K12182	-
JAMCOI010000025.1	Pyridoxal 5-phosphate (PLP)-dependent ornithine decarboxylase (EC 4.1.1.17)	COG0019	K01581	-

JAMCOI010000025.1	serine threonine-protein phosphatase	COG0639	K04348	-
JAMCOI010000026.1	Transcription initiation factor IIF alpha subunit		K03138	-
JAMCOI010000026.1	Flavin containing amine oxidoreductase	COG1231	K13367	-
JAMCOI010000026.1	hypothetical protein		K00624	-
JAMCOI010000026.1	Ceramide glucosyltransferase	COG1171	K00720	GT21
JAMCOI010000027.1	hypothetical protein	COG0692	K03648	-
JAMCOI010000027.1	hypothetical protein		K12669	-
JAMCOI010000027.1	hypothetical protein	COG0697	K15289	-
JAMCOI010000027.1	Endonuclease IV (EC 3.1.21.2)	COG0648	K10771	-
JAMCOI010000027.1	Nucleoporin autopeptidase		K14297,K18720	-
JAMCOI010000027.1	Glycosyl transferase family 8	COG5597	K00750	GT8
JAMCOI010000027.1	hypothetical protein	COG0011	-	-
JAMCOI010000028.1	COP9 signalosome subunit 5	COG1310	K09613	-
JAMCOI010000028.1	Ubiquitin carboxyl-terminal hydrolase 10	COG5560	K11873	-
JAMCOI010000028.1	LSU ribosomal protein L22p (L17e), mitochondrial	COG0091	-	-
JAMCOI010000028.1	NADH-ubiquinone oxidoreductase 9.5 kDa subunit		-	-
JAMCOI010000028.1	hypothetical protein	COG4886	-	-
JAMCOI010000028.1	Ferredoxin, 2Fe-2S	COG0633	K22071	-
JAMCOI010000028.1	Vesicle transport v-SNARE protein N-terminus		K08493	-
JAMCOI010000029.1	hypothetical protein	COG5391	K17919	-
JAMCOI010000029.1	Mannan endo-1,6-alpha-mannosidase (EC 3.2.1.101)		K08257	GH76
JAMCOI010000029.1	lysophospholipase		K13333	-
JAMCOI010000029.1	hypothetical protein		K12471	-
JAMCOI010000029.1	hypothetical protein		K20780	-
JAMCOI010000029.1	hypothetical protein	COG5024	K02220	-
JAMCOI010000030.1	L-lysine 6-monooxygenase (NADPH-requiring)		-	-
JAMCOI010000030.1	heat shock transcription factor	COG5169	K09419	-
JAMCOI010000030.1	hypothetical protein		K11246	-
JAMCOI010000030.1	oxidoreductase of aldo/keto reductase family, subgroup 1	COG0656	K18097	-
JAMCOI010000031.1	tubulin gamma	COG5023	K10389	-
JAMCOI010000031.1	Aromatic amino acid aminotransferase	COG1167	K05821	-
JAMCOI010000031.1	Bin/amphiphysin/Rvs domain for vesicular trafficking		-	-
JAMCOI010000031.1	Aspartokinase (EC 2.7.2.4)	COG0527	K00928	-
JAMCOI010000031.1	2OG-Fe(II) oxygenase superfamily	COG3751	-	-
JAMCOI010000031.1	NADH cytochrome b5 reductase	COG0543	K00326	-
JAMCOI010000031.1	Lysine-specific permease	COG0531	K16261	-
JAMCOI010000031.1	hypothetical protein Rap/ran-GAP		-	-
JAMCOI010000031.1	D-arabinose-5-phosphate isomerase (EC 5.3.1.13)	COG0794	-	-
JAMCOI010000032.1	NADH-ubiquinone oxidoreductase chain D (EC 1.6.5.3)	COG0649	K03935	-
JAMCOI010000032.1	hypothetical protein	COG0484	-	-
JAMCOI010000032.1	ubiquitin-conjugating enzyme	COG5078	K02207	-
JAMCOI010000032.1	hypothetical protein	COG2966	-	-
JAMCOI010000032.1	hypothetical protein		K20040	-
JAMCOI010000032.1	hypothetical protein	COG5249	-	-
JAMCOI010000032.1	hypothetical protein	COG0211	K00030,K02899	-
JAMCOI010000032.1	hypothetical protein	COG4702	-	-
JAMCOI010000033.1	phospholipase c		K01771	-
JAMCOI010000033.1	Lysine methyltransferase		-	-
JAMCOI010000033.1	Aspartate aminotransferase (EC 2.6.1.1)	COG0436	K14264	-
JAMCOI010000033.1	Precorrin-2 oxidase (EC 1.3.1.76) @ Sirohydrochlorin ferrochelatase activity of CysG (EC 4.99.1.4) / Uroporphyrinogen-III methyltransferase (EC 2.1.1.107)	COG0007	K00589	-
JAMCOI010000033.1	hypothetical protein	COG3582	K07059	-
JAMCOI010000033.1	Glycerol-3-phosphate O-acyltransferase (EC 2.3.1.15) @ Glycerone-phosphate O-acyltransferase (EC 2.3.1.42)	COG0204	K13507	-
JAMCOI010000033.1	NADH:flavin oxidoreductases, Old Yellow Enzyme family	COG1902	K00354	-
JAMCOI010000033.1	Protein tyrosine phosphatase, catalytic domain	COG5599	K01104	-
JAMCOI010000033.1	hypothetical protein		K20302	-
JAMCOI010000033.1	Fatty acid hydroxylase family (carotene hydroxylase/sterol desaturase)	COG3000	K04713	-
JAMCOI010000033.1	hypothetical protein	COG0531	K09885	-
JAMCOI010000034.1	hypothetical protein	COG2939	K13289	-
JAMCOI010000034.1	hypothetical protein	COG5273	K18932	-
JAMCOI010000034.1	hypothetical protein		K20826	-
JAMCOI010000034.1	RNAse P Rpr2/Rpp21/SNM1 subunit domain		K14531	-
JAMCOI010000034.1	Ferric reductase (1.6.99.14)		-	-
JAMCOI010000035.1	hypothetical protein		K17262	-
JAMCOI010000035.1	hypothetical protein		K14793	-
JAMCOI010000035.1	DNA-directed RNA polymerase III 31 kDa polypeptide (EC 2.7.7.6)		K03024	-

JAMCOI010000035.1	Protein kinase C conserved region 2 (CalB)		-	-
JAMCOI010000035.1	Deacetylases, including yeast histone deacetylase and acetoin utilization protein	COG0123	K06067	-
JAMCOI010000035.1	Dolichyl-phosphate beta-glucosyltransferase (EC 2.4.1.117)	COG0463	K00729	GT2
JAMCOI010000035.1	Phosphoglucomutase (EC 5.4.2.2)	COG0033	K01835	-
JAMCOI010000035.1	hypothetical protein	COG5641	K09184	-
JAMCOI010000036.1	hypothetical protein		K19849	-
JAMCOI010000036.1	hypothetical protein		K13217	-
JAMCOI010000036.1	hypothetical protein		K15082	-
JAMCOI010000036.1	hypothetical protein	COG5272	K10839	-
JAMCOI010000036.1	2,3-diketo-5-methylthiopentyl-1-phosphate enolase-phosphatase (EC 3.1.3.77)	COG4229	K09880	-
JAMCOI010000036.1	hypothetical protein	COG1752	-	-
JAMCOI010000037.1	S-(hydroxymethyl)glutathione dehydrogenase (EC 1.1.1.284)	COG1062	K00121	-
JAMCOI010000037.1	hypothetical protein		K14546	-
JAMCOI010000037.1	hypothetical protein		K11370	-
JAMCOI010000037.1	hypothetical protein		K10426	-
JAMCOI010000037.1	L-asparaginase (EC 3.5.1.1)	COG0252	K01424	-
JAMCOI010000037.1	Long-chain-fatty-acid--CoA ligase (EC 6.2.1.3)	COG1022	K01897	-
JAMCOI010000037.1	Inositol-1-phosphate synthase (EC 5.5.1.4)	COG1260	K01858	-
JAMCOI010000037.1	hypothetical protein		K01183	GH18
JAMCOI010000038.1	S-adenosylmethionine decarboxylase proenzyme (EC 4.1.1.50), eukaryotic		K01611	-
JAMCOI010000038.1	RNA polymerase II transcription factor B	COG5220	K10842	-
JAMCOI010000038.1	Protein arginine N-methyltransferase 2 (EC 2.1.1.322)		K18477	-
JAMCOI010000038.1	hypothetical protein	COG2124	K05917	-
JAMCOI010000038.1	Homoserine kinase (EC 2.7.1.39)	COG0083	K00872	-
JAMCOI010000038.1	hypothetical protein	COG4775	-	-
JAMCOI010000038.1	hypothetical protein	COG0258	K10746	-
JAMCOI010000038.1	hypothetical protein		K15901	-
JAMCOI010000038.1	GTPase activating factor for Rsr1p Bud1p		-	-
JAMCOI010000038.1	hypothetical protein	COG1112	K14326	-
JAMCOI010000038.1	sphingomyelin phosphodiesterase		-	-
JAMCOI010000038.1	Ubiquitin-conjugating enzyme family	COG5078	K10580	-
JAMCOI010000038.1	hypothetical protein	COG0474	K01509,K01530,K	-
JAMCOI010000038.1	ADP,ATP carrier protein		K05863	-
JAMCOI010000038.1	Multicopper oxidase	COG2132	K19791	-
JAMCOI010000038.1	hypothetical protein	COG2273	K21850	GH16
JAMCOI010000038.1	hypothetical protein Belongs to the SNF7 family	COG5491	K12191	-
JAMCOI010000038.1	Adenylylsulfate kinase (EC 2.7.1.25)	COG0529	K00860	-
JAMCOI010000038.1	hypothetical protein	COG0360	K02990	-
JAMCOI010000038.1	hypothetical protein		K05765	-
JAMCOI010000038.1	D-amino acid oxidase	COG0665	K00272,K00273	-
JAMCOI010000038.1	Chaperone protein DnaJ	COG0484	K09503	-
JAMCOI010000038.1	Ribosomal protein L34		K02914	-
JAMCOI010000038.1	hypothetical protein		K03001	-
JAMCOI010000039.1	hypothetical protein	COG0695	K03676	-
JAMCOI010000039.1	tRNA-splicing ligase (EC 6.5.1.3)	COG5324	K14679	-
JAMCOI010000039.1	hypothetical protein		K07195	-
JAMCOI010000040.1	ATP-dependent RNA helicase	COG1643	K13026	-
JAMCOI010000041.1	SAC3/GANP family		-	-
JAMCOI010000041.1	ubiquitin carboxyl-terminal hydrolase		K05609	-
JAMCOI010000041.1	SSU ribosomal protein S14e (S11p)	COG0100	K02955	-
JAMCOI010000041.1	SSU ribosomal protein S15Ae (S8p)	COG0096	K02957	-
JAMCOI010000041.1	hypothetical protein		K20359	-
JAMCOI010000042.1	hypothetical protein		K21388	-
JAMCOI010000042.1	hypothetical protein	COG0625	K00799,K01872,K	-
JAMCOI010000042.1	Translation elongation factor 1 gamma subunit	COG0625	K01120,K03233	-
JAMCOI010000043.1	hypothetical protein	COG4886	-	-
JAMCOI010000043.1	beta-glucosidase (EC 3.2.1.21)	COG1472	K05349	GH3
JAMCOI010000043.1	proteasome subunit alpha2 (EC 3.4.25.1)		K02726	-
JAMCOI010000043.1	[NU+] prion formation protein 1	COG0488	K03235	-
JAMCOI010000043.1	hypothetical protein	COG1457	-	-
JAMCOI010000043.1	hypothetical protein	COG0819	-	-
JAMCOI010000044.1	GDP-mannose 4,6 dehydratase	COG0451	K17741	-
JAMCOI010000044.1	Chorismate mutase III (EC 5.4.99.5)	COG1605	K01850	-
JAMCOI010000044.1	Ammonium Transporter Family	COG0004	K03320	-
JAMCOI010000044.1	hypothetical protein		K15109	-
JAMCOI010000044.1	hypothetical protein	COG4886	-	-

JAMCOI010000044.1	hypothetical protein	COG2608	K04569	-
JAMCOI010000045.1	hypothetical protein		K18183,K20406	-
JAMCOI010000045.1	5'-3' exoribonuclease 2	COG5049	K12618	-
JAMCOI010000046.1	Succinate dehydrogenase flavin-adding protein, antitoxin of CptAB toxin-antitoxin	COG2938	K18168,K20352	-
JAMCOI010000046.1	hypothetical protein	COG5126	K19932	-
JAMCOI010000046.1	hypothetical protein	COG0666	K06648	-
JAMCOI010000046.1	hypothetical protein	COG5246	K12826	-
JAMCOI010000046.1	Flavin adenine dinucleotide	COG0175	K00953	-
JAMCOI010000046.1	Flavin adenine dinucleotide	COG0175	K00953	-
JAMCOI010000046.1	hypothetical protein		K19996	-
JAMCOI010000046.1	hypothetical protein		K20456	-
JAMCOI010000046.1	hypothetical protein		K09240	-
JAMCOI010000047.1	hypothetical protein		K17771	-
JAMCOI010000047.1	hypothetical protein		K15115	-
JAMCOI010000047.1	Lysyl-tRNA synthetase (class II) (EC 6.1.1.6)	COG1190	K04567	-
JAMCOI010000047.1	6-phosphofructokinase, fungal/animal type (EC 2.7.1.11)	COG0205	K00850	-
JAMCOI010000047.1	RuvB-like DNA helicase RVB2 implicated in DNA remodeling, transcription initiation and RNA processing	COG1224	K11338	-
JAMCOI010000047.1	V-type ATP synthase subunit K (EC 3.6.3.14)	COG0636	K02155	-
JAMCOI010000047.1	hypothetical protein		K11561	-
JAMCOI010000047.1	hypothetical protein	COG5261	K05767	-
JAMCOI010000047.1	hypothetical protein	COG1985	K13886	-
JAMCOI010000047.1	Mannosyltransferase putative		-	-
JAMCOI010000048.1	54S ribosomal protein L31		-	-
JAMCOI010000048.1	hypothetical protein		K11557	-
JAMCOI010000048.1	hypothetical protein		K18171	-
JAMCOI010000048.1	Succinate dehydrogenase transmembrane subunit	COG2009	K00236	-
JAMCOI010000048.1	Oligosaccharyl transferase	COG1287	K07151	GT66
JAMCOI010000049.1	Ribosome assembly factor mrt4	COG0244	K14815	-
JAMCOI010000049.1	hypothetical protein		K11497	-
JAMCOI010000049.1	Free methionine-(R)-sulfoxide reductase, contains GAF domain	COG1956	K08968	-
JAMCOI010000050.1	hypothetical protein	COG2214	K09539	-
JAMCOI010000050.1	LSU ribosomal protein L24e-like protein	COG2075	K02896	-
JAMCOI010000050.1	hypothetical protein	COG5021	K10589	-
JAMCOI010000050.1	4-nitrophenylphosphatase	COG0647	K01101	-
JAMCOI010000050.1	hypothetical protein		K08197	-
JAMCOI010000050.1	hypothetical protein		K08197	-
JAMCOI010000050.1	Rpp20 subunit of nuclear RNase MRP and P		K14526	-
JAMCOI010000051.1	RNA-binding protein		K03258	-
JAMCOI010000051.1	Eukaryotic translation initiation factor 5A	COG0231	K03263	-
JAMCOI010000052.1	Phosphatidate cytidylyltransferase (EC 2.7.7.41)	COG0575	K00981	-
JAMCOI010000052.1	Translation initiation factor SUI1	COG0023	K03113	-
JAMCOI010000052.1	hypothetical protein		K02219	-
JAMCOI010000052.1	hypothetical protein	COG5032	K02543,K06640	-
JAMCOI010000053.1	hypothetical protein	COG1132	-	-
JAMCOI010000053.1	Coenzyme F420-dependent N5,N10-methylene tetrahydromethanopterin reductase and related flavin-dependent oxidoreductases	COG2141	K04091	-
JAMCOI010000053.1	2OG-Fe(II) oxygenase superfamily	COG3491	-	-
JAMCOI010000053.1	hypothetical protein		K11559	-
JAMCOI010000053.1	sulfurtransferase	COG2897	K01011	-
JAMCOI010000053.1	Sphingomyelin phosphodiesterase	COG0363	K01057	-
JAMCOI010000054.1	hypothetical protein		K01117	-
JAMCOI010000054.1	Multidrug efflux transporter MdtK/NorM (MATE family)	COG0534	K03327	-
JAMCOI010000054.1	hypothetical protein		K08343	-
JAMCOI010000054.1	Phospholipid:diacylglycerol acyltransferase (EC 2.3.1.158)		K00679	-
JAMCOI010000055.1	Kinase (Bud32/PRPK), required for threonylcarbamoyladenosine t(6)A37 formation in tRNA (p53-regulating)	COG3642	K08851	-
JAMCOI010000055.1	hypothetical protein		K12864	-
JAMCOI010000055.1	hypothetical protein	COG0706	K17797	-
JAMCOI010000055.1	hypothetical protein	COG5204	K15171	-
JAMCOI010000055.1	Cell division-associated, ATP-dependent zinc metalloprotease FtsH	COG0554	-	-
JAMCOI010000055.1	Methionyl-tRNA synthetase (EC 6.1.1.10)	COG0143	K01874	-
JAMCOI010000056.1	Transcription elongator complex protein 4		K11375	-
JAMCOI010000056.1	Meiotic recombination protein DMC1	COG0468	K10872,K14545	-
JAMCOI010000056.1	DNA replication licensing factor MCM4	COG1241	K02212	-
JAMCOI010000056.1	hypothetical protein		K20305	-
JAMCOI010000057.1	hypothetical protein	COG1026	-	-
JAMCOI010000057.1	hypothetical protein	COG5076	K11321	-

JAMCOI010000057.1	hypothetical protein		K17777	-
JAMCOI010000057.1	Asparaginyl-tRNA synthetase (EC 6.1.1.22)	COG0017	K01893	-
JAMCOI010000057.1	Histidinol-phosphatase (EC 3.1.3.15)	COG1387	K04486	-
JAMCOI010000057.1	Alcohol dehydrogenase (EC 1.1.1.1)	COG1064	K13953	-
JAMCOI010000058.1	D-aminoacyl-tRNA deacylase (EC 3.1.1.96)	COG1490	K07560	-
JAMCOI010000058.1	UDP-glucose:Glycoprotein Glucosyltransferase		K11718	GT24
JAMCOI010000058.1	5,10-methylenetetrahydrofolate reductase (EC 1.5.1.20)	COG0685	K00297	-
JAMCOI010000058.1	hypothetical protein Belongs to the ABC transporter superfamily	COG1131	K08711,K08712	-
JAMCOI010000059.1	hypothetical protein	COG1025	K01422	-
JAMCOI010000060.1	hypothetical protein		K18752	-
JAMCOI010000060.1	hypothetical protein		K03861	-
JAMCOI010000060.1	hypothetical protein		K14825	-
JAMCOI010000060.1	Protein tyrosine kinase		K02214	-
JAMCOI010000060.1	RNA polymerase II	COG0202	K03027	-
JAMCOI010000060.1	Aminotransferase, class III	COG0160	-	-
JAMCOI010000061.1	LSU ribosomal protein L32e	COG1717	K02912	-
JAMCOI010000061.1	RNA polymerase II		K15138	-
JAMCOI010000062.1	hypothetical protein		K18172	-
JAMCOI010000062.1	proteasome subunit beta3 (EC 3.4.25.1)	COG0638	K02735	-
JAMCOI010000062.1	Putative helicase	COG4581	K11593,K12598	-
JAMCOI010000062.1	hypothetical protein	COG2939	K13289	-
JAMCOI010000063.1	hypothetical protein		K14796	-
JAMCOI010000063.1	Leucyl-tRNA synthetase (EC 6.1.1.4)	COG0495	K01869	-
JAMCOI010000063.1	hypothetical protein		K03361,K22071	-
JAMCOI010000064.1	hypothetical protein	COG5189	K19487	-
JAMCOI010000065.1	hypothetical protein	COG0457	K03350	-
JAMCOI010000065.1	hypothetical protein		K17086	-
JAMCOI010000066.1	hypothetical protein		K15443	-
JAMCOI010000066.1	Vacuolar bilirubin concentrating pump YCF1	COG1132	-	-
JAMCOI010000067.1	hypothetical protein		K13336	-
JAMCOI010000067.1	hypothetical protein	COG5201	K03094	-
JAMCOI010000067.1	hypothetical protein		K00006	-
JAMCOI010000067.1	serine threonine protein kinase		K08286	-
JAMCOI010000068.1	hypothetical protein		K08052	-
JAMCOI010000068.1	hypothetical protein	COG0847	K18327	-
JAMCOI010000069.1	hypothetical protein		K05538	GT71
JAMCOI010000070.1	hypothetical protein	COG5636	-	-
JAMCOI010000070.1	Beta-mannosidase (EC 3.2.1.25)	COG3250	K01192	-
JAMCOI010000070.1	hypothetical protein	COG5185	K11547	-
JAMCOI010000071.1	hypothetical protein	COG0154	K01426	-
JAMCOI010000071.1	hypothetical protein	COG0477	K03448	-
JAMCOI010000071.1	hypothetical protein		K05535	GT71
JAMCOI010000072.1	Ribosomal L32p protein family	COG0333	K02911	-
JAMCOI010000072.1	Pyridoxamine 5'-phosphate oxidase (EC 1.4.3.5)	COG0259	K00275	-
JAMCOI010000073.1	hypothetical protein	COG1052	-	-
JAMCOI010000073.1	Xylulose kinase (EC 2.7.1.17)	COG1070	K00854	-
JAMCOI010000073.1	Saccharopine dehydrogenase [NAD+, L-lysine-forming] (EC 1.5.1.7)	COG1748	K00290,K11874	-
JAMCOI010000073.1	hypothetical protein		K14830	-
JAMCOI010000073.1	hypothetical protein		K03353	-
JAMCOI010000074.1	hypothetical protein		K19940	-
JAMCOI010000075.1	hypothetical protein		K14297,K18720	-
JAMCOI010000075.1	Cytosolic Fe-S cluster assembling factor NBP35	COG0489	K03593	-
JAMCOI010000075.1	hypothetical protein Belongs to the SNF7 family		K15053	-
JAMCOI010000075.1	hypothetical protein		K08332	-
JAMCOI010000076.1	hypothetical protein		K11215	-
JAMCOI010000076.1	hypothetical protein		K16578	-
JAMCOI010000076.1	transamidase	COG5206	K05290	-
JAMCOI010000077.1	hypothetical protein		K14555	-
JAMCOI010000077.1	hypothetical protein	COG0506	K00286,K00318,K	-
JAMCOI010000077.1	hypothetical protein		K06661,K15322	-
JAMCOI010000078.1	Lysine decarboxylase family	COG1611	-	-
JAMCOI010000078.1	Carbonic anhydrase, beta class (EC 4.2.1.1)	COG0288	K01673	-
JAMCOI010000078.1	SNF2 family N-terminal domain	COG0553	K19001	-
JAMCOI010000079.1	hypothetical protein	COG3104	K03305	-
JAMCOI010000080.1	hypothetical protein		K14411	-
JAMCOI010000080.1	Putative ATP-dependent RNA helicase	COG0513	K14808	-
JAMCOI010000081.1	hypothetical protein		K22493	-
JAMCOI010000082.1	Alpha-glucosidase (EC 3.2.1.20)	COG1501	K01187	GH31
JAMCOI010000083.1	hypothetical protein	COG5153	K17900	-

JAMCOI010000083.1	Pyridoxal kinase (EC 2.7.1.35)	COG2240	K00868	-
JAMCOI010000083.1	hypothetical protein		K14003	-
JAMCOI010000084.1	hypothetical protein		K17666	-
JAMCOI010000084.1	hypothetical protein	COG5099	K17943,K17944	-
JAMCOI010000084.1	hypothetical protein	COG1052	-	-
JAMCOI010000085.1	hypothetical protein		K18178	-
JAMCOI010000085.1	Thioredoxin	COG0526	K09584	-
JAMCOI010000085.1	phosphatidylinositol-4-phosphate 5-kinase	COG5253	K00889	-
JAMCOI010000085.1	hypothetical protein		K11344	-
JAMCOI010000085.1	hypothetical protein	COG1723	-	-
JAMCOI010000085.1	hypothetical protein		K14772	-
JAMCOI010000085.1	hypothetical protein	COG5648	K11680	-
JAMCOI010000085.1	Predicted cell-wall-anchored protein SasA (LPXTG motif)		-	-
JAMCOI010000085.1	hypothetical protein	COG0076	K01580,K14790	-
JAMCOI010000085.1	High-affinity carbon uptake protein Hat/HatR		K14855	-
JAMCOI010000085.1	hypothetical protein		K17430	-
JAMCOI010000085.1	Serine/Threonine protein kinase		K11230	-
JAMCOI010000085.1	hypothetical protein		K13113	-
JAMCOI010000085.1	Serine/threonine protein phosphatase (EC 3.1.3.16)	COG0639	K17615	-
JAMCOI010000085.1	hypothetical protein		K16362	-
JAMCOI010000085.1	N-acetylornithine aminotransferase (EC 2.6.1.11)	COG4992	K00818	-
JAMCOI010000085.1	hypothetical protein		K15163	-
JAMCOI010000085.1	hypothetical protein	COG0328	K03469	-
JAMCOI010000085.1	hypothetical protein	COG0328	K03469	-
JAMCOI010000085.1	phosphatidylinositol-4-phosphate 5-kinase	COG5253	K00889	-
JAMCOI010000085.1	Uncharacterized MFS-type transporter	COG0477	-	-
JAMCOI010000085.1	hypothetical protein		K12830	-
JAMCOI010000085.1	hypothetical protein	COG5025	-	-
JAMCOI010000085.1	WD repeat protein		K14961	-
JAMCOI010000086.1	Protein tyrosine kinase		K21157	-
JAMCOI010000086.1	Deacetylases, including yeast histone deacetylase and acetoin utilization protein	COG0123	K11404,K11483	-
JAMCOI010000086.1	proteasome subunit alpha1 (EC 3.4.25.1)	COG0638	K02730	-
JAMCOI010000086.1	hypothetical protein		K00223	-
JAMCOI010000087.1	hypothetical protein	COG1226	-	-
JAMCOI010000088.1	hypothetical protein	COG5274	-	-
JAMCOI010000088.1	hypothetical protein	COG5159	K12176	-
JAMCOI010000089.1	hypothetical protein		K14528	-
JAMCOI010000090.1	Glucose-6-phosphate isomerase (EC 5.3.1.9)	COG0166	K01810	-
JAMCOI010000090.1	Acetyltransferase (GNAT) domain		-	-
JAMCOI010000091.1	Dihydroxyacetone kinase, ATP-dependent (EC 2.7.1.29)	COG2376	K00863	-
JAMCOI010000091.1	hypothetical protein		K09246	-
JAMCOI010000092.1	Replication factor C small subunit	COG0470	K10756	-
JAMCOI010000093.1	hypothetical protein		K03233	-
JAMCOI010000093.1	hypothetical protein		K15032	-
JAMCOI010000093.1	Cyclin		K06656,K06657	-
JAMCOI010000094.1	hypothetical protein		K08284	-
JAMCOI010000095.1	hypothetical protein		K03233	-
JAMCOI010000095.1	hypothetical protein		K05529	GT62
JAMCOI010000095.1	OBG-family ribosome assembly protein NOG1/MJ1408	COG1084	K06943	-
JAMCOI010000095.1	hypothetical protein		K14404	-
JAMCOI010000097.1	glycerophosphoinositol permease		-	-
JAMCOI010000097.1	glycerophosphoinositol permease		-	-
JAMCOI010000097.1	glycerophosphoinositol permease		-	-
JAMCOI010000099.1	hypothetical protein		K20347	-
JAMCOI010000099.1	hypothetical protein		K12763	-
JAMCOI010000100.1	hypothetical protein		K11363	-
JAMCOI010000100.1	hypothetical protein		K15326	-
JAMCOI010000100.1	hypothetical protein	COG2273	K21850	GH16
JAMCOI010000101.1	3-oxoacyl-CoA synthase		-	-
JAMCOI010000102.1	hypothetical protein		K01183	GH18
JAMCOI010000102.1	Inositol oxygenase (EC 1.13.99.1)		K00469	-
JAMCOI010000102.1	hypothetical protein	COG5309	-	-
JAMCOI010000103.1	Glycine dehydrogenase [decarboxylating] (glycine cleavage system P protein) (EC 1.4.4.2)	COG1003	K00281	-
JAMCOI010000103.1	hypothetical protein		K14556	-
JAMCOI010000103.1	hypothetical protein		K15158	-
JAMCOI010000103.1	hypothetical protein		K15158	-
JAMCOI010000103.1	Low molecular weight protein tyrosine phosphatase (EC 3.1.3.48)	COG0394	K14394	-

JAMCOI010000103.1	hypothetical protein	COG5665	K12580	-
JAMCOI010000103.1	hypothetical protein		K19850	-
JAMCOI010000103.1	Transketolase (EC 2.2.1.1)	COG0021	K00615	-
JAMCOI010000103.1	Ca(2+)/H(+) antiporter @ Vacuolar	COG0387	K07300	-
JAMCOI010000103.1	hypothetical protein		K06657	-
JAMCOI010000103.1	Thermosome subunit	COG0459	K09493	-
JAMCOI010000103.1	Protein kinase		K13303	-
JAMCOI010000103.1	LSU ribosomal protein L40mt, mitochondrial		K17421	-
JAMCOI010000103.1	hypothetical protein		K11344	-
JAMCOI010000103.1	Enolase (EC 4.2.1.11)	COG0148	K01689	-
JAMCOI010000103.1	Kynureninase (EC 3.7.1.3)	COG3844	K01556	-
JAMCOI010000103.1	hypothetical protein	COG5030	K12399	-
JAMCOI010000103.1	Cell division control protein 42	COG1100	K04393	-
JAMCOI010000103.1	hypothetical protein	COG4586	K12608	-
JAMCOI010000103.1	hypothetical protein		K12882	-
JAMCOI010000103.1	Homocysteine S-methyltransferase (EC 2.1.1.10)	COG0646	K00547	-
JAMCOI010000103.1	Phospholipase B		K13333	-
JAMCOI010000103.1	hypothetical protein	COG5239	K12603	-
JAMCOI010000103.1	hypothetical protein	COG5239	K12603	-
JAMCOI010000103.1	hypothetical protein	COG1958	K11086,K22138	-
JAMCOI010000103.1	GTP-binding protein YPT31/YPT8	COG1100	K07904,K07905	-
JAMCOI010000104.1	hypothetical protein		K02153	-
JAMCOI010000104.1	hypothetical protein belonging to Vps23 core domain		K12183	-
JAMCOI010000105.1	proteasome subunit alpha4 (EC 3.4.25.1)	COG0638	K02731	-
JAMCOI010000105.1	Flap structure-specific endonuclease	COG0258	K04799	-
JAMCOI010000105.1	hypothetical protein belonging to Septin GTPase family	COG5019	K16938	-
JAMCOI010000106.1	Multidrug efflux transporter MdtK/NorM (MATE family)	COG0534	K03327	-
JAMCOI010000106.1	hypothetical protein		K12870	-
JAMCOI010000106.1	Nicotinamidase (EC 3.5.1.19)	COG1335	K01440	-
JAMCOI010000107.1	hypothetical protein		K05538	GT71
JAMCOI010000107.1	hypothetical protein		K05538	GT71
JAMCOI010000109.1	hypothetical protein	COG5210	K18469	-
JAMCOI010000110.1	hypothetical protein		K05538	GT71
JAMCOI010000110.1	hypothetical protein		K05538	GT71
JAMCOI010000111.1	NADH-ubiquinone oxidoreductase chain G (EC 1.6.5.3)	COG1034	K03934	-
JAMCOI010000111.1	hypothetical protein	COG5182	K12829	-
JAMCOI010000111.1	hypothetical protein	COG5599	K01104,K19806	-
JAMCOI010000111.1	Thioredoxin	COG0526	K03671	-
JAMCOI010000111.1	hypothetical protein		K18804	-
JAMCOI010000111.1	hypothetical protein		K03245	-
JAMCOI010000111.1	hypothetical protein	COG5128	K20280	-
JAMCOI010000111.1	Peptide alpha-N-acetyltransferase ARD1 subunit (EC 2.3.1.88)	COG0456	K20791	-
JAMCOI010000111.1	Putative RNA-binding protein COG1094	COG1094	K11884	-
JAMCOI010000111.1	hypothetical protein		K17980	-
JAMCOI010000111.1	hypothetical protein	COG5275	K11134,K17922	-
JAMCOI010000111.1	Transcriptional regulator		K11313,K19476	-
JAMCOI010000111.1	hypothetical protein		K14782	-
JAMCOI010000111.1	Eukaryotic translation initiation factor 4E	COG5053	K03259	-
JAMCOI010000111.1	Protein bem46	COG1073	K06889	-
JAMCOI010000111.1	hypothetical protein		K20408	-
JAMCOI010000111.1	hypothetical protein	COG5086	K10735	-
JAMCOI010000111.1	hypothetical protein		K10415	-
JAMCOI010000111.1	hypothetical protein		K06670	-
JAMCOI010000111.1	hypothetical protein	COG0355	K02134	-
JAMCOI010000111.1	hypothetical protein	COG1063	K00008,K05351	-
JAMCOI010000111.1	hypothetical protein		K17438	-
JAMCOI010000111.1	hypothetical protein	COG1958	K11087	-
JAMCOI010000111.1	hypothetical protein	COG5291	K13989	-
JAMCOI010000111.1	Cation/H+ antiporter @ Vacuolar	COG0387	K07300	-
JAMCOI010000111.1	Oligo-1,6-glucosidase (EC 3.2.1.10)	COG0366	K01182,K01187	GH13,GH31
JAMCOI010000111.1	hypothetical protein		K12562	-
JAMCOI010000111.1	GDSL-like Lipase/Acylhydrolase family	COG0214	-	-
JAMCOI010000111.1	Glutamate 5-kinase (EC 2.7.2.11) / RNA-binding C-terminal domain PUA	COG0263	K00931	-
JAMCOI010000111.1	hypothetical protein		K20177	-
JAMCOI010000111.1	4-hydroxybenzoate polyprenyltransferase (EC 2.5.1.39)	COG0382	K06125	-
JAMCOI010000111.1	hypothetical protein	COG5194	K03358	-
JAMCOI010000111.1	Biotin--protein ligase (EC 6.3.4.9)(EC 6.3.4.10)(EC 6.3.4.11)(EC 6.3.4.15)	COG0340	K01942	-

JAMCOI010000111.1	Multi antimicrobial extrusion protein (Na(+)/drug antiporter), MATE family of MDR efflux pumps	COG0534	K03327	-
JAMCOI010000111.1	hypothetical protein		K05012,K14798	-
JAMCOI010000111.1	hypothetical protein		K03097	-
JAMCOI010000112.1	WD repeat protein		K11805	-
JAMCOI010000117.1	Seryl-tRNA synthetase (EC 6.1.1.11)	COG0172	K01875	-
JAMCOI010000117.1	hypothetical protein		K10570	-
JAMCOI010000117.1	hypothetical protein		K06687	-
JAMCOI010000117.1	hypothetical protein		K14213	-
JAMCOI010000117.1	hypothetical protein		K20300	-
JAMCOI010000117.1	Enoyl-(Acyl carrier protein) reductase	COG1028	K00059	-
JAMCOI010000117.1	Chromosome partition protein smc	COG1196	K06669	-
JAMCOI010000117.1	Dolichol kinase (EC 2.7.1.108)	COG0170	K00902,K05542	-
JAMCOI010000117.1	Fatty acid desaturase	COG3239	K13076	-
JAMCOI010000117.1	hypothetical protein		K14853	-
JAMCOI010000117.1	hypothetical protein		K22138	-
JAMCOI010000117.1	Intracellular protease	COG0693	K22211	-
JAMCOI010000117.1	Ribosomal RNA processing endonuclease Nob1	COG1439	K11883	-
JAMCOI010000117.1	hypothetical protein	COG2273	K21850	GH16
JAMCOI010000117.1	hypothetical protein	COG0666	K06694	-
JAMCOI010000117.1	Prefoldin subunit 3		-	-
JAMCOI010000117.1	LSU ribosomal protein L18e	COG1727	K02883	-
JAMCOI010000117.1	LSU ribosomal protein L18e	COG1727	K02883	-
JAMCOI010000117.1	Alcohol dehydrogenase	COG0604	-	-
JAMCOI010000117.1	3-oxoacyl-[acyl-carrier-protein] synthase, KASII (EC 2.3.1.179)	COG0304	K09458	-
JAMCOI010000117.1	Integral membrane protein		K17907	-
JAMCOI010000117.1	Uncharacterized MFS-type transporter	COG0477	K08192	-
JAMCOI010000117.1	hypothetical protein Belongs to the ABC transporter superfamily	COG1131	K08711,K08712	-
JAMCOI010000120.1	serine threonine protein kinase		K08286	-
JAMCOI010000121.1	hypothetical protein	COG0494	K03574,K17816	-
JAMCOI010000121.1	Putative oxidoreductase	COG0667	K17647	-
JAMCOI010000124.1	hypothetical protein		K13682	GT91
JAMCOI010000124.1	Carbon-nitrogen hydrolase	COG0388	K14663	-
JAMCOI010000124.1	4-aminobutyrate aminotransferase (EC 2.6.1.19)	COG0160	K13524	-
JAMCOI010000124.1	hypothetical protein	COG1131	K08712	-
JAMCOI010000124.1	hypothetical protein	COG0389	K03509	-
JAMCOI010000124.1	hypothetical protein		K14327	-
JAMCOI010000124.1	hypothetical protein	COG1881	K17439	-
JAMCOI010000124.1	hypothetical protein	COG1881	K17439	-
JAMCOI010000124.1	hypothetical protein		K09043	-
JAMCOI010000124.1	hypothetical protein		K02264	-
JAMCOI010000124.1	Cytochrome c oxidase polypeptide Va, mitochondrial precursor (EC 1.9.3.1)		K02264	-
JAMCOI010000124.1	hypothetical protein	COG5034	K11319,K11396	-
JAMCOI010000124.1	hypothetical protein		K21991	-
JAMCOI010000124.1	hypothetical protein	COG5028	K14007	-
JAMCOI010000124.1	Chaperone protein ClpB (ATP-dependent unfoldase)	COG0542	K01509,K03695	-
JAMCOI010000124.1	hypothetical protein		K20047	-
JAMCOI010000124.1	Hydroxymethylpyrimidine phosphate kinase ThiD (EC 2.7.4.7)	COG0351	K00877	-
JAMCOI010000125.1	Oligo-1,6-glucosidase (EC 3.2.1.10)	COG0366	K01182,K01187	GH13,GH31
JAMCOI010000126.1	Ferric reductase		-	-
JAMCOI010000130.1	hypothetical protein		K19996	-
JAMCOI010000130.1	hypothetical protein		K03003	-
JAMCOI010000130.1	hypothetical protein	COG0684	-	-
JAMCOI010000130.1	hypothetical protein	COG5369	-	-
JAMCOI010000130.1	hypothetical protein		K04345	-
JAMCOI010000130.1	Protein tyrosine kinase		K08286	-
JAMCOI010000130.1	serine threonine-protein kinase		K08286,K19833	-
JAMCOI010000130.1	hypothetical protein		K12881	-
JAMCOI010000130.1	hypothetical protein		K20060	-
JAMCOI010000130.1	Methyltransferase domain	COG0500	K22438	-
JAMCOI010000130.1	hypothetical protein		K05538	GT71
JAMCOI010000130.1	hypothetical protein	COG2120	K03434	-
JAMCOI010000130.1	hypothetical protein Belongs to the actin family		K11662	-
JAMCOI010000130.1	hypothetical protein		K20059	-
JAMCOI010000130.1	LSU ribosomal protein L44e	COG1631	K02929	-
JAMCOI010000130.1	LSU ribosomal protein L44e	COG1631	K02929	-
JAMCOI010000130.1	LSU ribosomal protein L44e	COG1631	K02929	-
JAMCOI010000130.1	Serine threonine-protein kinase		K08286	-

JAMCOI010000130.1	Uncharacterized membrane protein YCR061W		-	-
JAMCOI010000135.1	Pyridine nucleotide-disulphide oxidoreductase	COG2072	K03379	-
JAMCOI010000136.1	Acetyltransferase	COG0110	-	-
JAMCOI010000136.1	ubiquitin-activating E1 family	COG0476	K03178	-
JAMCOI010000136.1	Lipid A export ATP-binding/permease protein MsbA	COG1132	K05658	-
JAMCOI010000136.1	hypothetical protein	COG5085	-	-
JAMCOI010000136.1	hypothetical protein Uracil DNA glycosylase superfamily	COG3663	K20813	-
JAMCOI010000136.1	Saccharopine dehydrogenase [NADP, L-glutamate-forming] (EC 1.5.1.10)	COG1748	K00293,K00797	-
JAMCOI010000136.1	hypothetical protein	COG0477	K08178	-
JAMCOI010000136.1	Phosphatidyl-N-methylethanolamine N-methyltransferase (EC 2.1.1.17)(EC 2.1.1.71)		K00551	-
JAMCOI010000136.1	hypothetical protein	COG5073	-	-
JAMCOI010000136.1	hypothetical protein Uracil DNA glycosylase	COG3663	K20813	-
JAMCOI010000136.1	hypothetical protein		K13682	GT91
JAMCOI010000136.1	hypothetical protein	COG0515	K08286	-
JAMCOI010000136.1	hypothetical protein		K10731	-
JAMCOI010000136.1	hypothetical protein	COG5095	K03131	-
JAMCOI010000136.1	Vacuolar cation-chloride cotransporter	COG0531	K14429	-
JAMCOI010000136.1	hypothetical protein	COG0513	K12811	-
JAMCOI010000136.1	enoyl-CoA hydratase		K12663	-
JAMCOI010000136.1	hypothetical protein		K17672	-
JAMCOI010000136.1	hypothetical protein		K14849	-
JAMCOI010000136.1	hypothetical protein		K12819	-
JAMCOI010000136.1	hypothetical protein		K18757	-
JAMCOI010000136.1	hypothetical protein		K17822	-
JAMCOI010000136.1	hypothetical protein		K03349	-
JAMCOI010000136.1	hypothetical protein	COG5624	K03126	-
JAMCOI010000137.1	hypothetical protein		K08141	-
JAMCOI010000139.1	hypothetical protein		K19996	-
JAMCOI010000139.1	hypothetical protein		K15102	-
JAMCOI010000139.1	hypothetical protein	COG5037	K21631	-
JAMCOI010000139.1	hypothetical protein	COG5174	K03137	-
JAMCOI010000139.1	hypothetical protein	COG5174	K03137	-
JAMCOI010000139.1	Mannosyltransferase putative		K05535	GT71
JAMCOI010000139.1	hypothetical protein	COG5061	-	-
JAMCOI010000139.1	DEAD-box ATP-dependent RNA helicase DeaD (= CshA) (EC 3.6.4.13)	COG0513	K14811	-
JAMCOI010000139.1	hypothetical protein	COG5641	-	-
JAMCOI010000139.1	Protein tyrosine kinase		K03114	-
JAMCOI010000139.1	hypothetical protein	COG2453	K11240,K19812	-
JAMCOI010000139.1	hypothetical protein	COG5329	K21797	-
JAMCOI010000139.1	hypothetical protein	COG2267	K01054	-
JAMCOI010000139.1	hypothetical protein		K18187	-
JAMCOI010000139.1	hypothetical protein		K20184	-
JAMCOI010000140.1	Aldehyde dehydrogenase (EC 1.2.1.3)	COG1012	K00128,K00129,K	-
JAMCOI010000140.1	hypothetical protein		K15183	-
JAMCOI010000140.1	hypothetical protein	COG5277	K11673	-
JAMCOI010000140.1	Succinyl-CoA ligase [ADP-forming] alpha chain (EC 6.2.1.5)	COG0074	K01899	-
JAMCOI010000140.1	hypothetical protein	COG5155	K02365	-
JAMCOI010000140.1	hypothetical protein		K18550	-
JAMCOI010000140.1	SSU ribosomal protein S4e	COG1471	K02987	-
JAMCOI010000140.1	hypothetical protein		K18159	-
JAMCOI010000140.1	hypothetical protein	COG1028	K00218,K11153	-
JAMCOI010000140.1	hypothetical protein		K17279	-
JAMCOI010000140.1	LSU ribosomal protein L23p (L23Ae), mitochondrial		K02892	-
JAMCOI010000140.1	DNA-directed RNA polymerase II second largest subunit (EC 2.7.7.6)	COG0085	K03010	-
JAMCOI010000140.1	LSU ribosomal protein L13p (L13Ae)	COG0102	K02871	-
JAMCOI010000140.1	DNA-directed RNA polymerase II 19 kDa polypeptide (EC 2.7.7.6)	COG1095	K03015	-
JAMCOI010000140.1	hypothetical protein		K04706,K20304	-
JAMCOI010000140.1	hypothetical protein	COG5391	-	-
JAMCOI010000140.1	Arginyl-tRNA synthetase (EC 6.1.1.19)	COG0018	K01887	-
JAMCOI010000140.1	Superoxide dismutase [Mn] (EC 1.15.1.1)	COG0605	K04564	-
JAMCOI010000140.1	SSU ribosomal protein S2e (S5p)	COG0098	K02981	-
JAMCOI010000140.1	hypothetical protein	COG0661	K08869	-
JAMCOI010000140.1	GTP-binding protein RHO1	COG1100	K07975	-
JAMCOI010000140.1	putative membrane-associated phospholipid phosphatase, PAP2 superfamily	COG0671	K04716,K04717	-
JAMCOI010000140.1	hypothetical protein		K19815	-

JAMCOI010000140.1	tRNA (uracil(54)-C5)-methyltransferase (EC 2.1.1.35) @ tmRNA (uracil(341)-C5)-methyltransferase	COG2265	K15331	-
JAMCOI010000140.1	Glycosyl transferase	COG5597	K00750	GT8
JAMCOI010000140.1	Eukaryotic translation initiation factor 4A	COG0513	K03257	-
JAMCOI010000140.1	Deoxyuridine 5'-triphosphate nucleotidohydrolase (EC 3.6.1.23)	COG0756	K01520	-
JAMCOI010000140.1	hypothetical protein		K17800	-
JAMCOI010000140.1	hypothetical protein		K03671	-
JAMCOI010000140.1	RNA polymerase II		K15140	-
JAMCOI010000140.1	hypothetical protein	COG5603	K20301	-
JAMCOI010000140.1	Putative oxidoreductase	COG0667	K17647	-
JAMCOI010000140.1	hypothetical protein	COG2072	-	-
JAMCOI010000140.1	hypothetical protein		K12778	-
JAMCOI010000140.1	hypothetical protein	COG1097	K03681	-
JAMCOI010000140.1	tRNA(Phe) (7-(3-amino-3-(methoxycarbonyl)propyl)wyosine(37)-N)-methoxycarbonyltransferase (EC 2.3.1.231) @ tRNA(Phe) (7-(3-amino-3-carboxypropyl)wyosine(37)-O)-methyltransferase (EC 2.1.1.290)		K15451	-
JAMCOI010000140.1	hypothetical protein		K10259	-
JAMCOI010000140.1	Zinc finger protein ZPR1	COG1779	K06874	-
JAMCOI010000140.1	hypothetical protein		K21864	-
JAMCOI010000140.1	hypothetical protein		K17411	-
JAMCOI010000140.1	hypothetical protein		K11560	-
JAMCOI010000140.1	hypothetical protein	COG1813	K03627	-
JAMCOI010000140.1	3-oxo-5-alpha-steroid 4-dehydrogenase		K10258	-
JAMCOI010000140.1	Peptide-methionine (R)-S-oxide reductase MsrB (EC 1.8.4.12)	COG0229	K07305	-
JAMCOI010000140.1	Protein arginine N-methyltransferase 1 (EC 2.1.1.-)	COG0500	K11434	-
JAMCOI010000140.1	hypothetical protein		K03937	-
JAMCOI010000140.1	hypothetical protein		K02608	-
JAMCOI010000140.1	Probable serine protease do-like precursor	COG0265	-	-
JAMCOI010000140.1	hypothetical protein ribosomal protein bL35 family	COG0291	-	-
JAMCOI010000140.1	hypothetical protein	COG2940	K11422	-
JAMCOI010000140.1	hypothetical protein		K06631,K06660	-
JAMCOI010000140.1	DNA-directed RNA polymerase II 13.3 kDa polypeptide (EC 2.7.7.6)	COG1761	K03008	-
JAMCOI010000140.1	hypothetical protein	COG5602	K11644	-
JAMCOI010000140.1	hypothetical protein	COG5602	K11644	-
JAMCOI010000140.1	hypothetical protein	COG5531	K15223	-
JAMCOI010000140.1	hypothetical protein	COG5225	K14852	-
JAMCOI010000140.1	hypothetical protein	COG1100	K07870	-
JAMCOI010000141.1	Haloacid dehalogenase-like hydrolase	COG1011	K07025,K18551	-
JAMCOI010000145.1	Sphingomyelin phosphodiesterase		K01117	-
JAMCOI010000147.1	Oxidoreductase, short-chain dehydrogenase/reductase family	COG1028	-	-
JAMCOI010000147.1	Alpha/beta hydrolase family	COG2267	K01054	-
JAMCOI010000147.1	Glutamate N-acetyltransferase (EC 2.3.1.35) @ N-acetylglutamate synthase (EC 2.3.1.1)	COG1364	K00620	-
JAMCOI010000147.1	hypothetical protein	COG5108	K10908	-
JAMCOI010000147.1	hypothetical protein		K20304	-
JAMCOI010000147.1	RNA-binding protein		-	-
JAMCOI010000147.1	Protein tyrosine kinase		K00871	-
JAMCOI010000147.1	Glutaminyl-tRNA synthetase (EC 6.1.1.18)	COG0008	K01886	-
JAMCOI010000147.1	tRNA(His) guanylyltransferase (EC 2.7.7.79)	COG4021	K10761	-
JAMCOI010000147.1	DNA-directed RNA polymerase I second largest subunit (EC 2.7.7.6)	COG0085	K03002	-
JAMCOI010000147.1	Pantothenate kinase (EC 2.7.1.33)	COG1072	-	-
JAMCOI010000147.1	LSU ribosomal protein L5e (L18p)	COG0256	K02932	-
JAMCOI010000147.1	Iron-sulfur cluster assembly scaffold protein IscU	COG0822	K22068	-
JAMCOI010000147.1	hypothetical protein		K15217	-
JAMCOI010000148.1	N-acetylglucosamine-1-phosphate uridyltransferase eukaryotic (EC 2.7.7.23)	COG4284	K00972	-
JAMCOI010000148.1	hypothetical protein	COG5019	K16945,K16947	-
JAMCOI010000148.1	hypothetical protein	COG5019	K16945,K16947	-
JAMCOI010000148.1	hypothetical protein	COG5213	K14405	-
JAMCOI010000148.1	Eukaryotic translation initiation factor 5	COG1601	K03262	-
JAMCOI010000148.1	hypothetical protein	COG4886	K18809	-
JAMCOI010000148.1	hypothetical protein	COG5099	K17974	-
JAMCOI010000148.1	hypothetical protein	COG5600	-	-
JAMCOI010000148.1	hypothetical protein		K03360	-
JAMCOI010000148.1	LSU ribosomal protein L37Ae	COG1997	K02921	-
JAMCOI010000148.1	hypothetical protein ESCRT-II complex subunit		K12189	-
JAMCOI010000148.1	CTP synthase (EC 6.3.4.2)	COG0504	K01937	-
JAMCOI010000148.1	Iron/manganese superoxide dismutase	COG0605	K04564	-
JAMCOI010000148.1	hypothetical protein		K17607	-

JAMCOI010000148.1	Glycerophosphoryl diester phosphodiesterase	COG0584	K18696	-
JAMCOI010000148.1	Arginase (EC 3.5.3.1)	COG0010	K01476	-
JAMCOI010000148.1	hypothetical protein		K15100	-
JAMCOI010000148.1	ubiquitin carboxyl-terminal hydrolase		K05610	-
JAMCOI010000148.1	hypothetical protein		K01166	-
JAMCOI010000148.1	Dienelactone hydrolase family	COG0412	-	-
JAMCOI010000148.1	Serine hydrolase (FSH1)		K00287	-
JAMCOI010000148.1	hypothetical protein	COG0526	K09584	-
JAMCOI010000148.1	Ferric reductase (1.6.99.14)		-	-
JAMCOI010000149.1	hypothetical protein		K14860	-
JAMCOI010000149.1	Ribulose-5-phosphate 4-epimerase and related epimerases and aldolases	COG0235	-	-
JAMCOI010000149.1	N-acetylglutamate kinase (EC 2.7.2.8) / N-acetyl-gamma-glutamyl-phosphate reductase (EC 1.2.1.38)	COG0002	K12659	-
JAMCOI010000149.1	Tyrosine phosphatase family	COG2365	-	-
JAMCOI010000149.1	hypothetical protein		K19992	-
JAMCOI010000149.1	hypothetical protein		K19730	-
JAMCOI010000149.1	Serine/threonine protein kinase		K18669,K18670	-
JAMCOI010000149.1	hypothetical protein	COG4178	K15628	-
JAMCOI010000149.1	Sphingoid long chain base kinase (EC 2.7.1.91)	COG1597	K04718	-
JAMCOI010000149.1	proteasome regulatory subunit Rpn2	COG5116	K03032	-
JAMCOI010000149.1	D-3-phosphoglycerate dehydrogenase (EC 1.1.1.95)	COG0111	K00058	-
JAMCOI010000149.1	hypothetical protein	COG5059	K10396	-
JAMCOI010000149.1	hypothetical protein		K15151	-
JAMCOI010000149.1	hypothetical protein		K19770	-
JAMCOI010000149.1	hypothetical protein	COG0599	-	-
JAMCOI010000149.1	LSU ribosomal protein L43mt, mitochondrial		K17424	-
JAMCOI010000149.1	hypothetical protein		K12609	-
JAMCOI010000149.1	hypothetical protein		K12199	-
JAMCOI010000149.1	hypothetical protein	COG5600	-	-
JAMCOI010000149.1	hypothetical protein		K14328	-
JAMCOI010000149.1	hypothetical protein	COG0599	-	-
JAMCOI010000149.1	hypothetical protein	COG4886	-	-
JAMCOI010000149.1	hypothetical protein	COG0814	K14209	-
JAMCOI010000150.1	Serine threonine-protein phosphatase	COG0639	K06269	-
JAMCOI010000150.1	hypothetical protein		K21544	-
JAMCOI010000150.1	hypothetical protein	COG1341	K06947	-
JAMCOI010000150.1	hypothetical protein	COG0625	K00799	-
JAMCOI010000150.1	hypothetical protein	COG5243	K10601	-
JAMCOI010000150.1	RNA polymerase I specific initiation factor		K15222	-
JAMCOI010000150.1	Heat shock protein 60 kDa family chaperone GroEL	COG0459	K04077	-
JAMCOI010000150.1	GTP-binding protein YPT6		K07893	-
JAMCOI010000150.1	hypothetical protein	COG2118	K06875	-
JAMCOI010000150.1	hypothetical protein	COG5059	K10398	-
JAMCOI010000150.1	3-oxoacyl-coenzyme A reductase of elongase (EC 1.1.1.62)	COG0300	K10251	-
JAMCOI010000150.1	Chaperone protein DnaJ	COG0484	K09510	-
JAMCOI010000150.1	hypothetical protein		K08266	-
JAMCOI010000150.1	hypothetical protein	COG2940	K11426	-
JAMCOI010000150.1	hypothetical protein		K09174,K09175	-
JAMCOI010000150.1	LSU ribosomal protein L7e (L30p)	COG1841	K02937	-
JAMCOI010000150.1	Adenylosuccinate lyase (EC 4.3.2.2) @ SAICAR lyase (EC 4.3.2.2)	COG0015	K01756	-
JAMCOI010000150.1	hypothetical protein		K11568	-
JAMCOI010000150.1	hypothetical protein		K05757	-
JAMCOI010000150.1	hypothetical protein	COG0053	-	-
JAMCOI010000151.1	hypothetical protein		K16362	-
JAMCOI010000151.1	hypothetical protein	COG0265	K06693	-
JAMCOI010000151.1	hypothetical protein	COG5131	K12161	-
JAMCOI010000151.1	hypothetical protein		K12670	-
JAMCOI010000151.1	hypothetical protein Belongs to the small GTPase superfamily. Arf family		K07952	-
JAMCOI010000151.1	hypothetical protein		K09043	-
JAMCOI010000151.1	hypothetical protein	COG5190	K17496	-
JAMCOI010000151.1	CDP-diacylglycerol--serine O-phosphatidyltransferase (EC 2.7.8.8)	COG1183	K17103	-
JAMCOI010000151.1	hypothetical protein	COG5217	K10436	-
JAMCOI010000151.1	2,4-dienoyl-CoA reductase [NADPH] (EC 1.3.1.34)	COG1902	-	-
JAMCOI010000151.1	hypothetical protein	COG0513	K12858	-
JAMCOI010000151.1	hypothetical protein	COG1774	-	-
JAMCOI010000151.1	hypothetical protein	COG5227	K12160	-
JAMCOI010000151.1	LSU ribosomal protein L37e	COG2126	K02922	-
JAMCOI010000151.1	hypothetical protein		K12578	-

JAMCOI010000151.1	rRNA small subunit pseudouridine methyltransferase Nep1 (EC 2.1.1.260)	COG1756	K14568	-
JAMCOI010000151.1	hypothetical protein		K02515,K06668,K	-
JAMCOI010000152.1	hypothetical protein		K11563	-
JAMCOI010000152.1	hypothetical protein	COG0814	-	-
JAMCOI010000152.1	hypothetical protein	COG5066	-	-
JAMCOI010000152.1	Methionine aminopeptidase (EC 3.4.11.18)	COG0024	K01265	-
JAMCOI010000152.1	Alpha-1,3-mannosyltransferase	COG0438	K03843	GT4
JAMCOI010000152.1	hypothetical protein		K03845	GT58
JAMCOI010000152.1	hypothetical protein		K14836	-
JAMCOI010000152.1	hypothetical protein		K13352	-
JAMCOI010000152.1	hypothetical protein		K19718	-
JAMCOI010000152.1	hypothetical protein Belongs to the ubiquitin-conjugating enzyme family	COG5078	K04649	-
JAMCOI010000152.1	hypothetical protein	COG5103	K12604	-
JAMCOI010000152.1	hypothetical protein		K02267	-
JAMCOI010000152.1	alanine glyoxylate aminotransferase	COG0075	K00830	-
JAMCOI010000152.1	Efflux ABC transporter, permease/ATP-binding protein mlr7818	COG5265	K05663	-
JAMCOI010000152.1	hypothetical protein	COG0308	-	-
JAMCOI010000152.1	Pantothenate kinase type II, eukaryotic (EC 2.7.1.33)	COG5146	K09680	-
JAMCOI010000152.1	Cystathionine beta-synthase (EC 4.2.1.22)	COG0031	K01738	-
JAMCOI010000153.1	Dihydrolipoamide acetyltransferase component of pyruvate dehydrogenase complex (EC 2.3.1.12)	COG0508	K00627	-
JAMCOI010000153.1	RNA polymerase III transcription initiation factor (TFIIIC) 55 kDa subunit		K15206	-
JAMCOI010000153.1	Phosphoglycerate mutase family	COG0406	K15206	-
JAMCOI010000153.1	hypothetical protein	COG5047	K14006	-
JAMCOI010000153.1	sulfhydryl oxidase	COG5054	K17783,K17891	-
JAMCOI010000153.1	hypothetical protein		K07973	-
JAMCOI010000153.1	Glutaredoxin	COG0695	K03676	-
JAMCOI010000153.1	3-beta hydroxysteroid dehydrogenase/isomerase family	COG0451	K17741	-
JAMCOI010000153.1	hypothetical protein		K12767	-
JAMCOI010000153.1	hypothetical protein	COG5296	K15178	-
JAMCOI010000153.1	hypothetical protein	COG5274	-	-
JAMCOI010000153.1	hypothetical protein		K14838	-
JAMCOI010000153.1	hypothetical protein (t-SNAREs or soluble NSF)		K08495	-
JAMCOI010000153.1	Glutamyl-tRNA synthetase (EC 6.1.1.17)	COG0008	K01885	-
JAMCOI010000153.1	hypothetical protein	COG1723	-	-
JAMCOI010000153.1	hypothetical protein		K17440	-
JAMCOI010000154.1	hypothetical protein (GTPase-activator protein for Rho-like GTPases)		K16733,K19839	-
JAMCOI010000154.1	hypothetical protein		K18167	-
JAMCOI010000154.1	hypothetical protein		K10365	-
JAMCOI010000154.1	Dicarboxylic amino acid permease	COG0531	K16261	-
JAMCOI010000154.1	Pyruvate decarboxylase (EC 4.1.1.1); Alpha-keto-acid decarboxylase (EC 4.1.1.-)	COG3961	K01568	-
JAMCOI010000154.1	Pyruvate decarboxylase (EC 4.1.1.1); Alpha-keto-acid decarboxylase (EC 4.1.1.-)	COG3961	K01568	-
JAMCOI010000154.1	Phosphomevalonate kinase (EC 2.7.4.2)	COG3890	K00938	-
JAMCOI010000154.1	Diacylglycerol pyrophosphate phosphatase (EC 3.1.3.81)	COG0671	K18693	-
JAMCOI010000154.1	Prephenate and/or arogenate dehydrogenase (unknown specificity) (EC 1.3.1.12)(EC 1.3.1.43)	COG0287	K00211	-
JAMCOI010000154.1	hypothetical protein	COG1100	K07942	-
JAMCOI010000154.1	Malate dehydrogenase (EC 1.1.1.37)	COG0039	K00026	-
JAMCOI010000154.1	RNA polymerase III transcription initiation factor complex		K15202	-
JAMCOI010000154.1	Dolichol-phosphate mannosyltransferase subunit 3 (DPM3)		K09659	GT2
JAMCOI010000154.1	hypothetical protein	COG0659	-	-
JAMCOI010000154.1	Probable low-affinity inorganic phosphate transporter	COG0306	K14640	-
JAMCOI010000154.1	Protein AIM2	COG0412	-	-
JAMCOI010000154.1	hypothetical protein	COG0624	K01293	-
JAMCOI010000154.1	Ribose-phosphate pyrophosphokinase (EC 2.7.6.1)	COG0462	K00948	-
JAMCOI010000154.1	hypothetical protein		K08330	-
JAMCOI010000154.1	hypothetical protein	COG0592	K04802	-
JAMCOI010000154.1	Acetyltransferase	COG0456	K00670	-
JAMCOI010000154.1	Fructose-bisphosphate aldolase class II (EC 4.1.2.13)	COG0191	K01192,K01624	-
JAMCOI010000154.1	hypothetical protein		K17793	-
JAMCOI010000154.1	Probable tubulin--tyrosine ligase PBY1		-	-
JAMCOI010000154.1	hypothetical protein	COG5648	-	-
JAMCOI010000155.1	hypothetical protein	COG5241	K10849	-
JAMCOI010000155.1	Prefoldin subunit 5	COG1730	K04797	-
JAMCOI010000155.1	hypothetical protein		K11763	-

JAMCOI010000155.1	hypothetical protein	COG2939	K13289	-
JAMCOI010000155.1	hypothetical protein	COG0055	K11446	-
JAMCOI010000155.1	LSU ribosomal protein L7e (L30p)	COG1841	K02937	-
JAMCOI010000155.1	hypothetical protein	COG0531	K09885	-
JAMCOI010000155.1	hypothetical protein		K08341	-
JAMCOI010000155.1	hypothetical protein	COG2234	K05994	-
JAMCOI010000155.1	hypothetical protein	COG2234	K05994	-
JAMCOI010000155.1	hypothetical protein	COG5308	K14312	-
JAMCOI010000155.1	Phosphoribulokinase / Uridine kinase family	COG0572	K00876	-
JAMCOI010000155.1	Eukaryotic translation initiation factor 4A	COG0513	K13025	-
JAMCOI010000155.1	hypothetical protein		K20221	-
JAMCOI010000155.1	hypothetical protein		K04627	-
JAMCOI010000155.1	Aspartyl-tRNA(Asn) amidotransferase subunit B (EC 6.3.5.6) @ Glutamyl-tRNA(Gln) amidotransferase subunit B (EC 6.3.5.7)	COG0064	K02434	-
JAMCOI010000155.1	hypothetical protein		K00706	GT48
JAMCOI010000155.1	hypothetical protein		K20062	-
JAMCOI010000155.1	alcohol acyl transferase	COG0429	K07019	-
JAMCOI010000155.1	Transcription accessory protein (S1 RNA-binding domain)	COG2135	-	-
JAMCOI010000155.1	hypothetical protein		K17413	-
JAMCOI010000155.1	hypothetical protein		K11315	-
JAMCOI010000155.1	Ubiquinol-cytochrome C reductase complex core protein I, mitochondrial precursor (EC 1.10.2.2)	COG0612	K17732	-
JAMCOI010000155.1	hypothetical protein Belongs to the actin family	COG5277	K11400,K11767	-
JAMCOI010000155.1	3-hydroxyisobutyryl-CoA hydrolase (EC 3.1.2.4)	COG1024	K05605	-
JAMCOI010000155.1	hypothetical protein		K17908	-
JAMCOI010000155.1	hypothetical protein	COG5232	K12275	-
JAMCOI010000155.1	RNase L inhibitor	COG1245	K06174	-
JAMCOI010000155.1	Chromo domain-containing protein 1	COG0553	K11367	-
JAMCOI010000155.1	hypothetical protein		K13126	-
JAMCOI010000155.1	Alpha,alpha-trehalose-phosphate synthase [UDP-forming] (EC 2.4.1.15)	COG1877	K16055	GT20
JAMCOI010000155.1	Replication-associated recombination protein RarA	COG2256	K07478	-
JAMCOI010000155.1	hypothetical protein		K14960	-
JAMCOI010000155.1	UPF0047 protein YjbQ	COG0432	-	-
JAMCOI010000155.1	hypothetical protein	COG0024	K14813	-
JAMCOI010000155.1	Triosephosphate isomerase (EC 5.3.1.1)	COG5040	K06630	-
JAMCOI010000155.1	hypothetical protein	COG5019	K16948	-
JAMCOI010000155.1	Octanoate-[acyl-carrier-protein]-protein-N-octanoyltransferase	COG0321	K03801	-
JAMCOI010000155.1	Ubiquinone biosynthesis protein COQ4, mitochondrial precursor	COG5031	K18586	-
JAMCOI010000155.1	hypothetical protein		K22240	-
JAMCOI010000155.1	hypothetical protein		K12404	-
JAMCOI010000155.1	hypothetical protein		K11572	-
JAMCOI010000155.1	hypothetical protein	COG1716	-	-
JAMCOI010000155.1	Succinate dehydrogenase hydrophobic membrane anchor protein		K00237	-
JAMCOI010000155.1	hypothetical protein	COG5273	K16675,K20030	-
JAMCOI010000155.1	tRNA-splicing endonuclease subunit SEN15 (EC 3.1.27.9)		K15325	-
JAMCOI010000155.1	hypothetical protein		K18638	-
JAMCOI010000155.1	Exosome complex RNA binding Csl4	COG1096	K07573	-
JAMCOI010000155.1	hypothetical protein		K15077	-
JAMCOI010000155.1	RNase P subunit p30	COG1603	K03539	-
JAMCOI010000155.1	hypothetical protein		K07059	-
JAMCOI010000155.1	Glycolipid 2-alpha-mannosyltransferase	COG5020	K03854	GT15
JAMCOI010000155.1	Aspartyl-tRNA synthetase (EC 6.1.1.12)	COG0173	K01876	-
JAMCOI010000155.1	hypothetical protein		K14790	-
JAMCOI010000156.1	proteasome subunit beta1 (EC 3.4.25.1)		K02738	-
JAMCOI010000156.1	Peptidyl-prolyl cis-trans isomerase (EC 5.2.1.8)	COG0652	K01802,K03768,K	-
JAMCOI010000156.1	hypothetical protein		K12833	-
JAMCOI010000156.1	hypothetical protein		K12666	-
JAMCOI010000156.1	hypothetical protein	COG0699	K17065	-
JAMCOI010000156.1	hypothetical protein	COG5210	K20176	-
JAMCOI010000156.1	Glycolipid 2-alpha-mannosyltransferase	COG5020	K10967	GT15
JAMCOI010000156.1	beta-glucosidase (EC 3.2.1.21)	COG1472	K01188,K05349	GH1,GH3
JAMCOI010000156.1	Glycolipid 2-alpha-mannosyltransferase	COG5020	K10967	GT15
JAMCOI010000156.1	hypothetical protein		K11242	-
JAMCOI010000156.1	Cell division-associated, ATP-dependent zinc metalloprotease FtsH	COG0465	K08956	-
JAMCOI010000156.1	hypothetical protein		K18177	-
JAMCOI010000156.1	SNF2 family N-terminal domain	COG0553	-	-
JAMCOI010000156.1	SNF2 family N-terminal domain	COG0553	-	-
JAMCOI010000156.1	hypothetical protein	COG2273	-	-

JAMCOI010000156.1	High-affinity carbon uptake protein Hat/HatR		K05236	-
JAMCOI010000156.1	Serine/threonine protein phosphatase (EC 3.1.3.16)	COG0639	K04460	-
JAMCOI010000156.1	hypothetical protein	COG5647	K03347	-
JAMCOI010000156.1	V-type ATP synthase subunit A (EC 3.6.3.14)	COG1155	K02145	-
JAMCOI010000156.1	OHCU decarboxylase		-	-
JAMCOI010000156.1	hypothetical protein	COG0702	K03953	-
JAMCOI010000156.1	Serine/threonine protein phosphatase (EC 3.1.3.16)	COG0639	K04382	-
JAMCOI010000157.1	tRNA-5-carboxymethylaminomethyl-2-thiouridine(34) synthesis protein MnmG	COG0445	K03495,K12479	-
JAMCOI010000157.1	hypothetical protein		K14784	-
JAMCOI010000157.1	Vps51/Vps67		-	-
JAMCOI010000157.1	hypothetical protein	COG5224	K08064	-
JAMCOI010000157.1	hypothetical protein	COG5657	K18423	-
JAMCOI010000157.1	GDP-mannose transporter	COG5070	K15356	-
JAMCOI010000157.1	Amidophosphoribosyltransferase (EC 2.4.2.14)	COG0034	K00764	-
JAMCOI010000157.1	hypothetical protein	COG0513	K12614	-
JAMCOI010000157.1	hypothetical protein	COG0847	K14570	-
JAMCOI010000157.1	Transcription initiation factor IID 145 kDa subunit	COG5179	K03125	-
JAMCOI010000157.1	Histidine permease	COG0531	K16261	-
JAMCOI010000157.1	hypothetical protein	COG0480	K14536	-
JAMCOI010000157.1	Uncharacterized MFS-type transporter		-	-
JAMCOI010000158.1	Rhodanese Homology Domain	COG5105	K02555	-
JAMCOI010000158.1	Oxidoreductase, short-chain dehydrogenase/reductase family	COG2030	K14729	-
JAMCOI010000158.1	Uncharacterized MFS-type transporter		-	-
JAMCOI010000158.1	Rab-GTPase-TBC domain	COG5210	K20167	-
JAMCOI010000158.1	hypothetical protein	COG5171	K18722	-
JAMCOI010000158.1	SSU ribosomal protein S25e	COG4901	K02975	-
JAMCOI010000158.1	Polyketide cyclase / dehydrase and lipid transport	COG2867	K18588	-
JAMCOI010000158.1	hypothetical protein	COG0531	K16261	-
JAMCOI010000158.1	proteasome subunit beta7 (EC 3.4.25.1)		K02736	-
JAMCOI010000158.1	hypothetical protein	COG5037	K21631	-
JAMCOI010000158.1	hypothetical protein		K17807	-
JAMCOI010000158.1	Threonine synthase (EC 4.2.3.1)	COG0498	K01733	-
JAMCOI010000158.1	hypothetical protein		K19323	-
JAMCOI010000158.1	3',5'-cyclic-nucleotide phosphodiesterase (EC 3.1.4.17)	COG5212	K01120	-
JAMCOI010000158.1	hypothetical protein		K20471	-
JAMCOI010000158.1	Steryl acetyl hydrolase	COG0657	-	-
JAMCOI010000158.1	hypothetical protein	COG5291	K13989	-
JAMCOI010000158.1	hypothetical protein	COG5237	-	-
JAMCOI010000159.1	Cyclin		-	-
JAMCOI010000159.1	hypothetical protein		K18188	-
JAMCOI010000159.1	Catalase KatE (EC 1.11.1.6)	COG0753	K03781	-
JAMCOI010000159.1	hypothetical protein		K14845	-
JAMCOI010000159.1	LSU ribosomal protein L11p (L12e)	COG0080	K02867	-
JAMCOI010000159.1	hypothetical protein	COG1028	-	-
JAMCOI010000159.1	hypothetical protein	COG2319	K14829	-
JAMCOI010000159.1	hypothetical protein	COG0724	K14789	-
JAMCOI010000159.1	small GTPase superfamily. Rho family	COG1100	K04392	-
JAMCOI010000159.1	Aminobutyraldehyde dehydrogenase (EC 1.2.1.19)	COG0579	K20298	-
JAMCOI010000159.1	Transcription initiation factor IIH cyclin-dependent kinase 7		K02202	-
JAMCOI010000159.1	Sodium/bile acid symporter family	COG0385	K02731,K14347	-
JAMCOI010000159.1	hypothetical protein	COG5279	-	-
JAMCOI010000159.1	hypothetical protein	COG5032	K00914	-
JAMCOI010000159.1	hypothetical protein	COG0406	-	-
JAMCOI010000159.1	N-terminal acetyltransferase B complex catalytic subunit NAT3 (EC 2.3.1.88)	COG0456	K17972	-
JAMCOI010000159.1	hypothetical protein		K17773	-
JAMCOI010000159.1	hypothetical protein		K11274	-
JAMCOI010000160.1	hypothetical protein	COG0477	-	-
JAMCOI010000160.1	SSU ribosomal protein S5e (S7p)	COG0049	K02989	-
JAMCOI010000160.1	monocarboxylate transporter		-	-
JAMCOI010000160.1	monocarboxylate transporter		-	-
JAMCOI010000160.1	Vacuolar protein sorting-associated protein 4		K12196	-
JAMCOI010000160.1	hypothetical protein		K11098	-
JAMCOI010000160.1	hypothetical protein		K05293	-
JAMCOI010000160.1	hypothetical protein (COPII coat)		K20353	-
JAMCOI010000160.1	Protein tyrosine kinase		K08286,K14864	-
JAMCOI010000160.1	hypothetical protein		K15156	-
JAMCOI010000160.1	Lysine-specific permease	COG0531	K16261	-

JAMCOI010000160.1	hypothetical protein		K12627	-
JAMCOI010000160.1	Methylthioribulose-1-phosphate dehydratase (EC 4.2.1.109)	COG0235	K08964	-
JAMCOI010000161.1	hypothetical protein	COG0678	K14171	-
JAMCOI010000161.1	hypothetical protein		K05287	-
JAMCOI010000161.1	hypothetical protein	COG5161	K14401	-
JAMCOI010000161.1	Thioredoxin	COG0526	-	-
JAMCOI010000161.1	hypothetical protein	COG5196	K10949	-
JAMCOI010000161.1	proteasome subunit beta6 (EC 3.4.25.1)	COG0638	K02732	-
JAMCOI010000161.1	hypothetical protein		K17781	-
JAMCOI010000161.1	hypothetical protein	COG0678	K14171	-
JAMCOI010000161.1	hypothetical protein		K09466	-
JAMCOI010000161.1	hypothetical protein		K09246	-
JAMCOI010000161.1	hypothetical protein		K09243,K21547	-
JAMCOI010000161.1	hypothetical protein		K17086	-
JAMCOI010000161.1	hypothetical protein		K21641	-
JAMCOI010000162.1	helicase superfamily c-terminal domain	COG1061	K17677	-
JAMCOI010000162.1	hypothetical protein	COG5214	K02321	-
JAMCOI010000162.1	hypothetical protein		K11090	-
JAMCOI010000162.1	Pyridoxal phosphate-containing protein YggS	COG0325	K06997	-
JAMCOI010000162.1	hypothetical protein		K19327	-
JAMCOI010000162.1	hypothetical protein		K14962	-
JAMCOI010000162.1	hypothetical protein		K20521	-
JAMCOI010000162.1	hypothetical protein		K14851	-
JAMCOI010000162.1	hypothetical protein		K12592	-
JAMCOI010000162.1	Endoribonuclease (EC 3.1.26.-) IRE1 / Serine,threonine protein kinase (EC 2.7.11.1)	COG0515	K08852	-
JAMCOI010000162.1	hypothetical protein		K10991	-
JAMCOI010000163.1	hypothetical protein		K08794	-
JAMCOI010000163.1	hypothetical protein	COG5141	K11380	-
JAMCOI010000163.1	hypothetical protein		K09448	-
JAMCOI010000163.1	N5-carboxyaminoimidazole ribonucleotide mutase (EC 5.4.99.18)	COG0152	K11808	-
JAMCOI010000163.1	LSU ribosomal protein L19e	COG2147	K02885	-
JAMCOI010000163.1	hypothetical protein	COG1814	-	-
JAMCOI010000163.1	hypothetical protein		K19613	-
JAMCOI010000163.1	hypothetical protein		K19613	-
JAMCOI010000163.1	hypothetical protein		K19613	-
JAMCOI010000163.1	hypothetical protein		K19613	-
JAMCOI010000163.1	hypothetical protein		K19613	-
JAMCOI010000164.1	Heat Shock Protein	COG5524	-	-
JAMCOI010000164.1	ABC-2 type transporter	COG1131	-	-
JAMCOI010000164.1	hypothetical protein	COG4286	-	-
JAMCOI010000164.1	hypothetical protein	COG2016	K15027	-
JAMCOI010000164.1	Cystathionine beta-synthase (EC 4.2.1.22)	COG0031	K01697	-
JAMCOI010000164.1	Transcription elongation factor S-II	COG1594	K03145	-
JAMCOI010000164.1	hypothetical protein	COG0724	K14407	-
JAMCOI010000164.1	hypothetical protein		K10576	-
JAMCOI010000164.1	hypothetical protein	COG5333	K06634	-
JAMCOI010000164.1	Succinate-semialdehyde dehydrogenase [NAD(P)+] (EC 1.2.1.16)	COG1012	K00135	-
JAMCOI010000164.1	hypothetical protein	COG0666	K06649	-
JAMCOI010000164.1	Sugar (and other) transporter		-	-
JAMCOI010000164.1	hypothetical protein	COG0524	-	-

Table S5: Comparitive analysis of insilico metabolic characersization and summary of putative metabolic genes identified in the Metagenome-Assembled Genomes of two endo-archaea Bin001 and Bin002 of *Candida tropicalis* JY101

S.No	Pathway Name	Enzyme/Reaction	Bin001	Bin002
1	1,4-Dichlorobenzene degradation	Hypothetical NagD-like phosphatase; 4-nitrophenylphosphatase (EC 3.1.3.41), YDL236W homolog	No	Yes
2	Alanine, aspartate and glutamate metabolism	Aspartate aminotransferase (EC 2.6.1.1)	Yes	No
		Carbamoyl-phosphate synthase large chain (EC 6.3.5.5)	Yes	No
		Carbamoyl-phosphate synthase small chain (EC 6.3.5.5)	Yes	No
		Glutamine synthetase type II, (EC 6.3.1.2)	Yes	No
		L-asparaginase (EC 3.5.1.1)	No	Yes
3	alpha-Linolenic acid metabolism	3-ketoacyl-CoA thiolase (EC 2.3.1.16) @ Acetyl-CoA acetyltransferase (EC 2.3.1.9)	Yes	No
4	Amino sugar and nucleotide sugar metabolism	Chitin synthase (EC 2.4.1.16)	Yes	Yes
		Glucosamine-6-phosphate deaminase (EC 3.5.99.6)	Yes	No
		Glutamine--fructose-6-phosphate aminotransferase [isomerizing] (EC 2.6.1.16)	Yes	No
		Mannose-1-phosphate guanylyltransferase (EC 2.7.7.13)	Yes	Yes
		N-acetylglucosamine-1-phosphate uridyltransferase (EC 2.7.7.23)	No	Yes
		N-acetylglucosamine-6-phosphate deacetylase (EC 3.5.1.25)	Yes	No
		Arginyl-tRNA synthetase (EC 6.1.1.19)		Yes
		Asparaginyl-tRNA synthetase (EC 6.1.1.22)	Yes	Yes
		Aspartyl-tRNA synthetase (EC 6.1.1.12)	Yes	Yes
		Aspartyl-tRNA(Asn) amidotransferase subunit A (EC 6.3.5.6) @ Glutamyl-tRNA(Gln) amidotransferase subunit A (EC 6.3.5.7)	Yes	Yes
		Aspartyl-tRNA(Asn) amidotransferase subunit B (EC 6.3.5.6) @ Glutamyl-tRNA(Gln) amidotransferase subunit B (EC 6.3.5.7)	Yes	Yes
		Cysteinyl-tRNA synthetase (EC 6.1.1.16)	No	Yes
		Glutaminyl-tRNA synthetase (EC 6.1.1.18)	No	Yes
		Glutamyl-tRNA synthetase (EC 6.1.1.17)	Yes	Yes

5	Aminoacyl-tRNA biosynthesis	Glycyl-tRNA synthetase (EC 6.1.1.14) @ Glycyl-tRNA synthetase (EC 6.1.1.14), mitochondrial	Yes	No
		Histidyl-tRNA synthetase (EC 6.1.1.21) @ Histidyl-tRNA synthetase (EC 6.1.1.21), mitochondrial	Yes	No
		Isoleucyl-tRNA synthetase (EC 6.1.1.5)	Yes	Yes
		Leucyl-tRNA synthetase (EC 6.1.1.4)	No	Yes
		Lysyl-tRNA synthetase (class II) (EC 6.1.1.6)	Yes	Yes
		Phenylalanyl-tRNA synthetase (EC 6.1.1.20)	Yes	Yes
		Prolyl-tRNA synthetase (EC 6.1.1.15), archaeal/eukaryal type	Yes	No
		Seryl-tRNA synthetase (EC 6.1.1.11)	Yes	Yes
		Threonyl-tRNA synthetase (EC 6.1.1.3)	Yes	No
		Tryptophanyl-tRNA synthetase (EC 6.1.1.2)	Yes	Yes
		Tyrosyl-tRNA synthetase (EC 6.1.1.1)	Yes	No
6	Arachidonic acid metabolism	Glutathione peroxidase (EC 1.11.1.9) @ Thioredoxin peroxidase (EC 1.11.1.15)	Yes	No
7	Arginine and proline metabolism	Aminobutyraldehyde dehydrogenase (EC 1.2.1.19)	No	Yes
		Arginase (EC 3.5.3.1)	No	Yes
		Argininosuccinate synthase (EC 6.3.4.5)	Yes	No
		Cytosine deaminase (EC 3.5.4.1)	Yes	No
		Gamma-glutamyl phosphate reductase (EC 1.2.1.41)	Yes	No
		Glutamate 5-kinase (EC 2.7.2.11) / RNA-binding C-terminal domain PUA	No	Yes
		Glutamate N-acetyltransferase (EC 2.3.1.35) @ N-acetylglutamate synthase (EC 2.3.1.1)	No	Yes
		Guanidinobutyrase (EC 3.5.3.7)	Yes	No
		N-acetylglutamate kinase (EC 2.7.2.8) / N-acetyl-gamma-glutamyl-phosphate reductase (EC 1.2.1.38)	No	Yes
		N-acetylornithine aminotransferase (EC 2.6.1.11)	No	Yes
		NADP-specific glutamate dehydrogenase (EC 1.4.1.4)	Yes	No
		Ornithine aminotransferase (EC 2.6.1.13)	Yes	No
		Spermidine synthase (EC 2.5.1.16)	Yes	No
8	Atrazine degradation	Allophanate hydrolase (EC 3.5.1.54)	Yes	No
9	Ascorbate and aldarate metabolism	Aldehyde dehydrogenase (EC 1.2.1.3)	No	Yes
		Inositol oxygenase (EC 1.13.99.1)	No	Yes

10	beta-Alanine metabolism	Pantoate--beta-alanine ligase (EC 6.3.2.1)	No	Yes
11	Benzoate degradation via hydroxylation	Catechol 1,2-dioxygenase 1 (EC 1.13.11.1)	Yes	No
12	Biosynthesis of type II polyketide products	Protein arginine N-methyltransferase 1 (EC 2.1.1.-)	No	Yes
13	Biotin metabolism	Acetyl transferase of FASI (EC 2.3.1.38)	Yes	No
		Dethiobiotin synthase BioD (EC 6.3.3.3)	Yes	No
		Adenosylmethionine-8-amino-7-oxononanoate aminotransferase (EC 2.6.1.62)	Yes	No
		Biotin--protein ligase (EC 6.3.4.9)(EC 6.3.4.10)(EC 6.3.4.11)(EC 6.3.4.15)	No	Yes
14	Butanoate metabolism	Succinyl-CoA:3-ketoacid-coenzyme A transferase subunit B (EC 2.8.3.5)	Yes	No
		Glutamate decarboxylase (EC 4.1.1.15)	Yes	No
		Pyruvate dehydrogenase E1 component beta subunit (EC 1.2.4.1)	No	Yes
		4-aminobutyrate aminotransferase (EC 2.6.1.19)	No	Yes
		Succinate-semialdehyde dehydrogenase [NAD(P)+] (EC 1.2.1.16)	No	Yes
15	C21-Steroid hormone metabolism	3-oxoacyl-coenzyme A reductase of elongase (EC 1.1.1.62)	No	Yes
16	Carbon fixation in photosynthetic organisms	Phosphoglycerate kinase (EC 2.7.2.3)	Yes	No
		Transketolase (EC 2.2.1.1)	No	Yes
17	Citrate cycle (TCA cycle)	Malate dehydrogenase (EC 1.1.1.37)	Yes	No
		Isocitrate dehydrogenase [NAD] subunit I, mitochondrial precursor (EC 1.1.1.41)	Yes	No
		Citrate synthase (si) (EC 2.3.3.1)	Yes	No
		Isocitrate dehydrogenase [NAD] subunit II, mitochondrial precursor (EC 1.1.1.41)	Yes	No
		Dihydrolipoamide succinyltransferase component (E2) of 2-oxoglutarate dehydrogenase complex (EC 2.3.1.61)	Yes	Yes
18	Cyanoamino acid metabolism	beta-glucosidase (EC 3.2.1.21)	No	Yes
		Gamma-glutamyltranspeptidase (EC 2.3.2.2) @ Glutathione hydrolase (EC 3.4.19.13)	Yes	No
		5'-methylthioadenosine phosphorylase (EC 2.4.2.28)	No	Yes
		Adenosylhomocysteinase (EC 3.3.1.1)	No	Yes
		Cystathionine beta-synthase (EC 4.2.1.22)	No	Yes

19	Cysteine and methionine metabolism	Cystathionine gamma-synthase (EC 2.5.1.48)	Yes	No
		Homoserine O-acetyltransferase (EC 2.3.1.31)	Yes	No
		L-serine dehydratase, (PLP)-dependent (EC 4.3.1.17) @ Threonine dehydratase (EC 4.3.1.19), type	Yes	No
		Methylthioribulose-1-phosphate dehydratase (EC 4.2.1.109)	No	Yes
		O-acetylhomoserine sulfhydrylase (EC 2.5.1.49) @ O-succinylhomoserine sulfhydrylase (EC 2.5.1.48)	Yes	No
		S-adenosylmethionine decarboxylase proenzyme (EC 4.1.1.50), eukaryotic	No	Yes
		S-adenosylmethionine synthetase (EC 2.5.1.6)	Yes	No
20	Drug metabolism	Glutathione S-transferase, omega (EC 2.5.1.18)	Yes	No
		Uridine kinase, type 2 (EC 2.7.1.48) / Uracil phosphoribosyltransferase (EC 2.4.2.9)	Yes	No
21	Fatty acid biosynthesis	Malonyl CoA-acyl carrier protein transacylase (EC 2.3.1.39)	Yes	No
		Acyl carrier protein of FASI / 3-oxoacyl-[acyl-carrier-protein] reductase of FASI (EC 1.1.1.100) / 3-oxoacyl-[acyl-carrier-protein] synthase of FASI (EC 2.3.1.41)	Yes	No
		3-oxoacyl-[acyl-carrier-protein] synthase, KASII (EC 2.3.1.179)	No	Yes
22	Fatty acid metabolism	Aldehyde dehydrogenase (EC 1.2.1.3)	Yes	Yes
		Long-chain-fatty-acid--CoA ligase (EC 6.2.1.3)	Yes	Yes
23	Folate biosynthesis	GTP cyclohydrolase I (EC 3.5.4.16) type 1	Yes	No
		Alkaline phosphatase (EC 3.1.3.1)	Yes	No
		Dihydrofolate reductase (EC 1.5.1.3)	Yes	No
24	Fructose and mannose metabolism	Fructose-1,6-bisphosphatase, type I (EC 3.1.3.11)	Yes	No
		Phosphomannomutase (EC 5.4.2.8)	Yes	No
		6-phosphofructo-2-kinase (EC 2.7.1.105)	Yes	No
		6-phosphofructokinase, fungal/animal type (EC 2.7.1.11)	No	Yes
		Fructose-bisphosphate aldolase class II (EC 4.1.2.13)	No	Yes
25	Galactose metabolism	Oligo-1,6-glucosidase (EC 3.2.1.10)	Yes	Yes
		Hexokinase (EC 2.7.1.1)	Yes	Yes
		5-oxoprolinase (EC 3.5.2.9), HyuA-like domain / 5-oxoprolinase (EC 3.5.2.9), HyuB-like domain	Yes	No

26	Glutathione metabolism	Glutathione peroxidase (EC 1.11.1.9) @ Thioredoxin peroxidase (EC 1.11.1.15)	Yes	No
		Glutathione S-transferase (EC 2.5.1.18)	Yes	No
		Glutathione synthetase (EC 6.3.2.3)	Yes	No
		Pyridoxal 5-phosphate (PLP)-dependent ornithine decarboxylase (EC 4.1.1.17)	No	Yes
		Ribonucleotide reductase of class Ia (aerobic), beta subunit (EC 1.17.4.1)	Yes	No
27	Glycerolipid metabolism	Acyl-CoA:1-acyl-sn-glycerol-3-phosphate acyltransferase (EC 2.3.1.51) @ Acyl-ACP:1-acyl-sn-glycerol-3-phosphate acyltransferase (EC 2.3.1.n4)	Yes	No
		Glycerol-3-phosphate O-acyltransferase (EC 2.3.1.15) @ Glycerone-phosphate O-acyltransferase (EC 2.3.1.42)	Yes	No
		Phospholipid:diacylglycerol acyltransferase (EC 2.3.1.158)	No	Yes
		Dihydroxyacetone kinase, ATP-dependent (EC 2.7.1.29)	No	Yes
28	Glycerophospholipid metabolism	Choline kinase (EC 2.7.1.32)	Yes	No
		Glycerol-3-phosphate dehydrogenase (EC 1.1.5.3)	Yes	No
		Glycerol-3-phosphate O-acyltransferase (EC 2.3.1.15) @ Glycerone-phosphate O-acyltransferase (EC 2.3.1.42)	No	Yes
		Phosphatidate cytidylyltransferase (EC 2.7.7.41)		Yes
		Phosphatidate phosphatase (EC 3.1.3.4)	Yes	
		Phosphatidyl-N-methylethanolamine N-methyltransferase (EC 2.1.1.17)(EC 2.1.1.71)		Yes
		Phosphatidylserine decarboxylase (EC 4.1.1.65)	Yes	No
		Phosphocholine cytidylyltransferase (EC 2.7.7.15)	Yes	No
29	Glycine, serine and threonine metabolism	Phosphoserine phosphatase (EC 3.1.3.3)	Yes	No
		L-serine dehydratase, (PLP)-dependent (EC 4.3.1.17) @ Threonine dehydratase (EC 4.3.1.19), type	Yes	No
		Alcohol dehydrogenase (EC 1.1.1.1)	Yes	No
		5-aminolevulinate synthase (EC 2.3.1.37)	Yes	No
		Homoserine kinase (EC 2.7.1.39)	No	Yes
		Threonine synthase (EC 4.2.3.1)	No	Yes

		Glycine dehydrogenase [decarboxylating] (glycine cleavage system P protein) (EC 1.4.4.2)	No	Yes
		CDP-diacylglycerol--serine O-phosphatidyltransferase (EC 2.7.8.8)	No	Yes
30	Glyoxylate and dicarboxylate metabolism	Malate dehydrogenase (EC 1.1.1.37)	No	Yes
		NAD-dependent formate dehydrogenase (EC 1.2.1.2)	Yes	No
31	Histidine metabolism	ATP phosphoribosyltransferase (EC 2.4.2.17) => HisGl	Yes	No
		Imidazoleglycerol-phosphate dehydratase (EC 4.2.1.19)	Yes	No
32	Inositol phosphate metabolism	CDP-diacylglycerol--inositol 3-phosphatidyltransferase (EC 2.7.8.11)	Yes	No
		Triosephosphate isomerase (EC 5.3.1.1)	Yes	Yes
		Inositol-1-phosphate synthase (EC 5.5.1.4)	No	Yes
33	Isoquinoline alkaloid biosynthesis	Aspartate aminotransferase (EC 2.6.1.1)	No	Yes
34	Lipoic acid metabolism	Lipoyl synthase (EC 2.8.1.8)	No	Yes
35	Lysine biosynthesis	Aspartokinase (EC 2.7.2.4)	No	Yes
		Homoisocitrate dehydrogenase (EC 1.1.1.87)	Yes	No
36	Lysine degradation	2-oxoglutarate dehydrogenase E1 component (EC 1.2.4.2)	Yes	No
		L-2-aminoadipate reductase (EC 1.2.1.31) (EC 1.2.1.95)	Yes	No
		Saccharopine dehydrogenase [NAD+, L-lysine-forming] (EC 1.5.1.7)	No	Yes
		Saccharopine dehydrogenase [NADP, L-glutamate-forming] (EC 1.5.1.10)	No	Yes
37	Methane metabolism	Serine hydroxymethyltransferase (EC 2.1.2.1)	Yes	No
		S-formylglutathione hydrolase (EC 3.1.2.12)	No	Yes
		Malate dehydrogenase (EC 1.1.1.37)	No	Yes
		D-3-phosphoglycerate dehydrogenase (EC 1.1.1.95)	No	Yes
		Catalase KatE (EC 1.11.1.6)	No	Yes
		S-(hydroxymethyl)glutathione dehydrogenase (EC 1.1.1.284)	No	Yes
38	mTOR signaling pathway	Serine/threonine-protein kinase RIO2 (EC 2.7.11.1)	Yes	Yes
		Serine/threonine-protein kinase RIO1 (EC 2.7.11.1)	Yes	Yes
		Dolichyl-phosphate beta-glucosyltransferase (EC 2.4.1.117)	No	Yes
		Dolichol kinase (EC 2.7.1.108)	No	Yes
		Glucan 1,3-alpha-glucosidase, subunit alpha (EC 3.2.1.84)	Yes	No

39	N-Glycan biosynthesis	Mannosyl-oligosaccharide glucosidase (EC 3.2.1.106)	Yes	No
		UDP-N-acetylglucosamine--dolichyl-phosphate N-acetylglucosaminephosphotransferase (EC 2.7.8.15)	Yes	No
40	Nicotinate and nicotinamide metabolism	NAD kinase (EC 2.7.1.23)	Yes	No
		Nicotinate-nucleotide adenylyltransferase (EC 2.7.7.18), PNAT family @ Nicotinamide-nucleotide adenylyltransferase (EC 2.7.7.1), PNAT family	Yes	No
		NAD synthetase (EC 6.3.1.5) / Glutamine amidotransferase chain of NAD synthetase	Yes	No
		Nicotinamidase (EC 3.5.1.19)	No	Yes
41	Nitrogen metabolism	NAD-specific glutamate dehydrogenase (EC 1.4.1.2), type	Yes	No
		Formamidase (EC 3.5.1.49)	Yes	No
		NADH-ubiquinone oxidoreductase chain I (EC 1.6.5.3)	Yes	No
		Ubiquinol-cytochrome C reductase iron-sulfur subunit (EC 1.10.2.2)	Yes	No
		Carbonic anhydrase, beta class (EC 4.2.1.1)	No	Yes
		NADH-ubiquinone oxidoreductase chain D (EC 1.6.5.3)	No	Yes
		NADH-ubiquinone oxidoreductase chain G (EC 1.6.5.3)	No	Yes
42	Novobiocin biosynthesis	Prephenate and/or arogenate dehydrogenase (unknown specificity) (EC 1.3.1.12)(EC 1.3.1.43)	No	Yes
43	One carbon pool by folate	5,10-methylenetetrahydrofolate reductase (EC 1.5.1.20)	Yes	Yes
		Phosphoribosylglycinamide formyltransferase (EC 2.1.2.2)	Yes	No
		Thymidylate synthase (EC 2.1.1.45)	Yes	No
44	Oxidative phosphorylation	ATP synthase alpha chain (EC 3.6.3.14)	Yes	No
		ATP synthase gamma chain (EC 3.6.3.14)	Yes	No
		Cytochrome c oxidase polypeptide Va, mitochondrial precursor (EC 1.9.3.1)	Yes	Yes
		Inorganic pyrophosphatase (EC 3.6.1.1)	Yes	No
		NADH dehydrogenase (EC 1.6.99.3)	Yes	No
		NADH-ubiquinone oxidoreductase chain B (EC 1.6.5.3)	Yes	No
		NADH-ubiquinone oxidoreductase chain E (EC 1.6.5.3)	Yes	No
		Succinate dehydrogenase flavoprotein subunit (EC 1.3.5.1)	Yes	No

		Ubiquinol-cytochrome C reductase complex core protein I, mitochondrial precursor (EC 1.10.2.2)	Yes	Yes
		V-type ATP synthase subunit K (EC 3.6.3.14)	Yes	No
45	Pantothenate and CoA biosynthesis	Dephospho-CoA kinase (EC 2.7.1.24)	Yes	No
		Phosphopantothenoylcysteine decarboxylase (EC 4.1.1.36)	Yes	No
		3-methyl-2-oxobutanoate hydroxymethyltransferase (EC 2.1.2.11)	Yes	No
		Phosphopantothenoylcysteine synthetase (EC 6.3.2.5)	Yes	No
		Pantothenate kinase type II (EC 2.7.1.33)	No	Yes
46	Pentose and glucuronate interconversions	D-ribulokinase (EC 2.7.1.47)	Yes	No
		Xylulose kinase (EC 2.7.1.17)	No	Yes
47	Pentose phosphate pathway	Ribulose-phosphate 3-epimerase (EC 5.1.3.1)	Yes	No
		6-phosphogluconolactonase (EC 3.1.1.31)	No	Yes
48	Phenylalanine, tyrosine and tryptophan biosynthesis	2-keto-3-deoxy-D-arabino-heptulosonate-7-phosphate synthase I alpha (EC 2.5.1.54)	No	Yes
		3-dehydroquinate dehydratase II (EC 4.2.1.10)	Yes	No
		3-dehydroquinate synthase (EC 4.2.3.4)	Yes	No
		Anthranilate phosphoribosyltransferase (EC 2.4.2.18)	Yes	No
		Chorismate mutase III (EC 5.4.99.5)	No	Yes
		Histidinol-phosphate aminotransferase (EC 2.6.1.9)	Yes	
		Indole-3-glycerol phosphate synthase (EC 4.1.1.48)	Yes	No
		Phosphoribosylanthranilate isomerase (EC 5.3.1.24)	Yes	No
		Tryptophan synthase beta chain (EC 4.2.1.20)	Yes	No
49	Phenylpropanoid biosynthesis	Pyruvate decarboxylase (EC 4.1.1.1); Alpha-keto-acid decarboxylase (EC 4.1.1.-)	No	Yes
50	Photosynthesis	ATP synthase alpha chain (EC 3.6.3.14)	Yes	No
		Ferredoxin--NADP(+) reductase (EC 1.18.1.2)	Yes	No
		ATP synthase delta chain (EC 3.6.3.14)	Yes	No
		V-type ATP synthase subunit D (EC 3.6.3.14)	No	Yes
		V-type ATP synthase subunit A (EC 3.6.3.14)	No	Yes

		V-type ATP synthase subunit K (EC 3.6.3.14)	No	Yes
51	Porphyrin and chlorophyll metabolism	Ferrochelatase, protoheme ferro-lyase (EC 4.99.1.1)	Yes	No
		Cytochrome C1 heme lyase CCHL (EC 4.4.1.17)	Yes	No
		Porphobilinogen deaminase (EC 2.5.1.61)	Yes	No
		Coproporphyrinogen III oxidase, aerobic (EC 1.3.3.3)	Yes	No
		Porphobilinogen synthase (EC 4.2.1.24)	Yes	No
		Precorrin-2 oxidase (EC 1.3.1.76) @ Sirohydrochlorin ferrochelatase activity of CysG (EC 4.99.1.4) / Uroporphyrinogen-III methyltransferase (EC 2.1.1.107)	No	Yes
52	Propanoate metabolism	Succinyl-CoA ligase [ADP-forming] alpha chain (EC 6.2.1.5)	No	Yes
53	Purine metabolism	3',5'-cyclic-nucleotide phosphodiesterase (EC 3.1.4.17)	No	Yes
		Adenine phosphoribosyltransferase (EC 2.4.2.7)	Yes	No
		Adenosine deaminase (EC 3.5.4.4)	Yes	No
		Adenylate kinase (EC 2.7.4.3)	Yes	No
		Adenylosuccinate lyase (EC 4.3.2.2) @ SAICAR lyase (EC 4.3.2.2)	No	Yes
		Adenylosuccinate synthetase (EC 6.3.4.4)	Yes	No
		ADP-ribose pyrophosphatase (EC 3.6.1.13)	Yes	No
		Allantoicase (EC 3.5.3.4)	Yes	No
		Amidophosphoribosyltransferase (EC 2.4.2.14)	No	Yes
		DNA polymerase alpha catalytic subunit A (EC 2.7.7.7)	Yes	No
		DNA-directed RNA polymerase I second largest subunit (EC 2.7.7.6)	Yes	Yes
		DNA-directed RNA polymerase II 13.2 kDa polypeptide (EC 2.7.7.6)	Yes	No
		GMP synthase [glutamine-hydrolyzing], amidotransferase subunit (EC 6.3.5.2) / GMP synthase [glutamine-hydrolyzing], ATP pyrophosphatase subunit (EC 6.3.5.2)	Yes	No
		IMP cyclohydrolase (EC 3.5.4.10) / Phosphoribosylaminoimidazolecarboxamide formyltransferase (EC 2.1.2.3)	Yes	No
		N5-carboxyaminoimidazole ribonucleotide mutase (EC 5.4.99.18)	No	Yes

		Phosphoribosylformylglycinamidine synthase, synthetase subunit (EC 6.3.5.3) / Phosphoribosylformylglycinamidine synthase, glutamine amidotransferase subunit (EC 6.3.5.3)	Yes	No
		Ribonucleotide reductase of class Ia (aerobic), alpha subunit (EC 1.17.4.1)	Yes	No
		Ribose-phosphate pyrophosphokinase (EC 2.7.6.1)	Yes	No
		Ribose-phosphate pyrophosphokinase (EC 2.7.6.1)	Yes	Yes
		Uricase (urate oxidase) (EC 1.7.3.3)	Yes	No
		Xanthine-guanine phosphoribosyltransferase (EC 2.4.2.22)	Yes	No
54	Pyrimidine metabolism	CTP synthase (EC 6.3.4.2)	No	Yes
		Cytidine deaminase (EC 3.5.4.5)	Yes	No
		dCMP deaminase (EC 3.5.4.12)	Yes	No
		Deoxyuridine 5'-triphosphate nucleotidohydrolase (EC 3.6.1.23)	No	Yes
		Dihydroorotase (EC 3.5.2.3)	Yes	No
		Dihydroorotate dehydrogenase (quinone) (EC 1.3.5.2)	Yes	No
		DNA polymerase delta catalytic subunit (EC 2.7.7.7)	Yes	No
		DNA-directed RNA polymerase I 13.7 kDa polypeptide (EC 2.7.7.6)	Yes	No
		DNA-directed RNA polymerase I 49 kDa polypeptide (EC 2.7.7.6)	Yes	No
		DNA-directed RNA polymerase II 13.3 kDa polypeptide (EC 2.7.7.6)	Yes	Yes
		DNA-directed RNA polymerase II 19 kDa polypeptide (EC 2.7.7.6)	Yes	Yes
		DNA-directed RNA polymerase II second largest subunit (EC 2.7.7.6)	Yes	Yes
		DNA-directed RNA polymerase III 12.5 kDa polypeptide (EC 2.7.7.6)	Yes	No
		DNA-directed RNA polymerase III 31 kDa polypeptide (EC 2.7.7.6)	Yes	Yes
		DNA-directed RNA polymerase III largest subunit (EC 2.7.7.6)	Yes	No
		DNA-directed RNA polymerase III second largest subunit (EC 2.7.7.6)	Yes	No
		DNA-directed RNA polymerases I and III 40 kDa polypeptide (EC 2.7.7.6)	Yes	Yes
		DNA-directed RNA polymerases I, II, and III 8.3 kDa polypeptide (EC 2.7.7.6)	Yes	No
		Pseudouridine 5'-phosphate glycosidase (EC 4.2.1.70)	Yes	No
		Purine nucleoside phosphorylase (EC 2.4.2.1)	No	Yes

		Ribonucleotide reductase of class Ia (aerobic), alpha subunit (EC 1.17.4.1)	Yes	No
		Ribonucleotide reductase of class Ia (aerobic), beta subunit (EC 1.17.4.1)	Yes	No
55	Pyruvate metabolism	Dihydrolipoamide dehydrogenase (EC 1.8.1.4); Dihydrolipoamide dehydrogenase of 2-oxoglutarate dehydrogenase (EC 1.8.1.4)	Yes	No
		D-Lactate dehydrogenase, cytochrome c-dependent (EC 1.1.2.4)	Yes	No
		Homocitrate synthase (EC 2.3.3.14)	Yes	No
		Hydroxyacylglutathione hydrolase (EC 3.1.2.6)	No	Yes
		Lactoylglutathione lyase (EC 4.4.1.5)	Yes	No
		Malate synthase (EC 2.3.3.9)	Yes	No
		Phosphoenolpyruvate carboxykinase [ATP] (EC 4.1.1.49)	Yes	No
		Pyruvate carboxylase (EC 6.4.1.1)	Yes	No
		Pyruvate dehydrogenase E1 component alpha subunit (EC 1.2.4.1)	Yes	No
56	Reductive carboxylate cycle (CO2 fixation)	Fumarate hydratase class II (EC 4.2.1.2)	Yes	No
		Acetyl-CoA synthetase (EC 6.2.1.1)	Yes	No
		Biotin carboxylase of acetyl-CoA carboxylase (EC 6.3.4.14) / Biotin carboxyl carrier protein of acetyl-CoA carboxylase / Acetyl-coenzyme A carboxyl transferase (EC 6.4.1.2)	Yes	No
		Aconitate hydratase (EC 4.2.1.3)	Yes	No
		Formate--tetrahydrofolate ligase (EC 6.3.4.3)	Yes	No
		Formate--tetrahydrofolate ligase (EC 6.3.4.3)	Yes	No
57	Retinol metabolism	Alcohol dehydrogenase (EC 1.1.1.1)	No	Yes
58	Riboflavin metabolism	6,7-dimethyl-8-ribityllumazine synthase (EC 2.5.1.78)	Yes	No
		3,4-dihydroxy-2-butanone 4-phosphate synthase (EC 4.1.99.12) / GTP cyclohydrolase II (EC 3.5.4.25)	Yes	No
		GTP cyclohydrolase II (EC 3.5.4.25)	Yes	No
		Riboflavin synthase (EC 2.5.1.9)	Yes	No
59	Selenoamino acid metabolism	Methionyl-tRNA synthetase (EC 6.1.1.10)	Yes	Yes
		Cystathionine gamma-lyase (EC 4.4.1.1)	Yes	No
		Thioredoxin reductase (EC 1.8.1.9)	No	Yes
		Acyl-CoA-dependent ceramide synthase (EC 2.3.1.24)	Yes	No
		Serine palmitoyltransferase, subunit LCB2 (EC 2.3.1.50)	Yes	No

60	Sphingolipid metabolism	Sphingoid long chain base kinase (EC 2.7.1.91)	No	Yes
		Sphingomyelinase (EC 3.1.4.12)	Yes	No
		Sphingosine-1-phosphate lyase (EC 4.1.2.27)	Yes	No
61	Starch and sucrose metabolism	4-alpha-glucanotransferase (amylomaltase) (EC 2.4.1.25) / Amylo-alpha-1,6-glucosidase (EC 3.2.1.33)	Yes	No
		Alpha,alpha-trehalose-phosphate synthase [UDP-forming] (EC 2.4.1.15)	Yes	Yes
		Alpha,alpha-trehalose-phosphate synthase [UDP-forming] (EC 2.4.1.15)	No	Yes
		beta-glucosidase (EC 3.2.1.21)	No	Yes
		Glucan 1,3-beta-glucosidase (EC 3.2.1.58)	Yes	No
		Glucose-6-phosphate isomerase (EC 5.3.1.9)	No	Yes
		Glycogen [starch] synthase, (EC 2.4.1.11)	Yes	No
		Hexokinase (EC 2.7.1.1)	Yes	No
		Oligo-1,6-glucosidase (EC 3.2.1.10)	Yes	Yes
		Trehalase (EC 3.2.1.28)	No	Yes
62	Steroid biosynthesis	Sterol 24-C-methyltransferase (EC 2.1.1.41)	Yes	No
		Sterol O-acyltransferase (EC 2.3.1.26)	No	Yes
63	Sulfur metabolism	Adenylylsulfate kinase (EC 2.7.1.25)	No	Yes
		Phosphoadenylyl-sulfate reductase [thioredoxin] (EC 1.8.4.8)	Yes	No
		Sulfite reductase [NADPH] flavoprotein alpha-component (EC 1.8.1.2)	Yes	No
64	T cell receptor signaling pathway	Serine/threonine protein phosphatase (EC 3.1.3.16)	Yes	Yes
65	Terpenoid backbone biosynthesis	Dehydrodolichyl diphosphate synthase (EC 2.5.1.87)	Yes	No
		Dimethylallyltransferase (EC 2.5.1.1) / (2E,6E)-farnesyl diphosphate synthase (EC 2.5.1.10) / Farnesyltranstransferase (EC 2.5.1.29)	Yes	No
		Diphosphomevalonate decarboxylase (EC 4.1.1.33)	Yes	No
		Hydroxymethylglutaryl-CoA reductase (EC 1.1.1.34)	Yes	No
		Isopentenyl-diphosphate Delta-isomerase (EC 5.3.3.2)	No	Yes
		Mevalonate kinase (EC 2.7.1.36)	Yes	No
		Octaprenyl diphosphate synthase (EC 2.5.1.90)	No	Yes
66	Tetrachloroethene degradation	Alcohol dehydrogenase (EC 1.1.1.1)	Yes	No

67	Thiamine metabolism	Thiamin-phosphate pyrophosphorylase (EC 2.5.1.3)	Yes	No
		Hydroxymethylpyrimidine phosphate kinase ThiD (EC 2.7.4.7)	No	Yes
68	Tryptophan metabolism	Indoleamine 2,3-dioxygenase (EC 1.13.11.52)	Yes	No
		Kynurenine 3-monooxygenase (EC 1.14.13.9)	Yes	No
		Kynureninase (EC 3.7.1.3)	No	Yes
69	Tyrosine metabolism	4-hydroxyphenylpyruvate dioxygenase (EC 1.13.11.27)	No	Yes
70	Ubiquinone and other terpenoid-quinone biosynthesis	NAD(P)H dehydrogenase (quinone), Type IV (EC 1.6.5.2)	Yes	No
		4-hydroxybenzoate polyprenyltransferase (EC 2.5.1.39)	No	Yes
71	Valine, leucine and isoleucine biosynthesis	Dihydroxy-acid dehydratase (EC 4.2.1.9)	Yes	No
		2-isopropylmalate synthase (EC 2.3.3.13)	Yes	No
		Acetolactate synthase small subunit (EC 2.2.1.6)	Yes	No
		3-isopropylmalate dehydratase large subunit (EC 4.2.1.33)	Yes	No
72	Valine, leucine and isoleucine degradation	Aldehyde dehydrogenase (EC 1.2.1.3)	Yes	No
		Methylmalonate-semialdehyde dehydrogenase (EC 1.2.1.27)	Yes	No
		3-hydroxyisobutyryl-CoA hydrolase (EC 3.1.2.4)	No	Yes
73	Vitamin B6 metabolism	Phosphoserine aminotransferase (EC 2.6.1.52)	Yes	Yes
		Pyridoxal kinase (EC 2.7.1.35)	Yes	Yes
74	Zeatin biosynthesis	tRNA dimethylallyltransferase (EC 2.5.1.75)	Yes	No
